# Bio‐Based Polyurethanes for Sustainable and Multifunctional Applications

**DOI:** 10.1002/advs.77078

**Published:** 2026-08-11

**Authors:** Xin Li, Henghui Deng, Shiwei Yu, Jiawei Huang, Zehong Chen, Dawei Zhao, Chaoqun Zhang

**Affiliations:** ^1^ College of Future Biomass South China Agricultural University Guangzhou People's Republic of China; ^2^ Key Laboratory on Resources Chemicals and Materials of Ministry of Education Shenyang University of Chemical Technology Shenyang People's Republic of China; ^3^ State Key Laboratory of Woody Oil Resources Utilization Northeast Forestry University Harbin People's Republic of China; ^4^ College of Material Science and Art Design Inner Mongolia Agricultural University Hohhot People's Republic of China

**Keywords:** bio‐based polyurethane, dynamic polymer network, smart materials, structural engineering, sustainable materials

## Abstract

Bio‐based polyurethanes, derived from renewable feedstocks such as vegetable oils, lignin, and polysaccharides, have attracted increasing attention as sustainable alternatives to fossil‐derived polyurethanes and as versatile materials for smart coatings, flexible electronics, biomedical engineering, and energy devices. Through structural engineering strategies combining dynamic covalent network design with nanocomposite reinforcement, these materials achieve mechanical tunability, reprocessability, and multifunctionality. This review summarizes recent progress in bio‐based polyurethane chemistry, encompassing bio‐polyols, internal emulsifiers, isocyanates, and non‐isocyanate synthetic routes, and discusses structural engineering strategies based on chain‐segment design, aggregation‐state control, dynamic covalent bond chemistry, and nanocomposite reinforcement. These strategies overcome the inherent limitations of thermosets, enabling high strength, self‐healing, and recyclability, thereby supporting applications in protective coatings, fire‐warning systems, tissue repair, antibacterial dressings, flexible sensors, and energy storage devices. This review examines remaining challenges and future opportunities, including sustainability assessment, scalable processing, end‐of‐life management, and data‐driven material design, toward the development of sustainable, reliable, and closed‐loop bio‐based polyurethane systems.

## Graphical abstract

Bio‐based polyurethanes prepared from vegetable oils, lignin, and polysaccharides have attracted increasing interest as alternatives to fossil‐derived polyurethanes. This review summarizes recent progress in their chemistry, structural engineering, and advanced applications, highlighting the roles of feedstocks, chain‐segment design, dynamic covalent networks, and nanocomposite interfaces in regulating mechanical performance, self‐healing, reprocessability, and multifunctionality. Remaining challenges in sustainability assessment, scalable processing, and end‐of‐life management are also discussed.

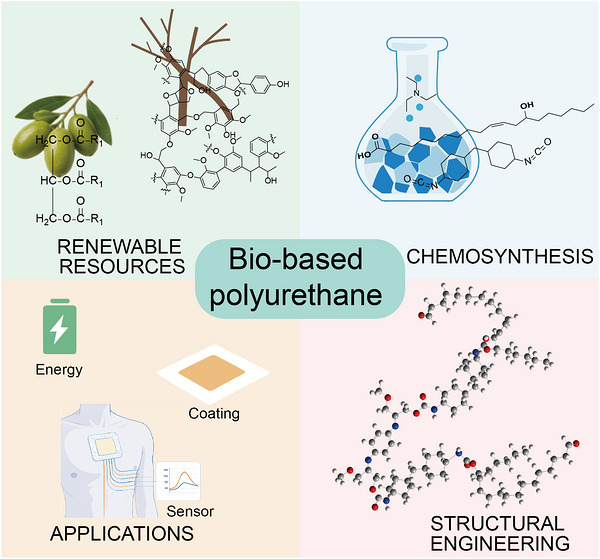

## 1 Introduction

Escalating environmental concerns, the depletion of fossil resources, and increasingly stringent regulatory frameworks have driven the imperative to replace petroleum‐based feedstocks with renewable raw materials in polymer production [1, 2, 3]. Annual global production of polyurethane (PU) has exceeded 27 million tons [4, 5], and the accumulation of persistent PU waste in the environment is becoming an increasingly serious problem, while the non‐renewable nature of fossil resources has compelled the industry to seek fundamental alternatives [6, 7, 8]. In this context, bio‐based PU, which employs renewable raw materials to construct polymer backbones, has emerged as an active research field with the potential to simultaneously alleviate the dual pressures of resource dependence and waste management [9, 10, 11]. However, it is important to emphasize that while bio‐based raw materials reduce dependence on fossil resources, the overall sustainability of bio‐based PUs depends on multiple factors beyond feedstock origin, including the energy intensity of chemical modification processes, agricultural land‐use impacts, water consumption, and end‐of‐life management option [12, 13, 14]. A rigorous life‐cycle assessment (LCA) framework is therefore essential for substantiating sustainability claims, as discussed in Section 5.1.1. At the same time, transitioning from simple raw material substitution to intelligent applications requires overcoming two key challenges: reduced mechanical properties and limited functional expression. The substantially greater chemical diversity of bio‐based raw materials compared with their petroleum‐based counterparts, exemplified by long aliphatic chains, aromatic rings, and multi‐functional groups, presents both opportunities and challenges for polymer design [15, 16, 17]. Transforming these structural features into controllable condensed‐matter structures and dynamic response functions is the central focus of current research [18, 19].

The properties of PU are typically governed by the soft‐to‐hard segment ratio and the microphase separation structure, where soft segments provide flexibility and elasticity, while hard segments impart mechanical strength and thermal stability through hydrogen‐bond aggregation [20]. The introduction of bio‐based polyols, which can be derived from vegetable oils, lignin, polysaccharides, and microalgae oils, has opened new chemical pathways for soft‐segment design using polyol precursors of varying functionality [4, 21, 22]. For instance, castor oil can directly participate in polymerization owing to its inherent hydroxyl functional groups, whereas soybean oil and palm oil require the introduction of reactive sites via epoxidation or ozonation. The synthesis of bio‐based isocyanates is more challenging; both phosgenation and non‐phosgenation routes have been employed to convert vegetable oils into isocyanate‐terminated prepolymers [23, 24]. The non‐isocyanate polyurethane (NIPU) route constructs networks through the aminolysis of cyclic carbonates with polyamines, completely circumventing the use of toxic isocyanates. These chemical pathways provide an important foundation for reducing fossil‐resource dependence. Traditional bio‐based PU networks are predominantly thermosetting structures with permanent cross‐links; once formed, such materials cannot be reprocessed or self‐repaired [25, 26]. This static characteristic severely limits their integration into smart coatings, biomedical engineering, and flexible electronic devices. In recent years, the introduction of dynamic covalent chemistry has provided a strategy to address this limitation [27, 28]. Researchers have embedded reversible units such as disulfide, imine, acylhydrazone, and oxime‐carbamate bonds into the main chains of bio‐based PUs [29, 30]. These dynamic bonds undergo reversible cleavage and recombination in response to thermal, photonic, or pH stimuli, endowing the materials with self‐healing, reprocessing, and stimuli‐responsive capabilities [31, 32]. The dynamic covalent chemistry‐based approach fundamentally transforms the network topology of bio‐based PUs from a static, permanently cross‐linked architecture into a dynamic, adaptable architecture. Notably, bio‐based monomers with inherent multi‐functional groups enable higher cross‐linking densities and more complex dynamic bond combinations compared with their petroleum‐based counterparts [33]. This unique chemical versatility makes bio‐based PUs a particularly promising platform for implementing dynamic covalent chemistry to construct intelligent polymer networks.

Another critical dimension of structural engineering is the incorporation of nanomaterials. The interfacial compatibility between the bio‐based PU matrix and functional nanofillers can be optimized through polar‐group anchoring and hydrogen‐bonding networks [34, 35, 36]. The intrinsic high strength of nanofillers can not only effectively modulate mechanical properties but also impart additional functionalities [37]. For example, cellulose nanocrystals (CNCs) can form a rigid percolation network that reinforces the matrix, boron nitride nanosheets (BNNSs) can be oriented to construct efficient thermal conduction pathways, and conductive carbon black (CB) or silver nanowire percolation networks can endow strain‐sensing functionality. Importantly, bio‐based components serve not merely as dispersion media during nanocomposite fabrication, but also actively participate in interfacial stress transfer and dynamic bond rearrangement through their structural features.

These molecular‐ and mesoscale structural engineering advances are driving bio‐based PUs toward multifunctional, integrated applications. In the field of coatings and corrosion protection, the incorporation of bio‐based rigid monomers simultaneously increases cross‐linking and hydrogen‐bond network densities, thereby enhancing the barrier properties of the material. In fire protection, phosphorus‐containing polyols derived from phytic acid, lignin, and vanillin are covalently incorporated into the network, thereby stabilizing the flame‐retardant elements within the polymer matrix. The synergistic effects of condensed‐phase charring and gas‐phase free‐radical quenching enable the material to achieve high‐efficiency flame retardancy while preserving mechanical properties [9]. In biomedical engineering, the programmable characteristics of the soft and hard segments of bio‐based PU enable a mechanical response spanning from soft elasticity to strong plasticity. In flexible sensing and energy devices, bio‐based dynamic networks maintain the stability of the conductive pathways within electrodes under repeated deformation [38, 39, 40]. Self‐healing current collectors and high‐dielectric‐constant dielectrics have enabled the development of sustainable energy storage devices.

**FIGURE 1:**
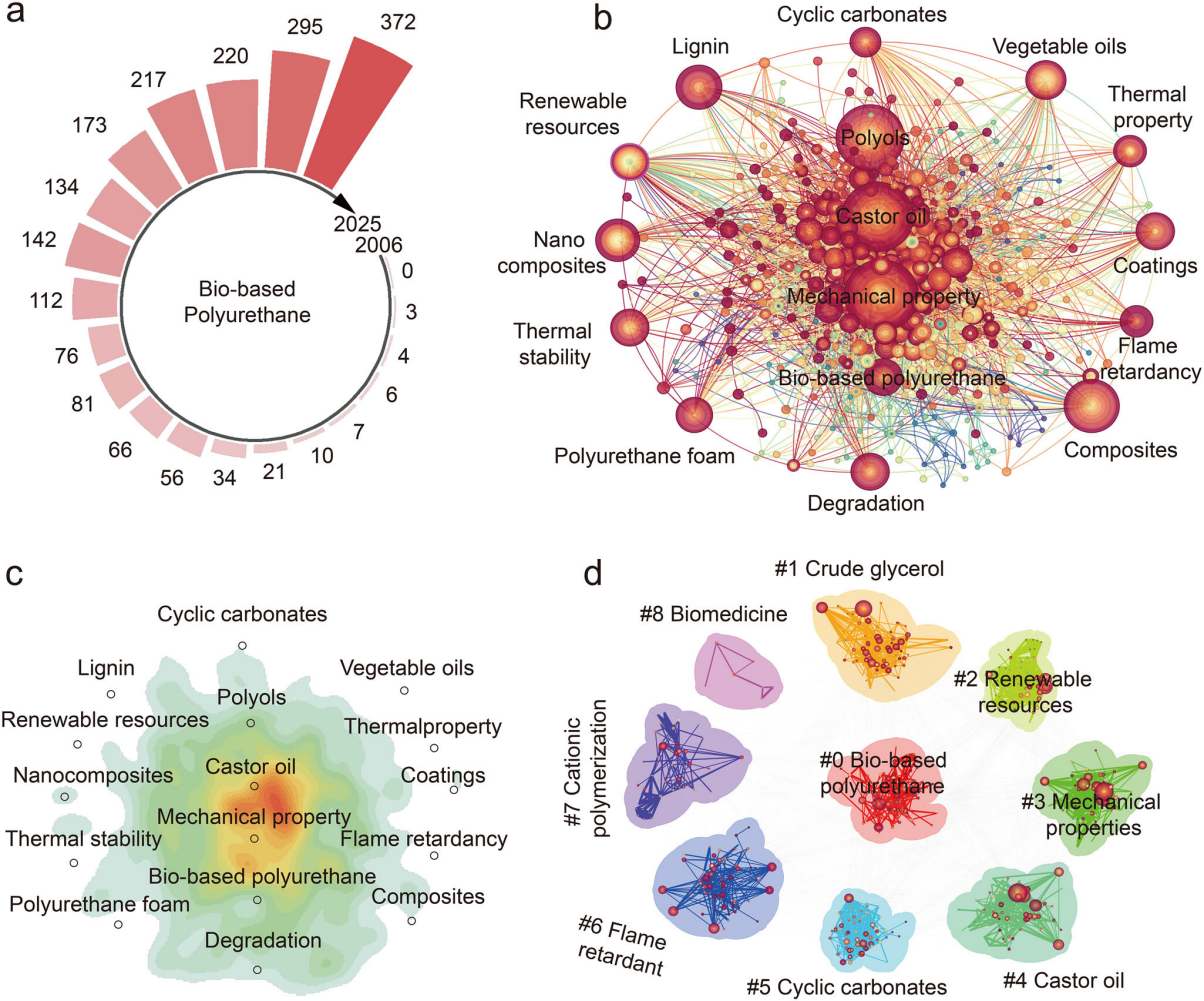
Analysis of publications from 2006 to 2025. (a) Annual publications involving bio‐based polyurethane. (b) Keyword network visualization related to the development of bio‐based polyurethane in the literature. (c) Heatmap of keywords for publications involving bio‐based polyurethane. (d) Keyword clustering of publications involving bio‐based polyurethane.

Bibliometric analysis of the Web of Science database (2006–2025) reveals that the number of publications containing “bio‐based” and “PU” keywords has increased steadily year by year (Figure 1a). Based on CiteSpace 6.4 analysis of 2029 articles, high‐frequency research topics were identified (Figure 1b,c). Mechanical properties, castor oil, and polyol are the three most frequent keywords, indicating that research on bio‐based PU has long centered on optimizing bio‐based raw materials and mechanical performance. Concurrently, the emergence of keywords such as composite materials, flame retardancy, and degradability reflects a growing research trend toward sustainability and functional orientation. The keyword clustering results delineate the current research framework in the field of bio‐based PU (Figure 1d). The core cluster is “#0 bio‐based PU.” Research primarily focuses on three dimensions: raw material development (#1 crude glycerol, #4 castor oil, and #5 cyclic carbonates), sustainability orientation (#2 renewable resources), and performance enhancement and functionalization (#3 mechanical properties, #6 flame retardant). Additionally, clusters such as #7 cationic polymerization and #8 biomedicine point to emerging subfield directions, including synthetic methods and specific bioactive components. Overall, the field has established a coherent knowledge chain spanning the acquisition of renewable raw materials, green synthesis, and the construction of multifunctional materials.

**FIGURE 2:**
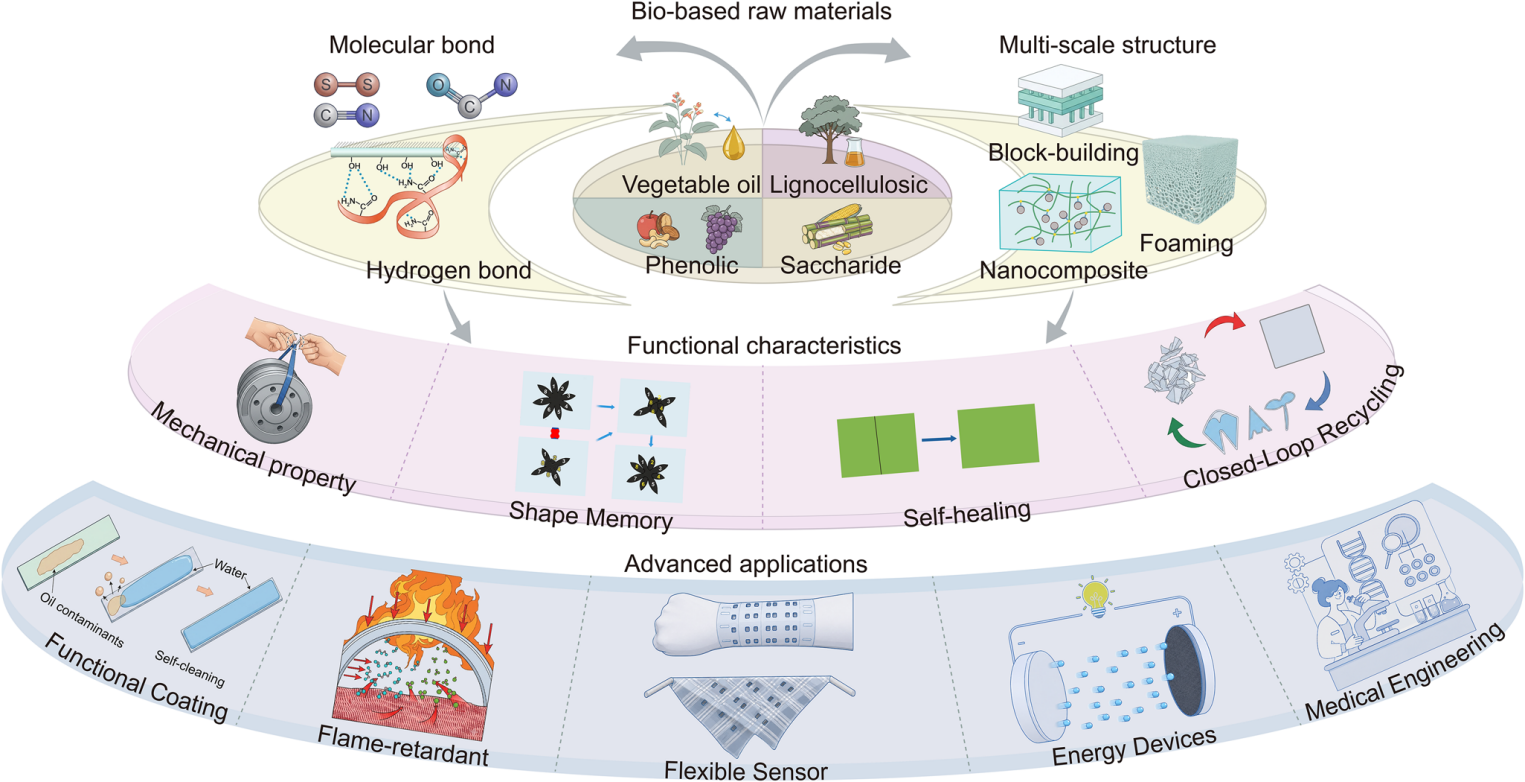
An overview of the scope of this review highlighting the main topics discussed in each section.

In summary, research on bio‐based PU has progressed from raw‐material substitution to multi‐scale, structure‐driven functional integration. From chain‐segment composition design to aggregation morphology regulation, from dynamic covalent network topology to nanocomposite interface optimization, structural innovation at each level continues to expand the functional boundaries of bio‐based PU materials. This review systematically summarizes the latest progress of bio‐based PU in chemical basic synthesis, structural engineering design, and advanced applications (Figure 2). Compared with reviews focused mainly on specific feedstocks, specific functions, or individual applications, this review integrates three interconnected dimensions into a holistic framework [20, 41, 42, 43]: (1) multi‐scale structural engineering strategies spanning from molecular chain‐segment design to nanocomposite interfaces, (2) a systematic critical comparison of dynamic covalent bond chemistries in bio‐based PU networks, and (3) structure‐performance‐function relationships across representative application areas. By examining these dimensions together, we aim to elucidate the intrinsic relationships among structure, performance, and function, and to identify remaining challenges and outline future research directions for next‐generation functional materials.

## 2 Chemical Basis of Bio‐Based Polyurethane

To realize the transformation of bio‐based PU from simple raw material substitutes to high‐strength, high‐toughness, and multifunctional materials, a thorough understanding of its chemical basis is essential, as this forms the foundation for structural engineering design. PU is a block copolymer composed of soft segments and hard segments connected through carbamate bonds. This chemical architecture endows PU with tunable mechanical properties, molecular designability, excellent wear resistance, and broad applicability [44]. The core strategy of bio‐based PU production is to replace traditional petroleum‐based raw materials with renewable resources (Table 1). One approach is to synthesize PU using bio‐based polyols of varying functionality together with bio‐based isocyanates; another is to construct PU via the NIPU route, which circumvents isocyanate usage entirely.

**TABLE 1:** Raw material sources and characteristics of bio‐based polyurethane.

Feedstock category	Polyol/isocyanate type	Key structural features	Key references
Castor Oil [Natural polyol]	Castor oil (OH functionality ∼ 2.7) Ricinoleic acid‐derived	Naturally occurring OH groups Long aliphatic C18 backbone Direct use	[45]
Soybean Oil [Chemically modified]	Epoxidized + ring‐opened Methoxylated soybean Acrylated epoxidized	ring‐opening to introduce OH groups High unsaturation Tunable OH value	[46]
Lignin‐based Polyol [Aromatic polyol]	Kraft lignin Oxypropylated lignin Lignin‐derived aromatic	Aromatic ring provide rigidity and thermal stability Abundant phenolic and aliphatic OH groups Acts as both polyol and hard segment contributor	[47]
Carbohydrate/Polysaccharide [Polyether polyol]	Sorbitol/glycerol Isosorbide‐based Sucrose/glucose‐based	High OH functionality Rigid cyclic structures suitable for waterborne systems Non‐toxic, food‐grade origin	[48]
Bio‐based Isocyanates	PDI (pentamethylene diisocyanate from lysine) Partially bio‐based MDI(methylenediphenyl diisocyanates)/TDI(toluene diisocyanate) Fatty acid derived diisocyanate	PDI: linear C5 aliphatic chain, reactivity similar to HDI Lysine‐derived: 100% bio‐carbon Fatty acid derived diisocyanate: long chain → hydrophobicity Limited commercial availability and higher cost currently	[49]
NIPU	Cyclic carbonate (from CO_2_ + epoxide) Polyhydroxyurethane from bis‐cyclic carbonate + diamine	Eliminates toxic isocyanate use Inherent OH along backbone → enhanced adhesion CO_2_ utilization as C1 feedstock Generally lower MW and mechanical strength vs. conventional PU	[50]
Internal Emulsifier [Bio‐based waterborne polyurethane]	2,2‐dimethylolpropionic acid (DMPA) / 2,2‐dimethylolbutyric acid (DMBA) (petroleum‐based, widely used) CO_2_‐adduct hydrophilic polyol Sulfonated bio‐based polyol	DMPA: industry standard, provides COOH for neutralization Bio‐based alternatives: citric acid, tartaric acid derivatives CO_2_‐adduct: epoxide → cyclic carbonate → hydrophilic segments	[51]

### 2.1 Bio‐Based Polyol

**FIGURE 3:**
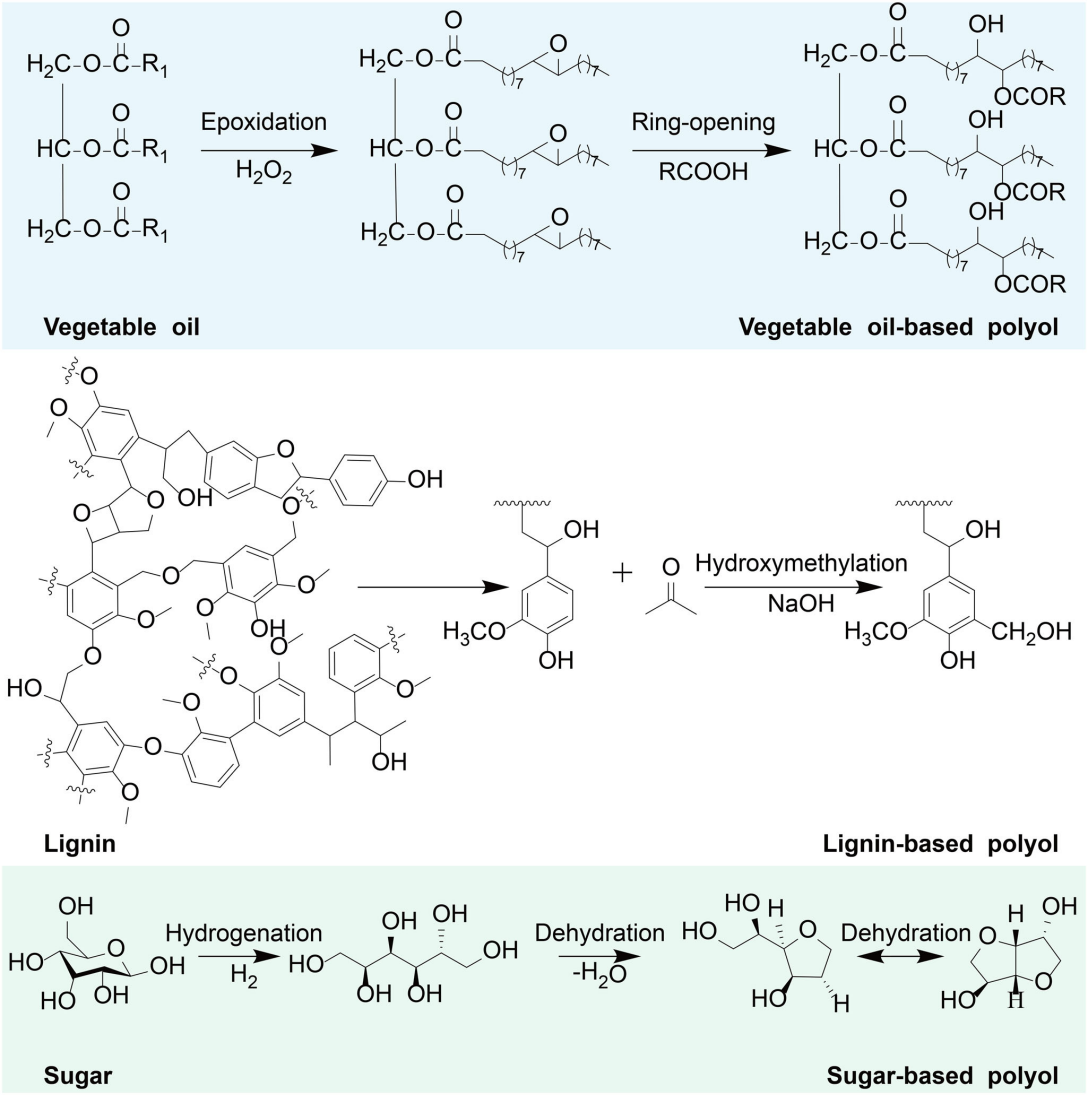
A schematic diagram of bio‐based polyol preparation route.

Polyols are the key components determining the performance of PU [52, 53]. The scientific and industrial communities have long been engaged in developing and applying novel bio‐based polyols for PU production. Various renewable resources, including vegetable oils, lignin, polysaccharides, and microalgae, have been explored for the preparation of bio‐based polyols (Figure 3) [54, 55]. These biomass feedstocks possess diverse chemical structures, enabling the production of polyols with a wide range of properties. For example, vegetable oils are typically used to produce polyester polyols, polysaccharides are primarily used to produce polyether polyols, and lignin is used to produce rigid polyols with aromatic ring structures [56].

Vegetable oils contain long‐chain unsaturated fatty acid structures that meet the fundamental chemical requirements for the development of bio‐based polyols; accordingly, they are favored by both academia and industry. As early as 2008, Ford Motor Company announced the development of soybean oil‐based polyols for the synthesis of bio‐based flexible PU foam and applied this foam in their vehicle interiors. Castor oil is one of the few raw materials that can be used directly as a polyol for producing bio‐based PU [57]. Its molecular structure contains approximately 2.7 hydroxyl groups per molecule, allowing it to participate directly in polymerization as a polyol without complex chemical modification. Its long‐chain fatty acid structure imparts flexibility to PU products while also conferring favorable hydrolysis resistance and mechanical properties. Consequently, industry has shown a strong preference for developing castor oil‐based polyols for synthesizing bio‐based PU. Except for ricinoleic acid, most fatty acid molecular structures lack hydroxyl groups; therefore, these fatty acid chains must undergo hydroxyl functionalization before they can serve as polyols.

**FIGURE 4:**
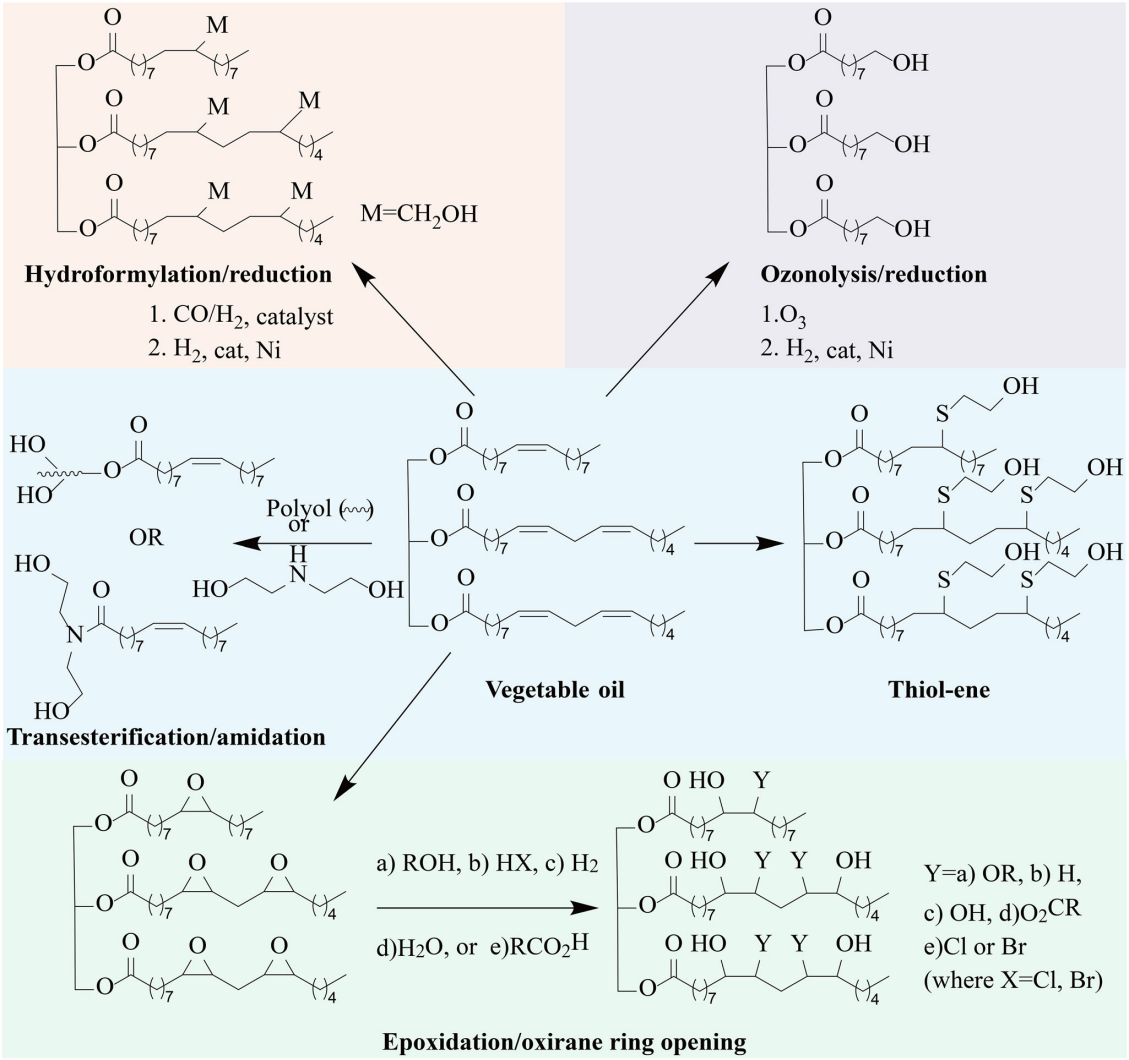
A schematic diagram of typical preparation methods for vegetable oil‐based polyol.

Currently, various chemical methods have been employed to develop vegetable oil‐based polyols, including epoxidation/oxirane ring‐opening, ozonolysis/reduction, hydroformylation/hydrogenation, transesterification/amidation, and thiol‐ene reactions (Figure 4) [46, 58]. Zeng et al. [59] synthesized diols via the amidation reaction of palm oil with diethanolamine, and the resulting diol had a palmitoleic acid side chain. They used 1,4‑butanediol to construct PU elastomers synergistically, obtaining an elastomer rich in hydrogen bonds that exhibited a highly microphase‑separated structure. In another study, Becquet et al. [60] successfully synthesized bio‐based polyol using a rhodium catalyst in a hydrogen‐carbon monoxide atmosphere, via consecutive hydroformylation and hydrogenation of the carbon‐carbon double bonds in castor oil and ethyl ricinoleate. Feng et al. [61] employed a self‑built photochemical reactor to modify soybean oil through the thiol‑ene click reaction under light; this reaction successfully yielded bio‑based polyols, which were then utilized for the synthesis of high‑strength PU materials.

As the second most abundant natural polymer, lignin constitutes 15 to 35% of lignocellulosic biomass and remains the only renewable aromatic polymer available on a large scale [62, 63]. Its molecular framework is built upon phenylpropane units and contains a high density of phenolic and aliphatic hydroxyl groups, which serve as the essential reactive sites for PU synthesis. Biosynthetically, lignin arises from the coupling polymerization of p‐coumaryl, coniferyl, and sinapyl alcohols, yielding p‑hydroxyphenyl, guaiacyl, and syringyl subunits, respectively [64]. The relative abundance of these three unit types varies considerably among plant sources: softwood lignin consists predominantly of guaiacyl moieties, hardwood lignin derives from the copolymerization of guaiacyl and syringyl units, and grass lignin incorporates all three [65]. Notably, an elevated syringyl content correlates with fewer free substitution positions on the aromatic ring and consequently diminished reactivity [66]. This inherent structural variation underpins all subsequent strategies for preparing lignin‑derived polyols.

Although methods for developing bio‐based PU from vegetable oils have been extensively explored, the epoxidation or ring‐opening approach remains the most widely adopted in industry due to its simplicity. Rapeseed oil, palm oil, and soybean oil are the most abundant and cost‐effective oils in the world, and these oils are most suitable for large‐scale production of bio‐based polyols. However, despite their abundance, these vegetable oils are essentially edible raw materials, and their large‐scale industrial use raises concerns about competition with food supplies [67]. Given that their production capacity rivals that of vegetable oils and their conversion into polyols follows analogous synthetic routes, microalgal oils have recently attracted considerable attention, affording products with hydroxyl values as high as 150 mg KOH g^−^^1^ [68].

Carbohydrate modification is usually carried out using the epoxy ring‐opening method, which involves reacting compounds containing hydroxyl or amino groups in the presence of a catalyst [69, 70]. Biomass such as starch, chitosan, and alginate can be converted into bio‐based polyols through this modification; they are natural polymers formed by monosaccharides linked by glycosidic bonds and contain various functional groups such as amino, carboxyl, or sulfonic groups [71]. Polysaccharide‐based polyols can effectively enhance the degradability of PU materials [72, 73]. Among these, starch is the most extensively studied polysaccharide‐based polyol, which is composed of amylose and amylopectin linked by *α*‐D_1,4_‐ and *α*‐D_1,6_‐glycosidic bonds. Starch can be reacted directly with an oligomeric diisocyanate to synthesize cross‐linked PU, which usually exhibits relatively low thermal decomposition temperature and high hydrophilicity [74]. Chitosan is commonly used in the synthesis of PU, as hydroxyl and amino groups on its main chain can react with isocyanate to prepare poly(urea‐urethane) materials, which usually exhibit thermal stability and possess inherent antibacterial and antifungal properties. Additionally, these polysaccharide biomasses can be chemically converted into small‐molecule diols, such as bio‐based 1,4‐butanediol, which are often utilized as chain extenders [75]. In addition to being converted into polyols, these biomass resources can also be transformed into polyamines, which are primarily used as curing agents for polyurea or NIPU [76].

Despite these advances, it is important to recognize that natural variations in feedstock composition pose significant challenges for industrial‐scale production. Vegetable oils exhibit batch‐to‐batch variability in hydroxyl value, fatty acid composition, and molecular weight distribution, depending on plant species, growing conditions, and harvest seasons [77]. Similarly, lignin structure and reactivity vary substantially with botanical source (softwood, hardwood, grass lignin) and isolation process employed [78]. These variations can disrupt the NCO/OH stoichiometric balance, alter curing kinetics and viscosity evolution, and change cross‐link density or microphase separation behavior, thereby causing unstable gel times, foaming defects, coating irregularities, or inconsistent mechanical properties in mass‐produced foams and coatings. Addressing this variability requires, first, establishing feedstock quality specifications based on hydroxyl value, acid value, viscosity, and molecular weight distribution to ensure consistent reactivity. When specifications alone are insufficient, blending of different feedstock batches can average out compositional variations, and real‐time process monitoring with adaptive formulation adjustment can compensate for residual variability.

### 2.2 Bio‐Based Internal Emulsifier

Internal emulsifiers are essential components required for the preparation of waterborne polyurethane (WPU) [79]. In the existing industrial system, 2,2‐dimethylolpropionic acid and 2,2‐dimethylolbutyric acid are the most commonly used commercial internal emulsifiers due to their mature industrial processes and stable performance. These molecules contain multiple hydroxyl and carboxylic acid functional groups. To retain their renewable credentials while rivaling or surpassing the emulsification efficiency of petroleum‐derived counterparts, bio‐based internal emulsifiers must be developed with enhanced performance characteristics, a prerequisite for the successful synthesis of bio‐based WPU [80, 81].

**FIGURE 5:**
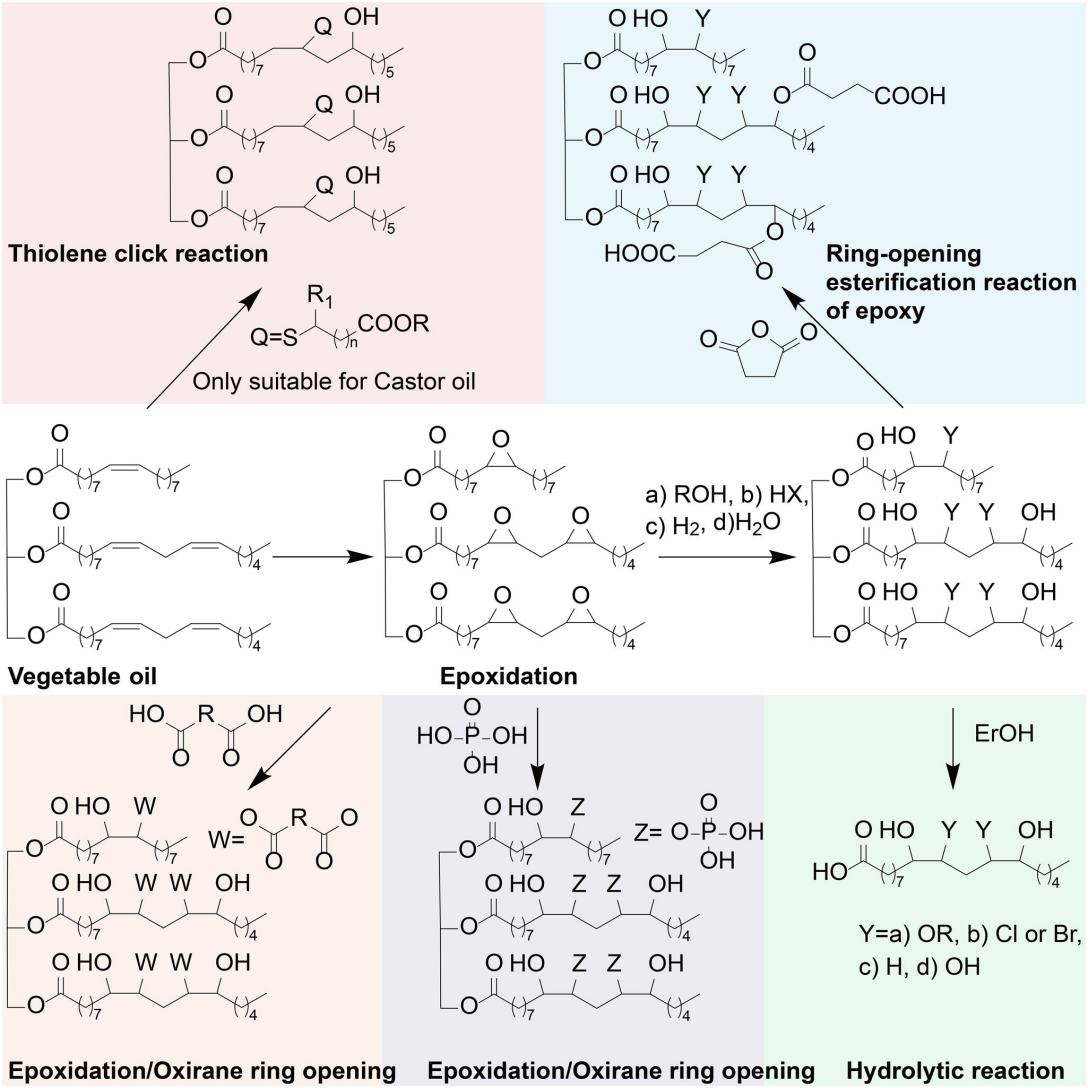
A schematic diagram of typical preparation methods for vegetable oil‐based internal emulsifiers.

Currently, bio‐based internal emulsifiers include vegetable oil‐based internal emulsifiers and some small‐molecule aliphatic acids. Among them, vegetable oil‐based internal emulsifiers can be synthesized through the following methods (Figure 5) [82, 83, 84, 85]: (1) Polyhydroxy vegetable oils obtained via epoxidation and subsequent ring opening at the carbon‐carbon double bonds of native vegetable oils can be saponified to yield internal emulsifiers characterized by a single fatty acid chain architecture [86]. (2) Vegetable oil‐based internal emulsifiers are prepared via a two‐step functionalization sequence in which hydroxyl groups are first introduced onto the aliphatic backbone through epoxidation and ring opening, and a subsequent partial ring opening of an anhydride by these pendant hydroxyls installs the requisite carboxyl moieties [87]. (3) Thioglycolic acid can be grafted onto the molecular backbone of castor oil via thiol‐ene photo click reactions to yield vegetable oil‐based internal emulsifiers. However, this strategy remains exclusive to castor oil owing to its coexistence of both carbon‐carbon double bonds and hydroxyl groups. (4) The ring‐opening reaction of epoxidized vegetable oils can directly prepare internal emulsifiers in a single step, which uses small‐molecule acids, such as phosphoric acid, fatty acids, or polycarboxylic acids [88]. In addition, some small‐molecule hydroxy acids, such as tartaric acid, can be directly used as bio‐based internal emulsifiers.

### 2.3 Bio‐Based Isocyanate

Compared with the vigorous development of polyols, the development of bio‐based isocyanates is more challenging. Currently, the academic community has conducted exploratory research on the bio‐based synthesis of isocyanates using vegetable oils, including castor oil, soybean oil, linseed oil, and palm oil. The preparation of vegetable oil‐based isocyanates involves multi‐step chemical reactions. The core step of this process is to convert natural oils rich in unsaturated fatty acids into vegetable‐oil‐based polyamines via chemical conversion. These bio‑based intermediates can then be transformed into target products via either the traditional phosgenation process or more environmentally friendly non‑phosgene processes, such as dimethyl carbonate or carbamate pyrolysis [89, 90]. These methods can connect the highly reactive isocyanate groups to the end of molecular chains, finally producing vegetable oil‑based isocyanates. For example, Çaylı and Küsefoğlu [91] used N‐bromosuccinimide to brominate the carbon‐carbon double bond of soybean oil, followed by a substitution reaction with AgNCO to convert bromine atoms into NCO groups. They also proposed a simpler method in which AgNCO and iodine were dissolved in tetrahydrofuran, and soybean oil was added; the mixture was allowed to react at room temperature for 2 h to obtain the target soybean oil‑based isocyanate. Phung Hai et al. [92] extracted palmitoleic acid from algae, performed ozonolysis to produce azelaic acid, and converted it into dimethyl azelate. The dimethyl azelate was then transformed into azelodihydrazide using hydrazine hydrate, and the acyl azide was prepared by the reaction of azelodihydrazide with NaNO_2_·HCl at 0°C. Subsequently, the acyl azide was heated to 85°C, yielding a terminal fatty acid diisocyanate whose performance is comparable to that of 1,6‐hexamethylene diisocyanate. Furthermore, More et al. [93] used the hydrazide‐acyl azide reaction to treat undecylenic acid derived from castor oil, and the resulting product was subsequently converted into a terminal bio‐based diisocyanate via a Curtius rearrangement. To completely avoid the use of toxic isocyanates, the NIPU route has been gradually explored. Among the explored NIPU routes, the most classic one is to epoxidize these biomass resources, after which the epoxy product is converted into a bio‑based cyclic carbonate via a CO_2_ addition reaction, and the cyclic carbonate is polymerized with amine compounds to form an NIPU.

### 2.4 Construction of Bio‐Based Polyurethane Network

As mentioned above, the synthesis of bio‐based PU relies on the extensive development and use of renewable raw materials. Polyols, isocyanates, and internal emulsifiers necessary for WPU systems together constitute the three core components of their chemical networks [94]. Understanding how these components assemble into a cross‐linking network through polymerization is the basis for further exploration of structural engineering design and, ultimately, for endowing materials with superior mechanical properties and intelligence.

From a chemical composition perspective, the network structure of bio‑based PU consists primarily of soft and hard segments. The soft segments are usually derived from long‑chain polyols that are obtained from vegetable oils, microalgae oils, or polysaccharides. These components endow the material with flexibility and ductility. The hard segments are formed by the reaction of an isocyanate with a small‑molecule chain extender, which imparts strength, modulus, and heat resistance to the material. When bio‑based isocyanates are used, the bio‑based content of the entire material can be further increased. In the WPU system, the introduction of bio‑based internal emulsifiers enables hydrophobic polymer chains to be stably dispersed in water, thereby avoiding the use of large volumes of organic solvents, a key step toward cleaner production and application.

**FIGURE 6:**
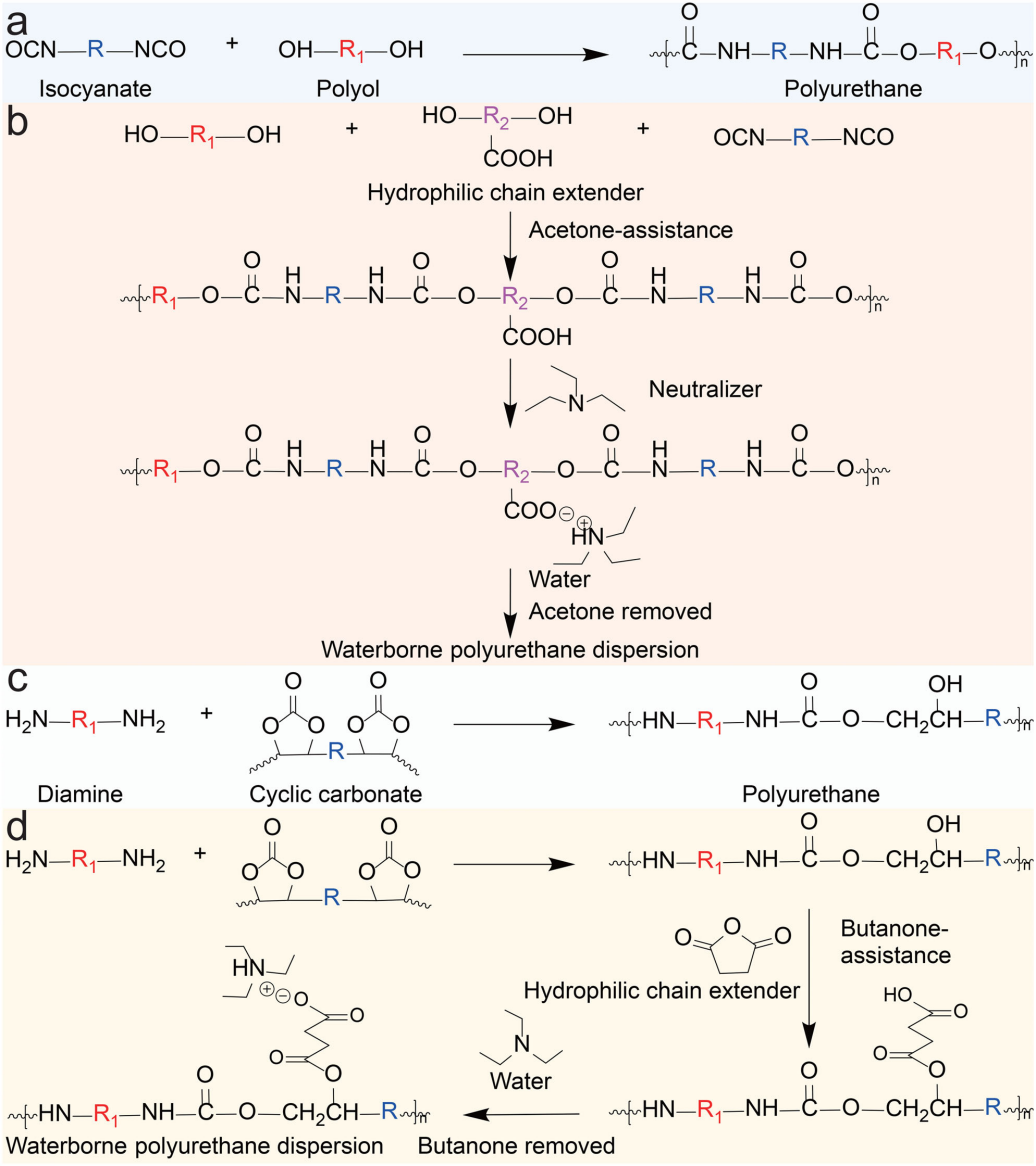
Polyurethane synthesis roadmap. (a) Typical synthesis route of solvent‐based polyurethane. (b) Typical synthesis route of waterborne polyurethane. (c) Typical synthesis route of non‐isocyanate solvent‐based polyurethane. (d) Typical synthesis route of non‐isocyanate waterborne polyurethane.

Based on the different combinations of the above components, various types of traditional bio‐based PU networks can be constructed (Figure 6) [85, 95]. For clarity, bio‑based PUs can be classified into four categories based on the extent of bio‑based component incorporation (Table 2). Type A, in which only the polyol is bio‑based, represents the most extensively studied class and the closest to commercial viability. Type B incorporates bio‑isocyanates but is limited by the current scarcity of commercially available bio‑isocyanate building blocks. Type C, the NIPU route, eliminates isocyanate toxicity entirely through carbonate‑amine chemistry but generally produces materials with lower mechanical performance. Type D represents fully bio‐based PU systems in which polyols, isocyanates, and chain extenders/internal emulsifiers are derived from renewable resources. Dynamic or recyclable network designs can be further incorporated into these systems to improve self‐healing, reprocessability, and end‐of‐life performance. The current research frontier is actively transitioning from Types A and B toward Type D systems. Oil‑based (solvent‑based) PU, a typical Type A system, is mainly synthesized by the direct polymerization of a bio‑based polyol with bio‑based or petroleum‑based isocyanates, resulting in a high‐molecular‐weight product with good film‑forming properties that is widely used in high‑performance coatings and elastomers. Based on this, a bio‑based internal emulsifier is introduced into WPU, and water dispersion is achieved via a phase conversion process, such as the acetone method or prepolymer dispersion method. The synthesis path usually involves introducing a hydrophilic chain extender, neutralizing, and dispersing in the water phase. Type C systems usually first react bio‐based raw materials, such as vegetable oil and epoxides, with carbon dioxide to form cyclic carbonates, and then polymerize with bio‐based polyamines to form a poly‐HU network containing hydroxyl groups [23]. This path not only avoids the use of toxic raw materials such as phosgene but also fixes carbon dioxide, reflecting the concept of green chemistry [96].

**TABLE 2:** Classification and comparison of various bio‐based polyurethane types.

Type	Bio‐polyol	Bio‐isocyanate	Bio‐based content (%)	Mechanical performance	Recyclability	Key limitation	Key ref∖erences
Conventional (Petroleum)	Petroleum (PPG, PTMG, PCL)	Petroleum (MDI, TDI, HDI, IPDI)	0	Tensile: 20–80 MPa Elongation: 200–800% Established, consistent	None (permanent covalent network)	Non‐renewable feedstock Non‐degradable waste No self‐healing	[97]
Type A	Bio‐based	Petroleum	20–60%	Tensile: 5–38 MPa (unfilled); ≤54 MPa (with Boron nitride nanosheets(BNNS) filler) Elongation: 50–500%	Limited (unless dynamic bonds incorporated)	Isocyanate toxicity Incomplete bio‐content Soft segment‐driven	[98]
Type B	Bio‐based	Bio‐based	40–80%	Tensile: 2–30 MPa (Often lower from limited isocyanate variety)	Limited (chain extender dependent)	Limited bio‐isocyanate commercial availability Higher cost (2–4×) Lower variety	[49]
Type C	Cyclic carbonates	Not applicable	30–100%	Tensile: 5–25 MPa Lower than conventional PU Improved adhesion	Moderate (inherent OH groups enable trans‐carbamoylation)	Lower MW and strength Slower curing kinetics Limited commercial adoption	[99]
Type D	Bio‐based	Bio‐based	70–95%	Tensile: 10–40+ MPa Self‐healing Reprocessability Programmed degradation	Excellent (dynamic covalent bonds: disulfide, imine, acylhydrazone, oxime‐carbamate)	Trade‐off between strength and healing kinetics Long‐term stability unknown	[67]

These research works have laid the foundation for the transition of bio‑based PU from laboratory to application. However, a critical gap in the bio‐based PU literature is the absence of systematic benchmarking against commercial petroleum‐derived PU. Table 3 addresses this by comparing castor oil‐based, soybean oil‐based, and lignin‐based PU systems with commercial PU across six performance dimensions. While bio‐based systems with nanofiller reinforcement can approach or even exceed commercial tensile strength (e.g., 54 MPa with BNNS at 10 wt%), most unfilled systems remain substantially below commercial benchmarks [106]. Crucially, bio‑based systems incorporating dynamic covalent bonds achieve self‑healing efficiencies of 80 to 100 percent and thermal reprocessability, capabilities fundamentally absent in conventional irreversible PU networks. However, cost, scalability, and batch‑to‑batch consistency remain the primary barriers to industrial translation. Furthermore, it should be noted that the PU networks constructed by the above traditional methods are structures with permanent covalent cross‑linking. This structure gives the material stable mechanical properties and solvent resistance, but it also imposes inherent limitations: the material cannot be reprocessed, repaired, or recycled, and it cannot respond to external stimuli [107, 108, 109]. This static characteristic has become a bottleneck for the material to move toward higher‑end and intelligent application fields. Therefore, overcoming the static constraints of its traditional network while retaining the advantages of bio‑based PU and endowing it with dynamic, reversible characteristics has become the focus of current research; this is also the core content of the subsequent chapters of this paper.

**TABLE 3:** Benchmark comparison: bio‐based polyurethane vs. commercial petroleum‐based polyurethane.

Property	Commercial petroleum polyurethane	Castor oil‐based polyurethane	Soybean oil‐based polyurethane	Application threshold	Key references
Tensile Strength (MPa)	Flexible: 20–50 Rigid: 40–80	5–38 (unfilled) Up to 54 (with BNNS)	3–25 (OH value dependent)	Coatings: ≥15 Biomedical: ≥5	[100]
Self‐Healing Efficiency (%)	0 (irreversible)	Up to 98%	60–85 (via transesterification)	≥80 for self‐healing label	[101]
Reprocessing / Recycling	Only mechanical downcycling	Multiple recyclability	Closed‑loop recyclability	≥3 cycles with ≥80% retention	[102]
Thermal Stability T_5_% (°C)	250–320	250–300	>200	Processing: ≥200 Service: ≥120	[103]
Relative Cost (vs. conventional)	1.0 (baseline)	1.2–2.5	0.8–1.5 (soybean scale benefit)	≤2.0 for commercial viability	[104]
Scalability (TRL^a^)	TRL 9 (fully commercial)	TRL 5–7 (pilot/commercial)	TRL 4–6 (lab/pilot)	≥TRL 5 for industrial translation	[105]

^a^ TRL = Technology Readiness Level (NASA scale: 1 = basic principles, 9 = proven operational system).

## 3 Structure Design and Performance Improvement Strategy of Bio‐Based Polyurethane

### 3.1 Chain Segment Design

The performance of PU is related to its chemical composition and the relative content of soft and hard segments [110]. The soft segments are generally composed of long‑chain polyols, whose flexible molecular conformation imparts low‑temperature elasticity and deformation properties to the material [88]. The hard segments are composed of a diisocyanate and a small‑molecule chain extender; with strong intermolecular forces, they provide strong support and thermal stability. As the core of the hard segments, the chain extender directly affects the interaction strength and aggregation morphology among them. The introduction of bio‑based polyols or chain extenders can reduce dependence on petroleum‑based resources while balancing the soft and hard segments [20, 70, 111, 112]. Appropriately increasing the soft segment content can expand the molecular chain's activity space and enhance ductility. Conversely, increasing the proportion of rigid chain extender strengthens the cohesion of the hard segments and improves the modulus and heat resistance. Therefore, precise control of the soft‑to‑hard segment ratio is the key to determining the mechanical properties of PU. Further introduction of bio‑based chain extenders with specific chemical functional groups can impart additional functions, such as antibacterial properties, anti‑adhesion, or stimulus‐responsive properties, while maintaining mechanical balance.

**FIGURE 7:**
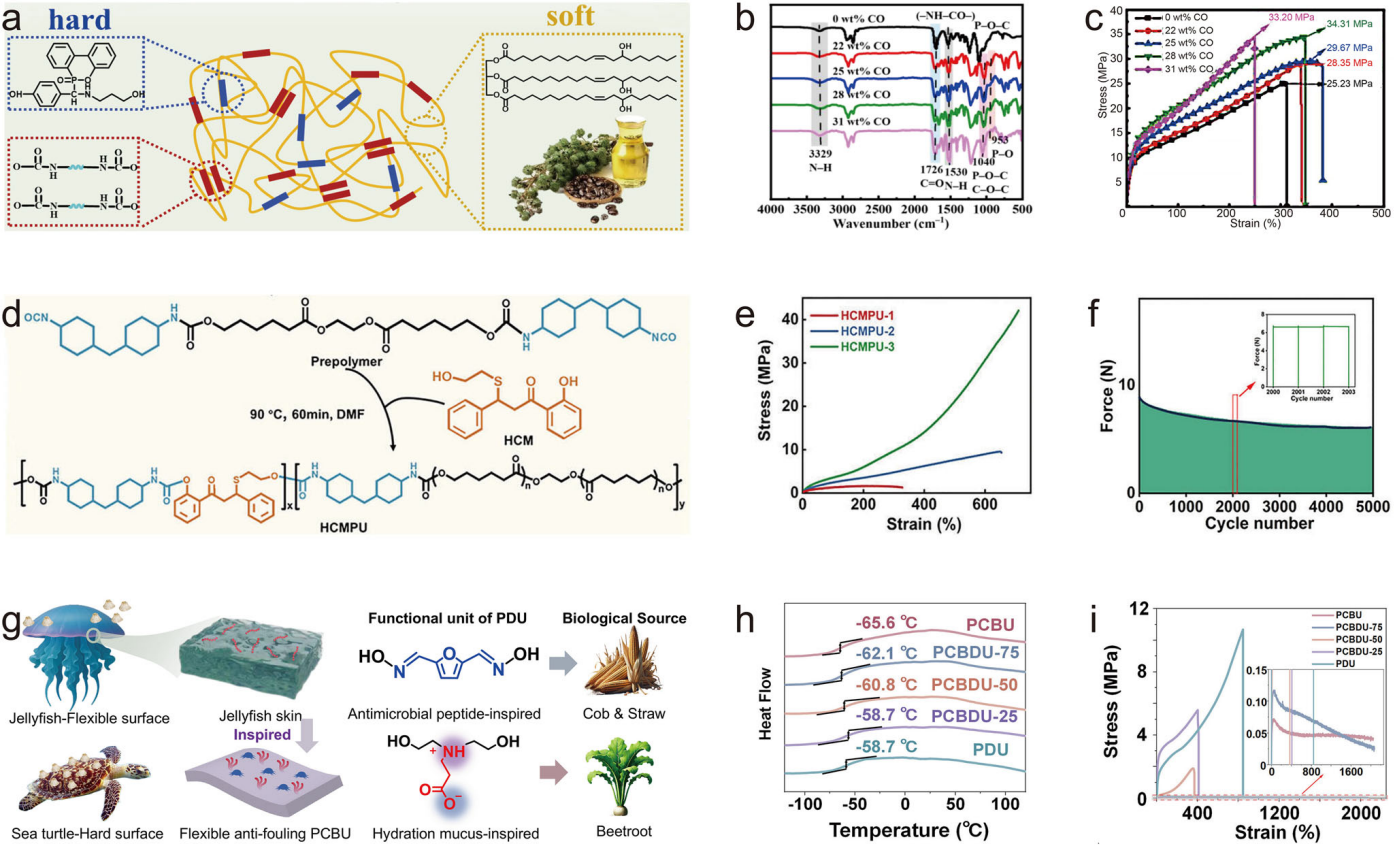
The effect of bio‐based soft segment and chain extender on polyurethane segment engineering. (a) Soft–hard segment structure of polyurethane synthesized from castor oil and DOPO derivatives. (b) Fourier transform infrared spectra of polyurethane films with varying castor oil contents. (c) Stress–strain curves of films with different castor oil contents. (a–c) Reproduced with permission [113]. Copyright 2026, Elsevier. (d) Synthetic route of HCMPU. (e) Stress–strain curves of HCMPU with different soft‑to‑hard segment ratios. (f) Cyclic tensile stability of HCMPU‑3 after 5000 cycles at 100% strain. (d–f) Reproduced with permission [114]. Copyright 2025, Wiley‐VCH. (g) Antifouling design of poly(oxime‑urethane) based on DFFD and DEAEA dual chain extenders. (h) DSC curves of poly(oxime‑urethane) with different DFFD contents. (i) Tensile strength and elongation at break of poly(oxime‑urethane) samples with various chain extenders. (g–i) Reproduced with permission [115]. Copyright 2026, Elsevier.

The proportions of soft and hard segments in PU directly affect its mechanical properties. Increasing the flexible soft segment content usually increases elongation at break but sacrifices some strength. The increase in rigid hard‐segment content enhances the modulus and mechanical strength. Therefore, establishing a rigid‑flexible coordination mechanism between the soft and hard segments is a key approach to simultaneously optimizing strength and toughness. Xiao et al. [113] introduced castor oil as a bio‑based flexible soft segment into the main chain of WPU and copolymerized it with a rigid chain extender derived from 9,10‑dihydro‑9‑oxa‑10‑phosphaphenanthrene‑10‑oxide (DOPO) to construct a molecular framework with synergistic soft and hard segments (Figure 7a).

The long fatty chain of castor oil enhances the mobility of its chain segments and the efficiency of stress dissipation. DOPO‑derived aromatic phosphaphenanthrene units provide thermally stable carbonization precursors and physical cross‐linking sites. Fourier transform infrared spectroscopy confirmed that castor oil and DOPO units were covalently connected to the PU main chain (Figure 7b). As the castor oil content increased, the intensity of the hydrogen bond absorption peak in the carbonyl region of carbamate intensified, indicating that the flexible soft segment facilitated the orderly accumulation of the hard segments and strengthened the hydrogen‑bond network. Stress‑strain curves quantified the contribution of the soft‑to‑hard segment ratio to the mechanical properties (Figure 7c). When the castor oil mass fraction reached 28%, the film tensile strength increased to 34.3 MPa, 35.9% higher than that of the sample without castor oil. The elongation at break increased synchronously to 347.9%, an increase of 11.7%. These results confirm that the synergism of bio‑based flexible soft segments and rigid hard segments can simultaneously improve strength and toughness.

In addition to the substitution of bio‑based soft segments, the type and structure of the chain extenders also affect the interactions among hard segments. The configuration of the small‑molecule chain extender and the polarity of its functional groups determine the cohesive energy strength and the segment composition of the hard‑segment region. Chain extenders derived from natural product skeletons can carry additional non‑covalent bond sites while introducing specific rigidity to finely regulate the aggregation behavior of hard segments. Fu et al. [114] synthesized a functional chain extender derived from the bio‑based chalcone analog 1‑(2‑hydroxyphenyl)‑3‑phenyl‑2‑propen‑1‑one (HCM) and combined it with polycaprolactone diol (PCL) soft segments and 4,4′‑methylenebis (cyclohexyl isocyanate) to prepare a new type of PU (HCMPU) (Figure 7d).

The *α,β*‑unsaturated ketones and ortho‑phenolic hydroxyl groups of 1‑(2‑hydroxyphenyl)‑3‑phenyl‑2‑propen‑1‑one molecules conferred HCM rigidity, thereby promoting π‑π stacking between hard segments during polymerization and strengthening the physical cross‐linking network. Stress‑strain curves showed the significant effect of the soft‑to‑hard segment ratio on the mechanical properties (Figure 7e): the tensile strength of the sample HCMPU‑3 reached 42.1 MPa, and the elongation at break increased to 710%. This result was attributed to the HCM‑promoted reduction in hard‑segment aggregation size and its uniform distribution, which optimized the stress transfer path [114]. Cyclic tensile tests verified the structural stability under long‑term dynamic loading (Figure 7f). After 5000 loading‑unloading cycles at 100% strain, the stress‑strain curve of HCMPU‑3 showed no obvious plastic deformation, because HCM‑enhanced intermolecular interactions served as reversible energy‑dissipating sites to maintain network integrity. This design not only achieved synergistic improvements in strength and high ductility but also endowed the material with fatigue resistance.

In addition to regulating mechanical properties, the functional design of polyol and chain extenders can endow PU with surface properties and environmental response behavior. Bio‐based molecules are often used to construct chain extenders that combine rigidity and responsiveness, thanks to their diverse functional groups. Hu et al. [115] used biomass‑derived furoyl dioxime (DFFD) as a rigid chain extender and incorporated it into the main chain of poly(oxime‑urethane) together with the hydrolyzable zwitterionic chain extender diethanolamine acetate (DEAEA) (Figure 7g). The rigid furan ring and oxime ester structure of DFFD strengthened the hard segments and endowed the material with intrinsic antibacterial activity. The side chain of DEAEA hydrolyzes in aqueous environments to form carboxybetaine zwitterions, which form a dense hydration layer that resists biological adhesion. Differential scanning calorimetry (DSC) showed that the glass transition temperature (*Tg*) of poly(oxime‑urethane) increased monotonically from −65.6°C to −58.7°C as the DFFD content rose from 0% to 100% (Figure 7h). The increase in the rigid furan ring content reduced the thermal mobility of the soft‑segment chains, thereby increasing *Tg*. Tensile tests quantified the effect of the ratio of the two chain extenders on the mechanical properties (Figure 7i). The tensile strength of the pure DFFD system PDU was 10.7 MPa, and the elongation at break was 840%. As the DEAEA ratio increased, the tensile strength decreased gradually, while the elongation at break synchronously increased to 2069.7%. The rigid DFFD skeleton constructed a load‑bearing matrix, while the dynamic interaction of the side chains of DEAEA provided the molecular chain with a large deformation space. This chain extender combination achieved ultra‑wide flexibility while ensuring basic strength, and endowed the material with long‑term resistance to biological adhesion.

### 3.2 Aggregation Structure

The mechanical properties of bio‑based PU are closely related to its multi‑scale aggregated structure. The microphase‑separated structure determines the spatial distribution and interfacial interaction strengths of the soft and hard segments [116, 117, 118]. The topological structure of the cross‐linked network further regulates the motion of chain segments and the stress‑transfer path [29, 119]. The chemical structures and rich polar groups of bio‑based monomers provide a chemical platform for regulating the scale of phase separation and network dynamics. Rigid bio‑based segments can induce the formation of hard‑phase crystalline microdomains, thereby increasing the elastic modulus. In contrast, flexible bio‑based chain segments reduce the interfacial energy at the phase boundary, thereby promoting the uniform dispersion of the hard‑phase microdomains. Bio‑based compounds containing polyhydroxyl or phenolic hydroxyl groups can also form high‑density hydrogen‑bond cross‐linking networks [120, 121, 122]. These dynamic physical/chemical cross‐linking points dissipate a large amount of energy through reversible dissociation and recombination under the action of external forces, thereby enhancing the mechanical properties of bio‑based PUs.

**FIGURE 8:**
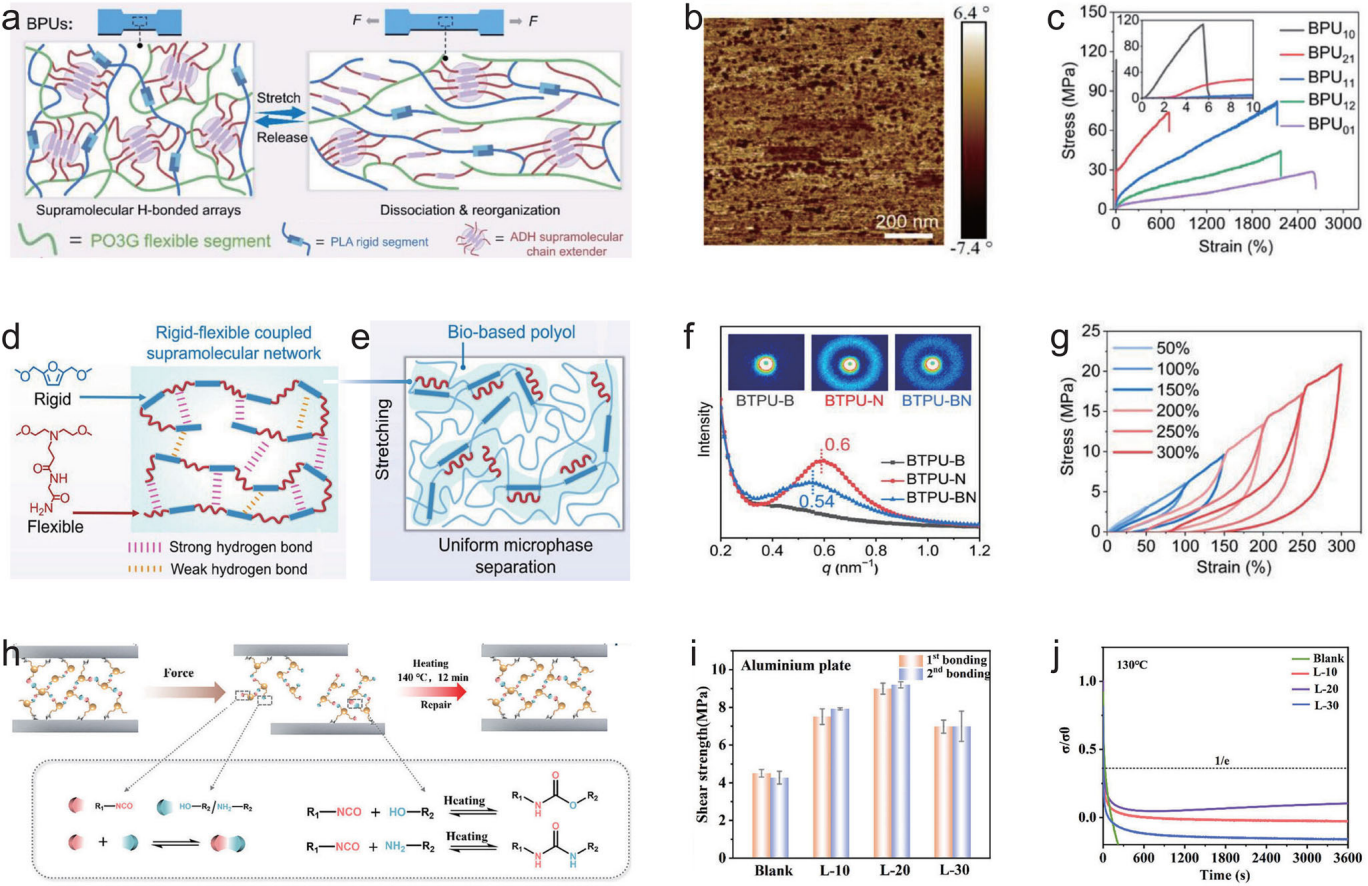
Key role of bio‐based components in regulating microphase separation and cross‐linking networks of polyurethane. (a) Design principle of the BPU network with rigid PLA and flexible PO3G hybrid soft segment. (b) Atomic force microscopy phase image of nanoscale uniform microphase‐separated morphology in BPU_11_. (c) Stress–strain curves of BPU showing simultaneous improvement in tensile strength and elongation at break via optimized soft segment ratio. (a–c) Reproduced with permission [123]. Copyright 2025, Wiley‐VCH. (d) Schematic design of rigid–flexible coupled supramolecular networks. (e) Microphase separation of the polymer. (f) 1D small‑angle X‑ray scattering profile and 2D scattering pattern confirming a typical microphase‐separated structure in BTPU. (g) Cyclic loading–unloading tensile curves of BTPU at various incremental strains. (d–g) Reproduced with permission [124]. Copyright 2025, Wiley‐VCH. (h) Schematic of the dynamic dual‐cross‐linking network reuse mechanism in lignin‐based waterborne polyurethane. (i) Bonding strength retention of lignin‑based waterborne polyurethane adhesive on aluminum substrates over ten hot‐pressing cycles. (j) Stress relaxation curves of lignin‑based waterborne polyurethane with different lignin contents at 130°C. (h–j) Reproduced with permission [125]. Copyright 2024, Wiley‐VCH.

The microphase‑separated structure plays a key role in balancing strength and toughness in PU elastomers [37]. The micro‑regions of the hard segments act as physical cross‐linking points to enhance the modulus, while the continuous phase of the soft segment confers flexibility. However, excessive aggregation of the hard phase may lead to stress concentration and brittle fracture. Optimizing the compatibility between soft and hard segments and the size distribution of the hard phase is key to obtaining high‑strength, high‑toughness elastomers. Zheng et al. [123] proposed the preparation of bio‑based PU elastomers (BPUs) based on rigid‑flexible mixed soft segments (Figure 8a). They copolymerized bio‑based polylactic acid polyol (PLA) and bio‑based poly(1,3‑propanediol) (PO3G) as hybrid soft segments. The rigid PLA segments formed crystalline microdomains, thereby maintaining mechanical strength. In contrast, the flexible PO3G segment reduced the phase interface energy of both the soft and hard segments through its conformational entropy‑buffering effect. This thermodynamic incompatibility, driven by differences in the characteristics of the bio‑based components, can promote the orderly accumulation of hard‑segment micro‑regions. Atomic force microscopy phase images confirmed a uniform microphase‑separated morphology (Figure 8b): the light and dark nanoscale regions corresponded to the hard and soft segment, respectively, with a uniform spatial distribution at the nanoscale. Stress–strain curves further indicated that the above structure was the direct cause of the mechanical enhancement (Figure 8c). As the molar ratio of bio‑based PLA to PO3G approached an optimal value, the degree of equilibrium microphase separation was optimized, and the resulting sample exhibited a tensile strength of 82.29 MPa and an elongation at break of 2128%. These results show that the bio‑based hybrid soft segments simultaneously endow PU with high strength and toughness by adjusting the phase‑separation morphology.

In addition to regulating the soft‑segment components, the molecular configuration of the chain extender in the hard segments also decisively influences the uniformity of microphase separation. Rigid chain extenders promote the orderly accumulation of hard segments, but can lead to excessive aggregation of hard phases. Flexible chain extenders can enhance the mobility of chain segments, but may weaken the cohesive energy within the hard‑phase microdomains. The synergistic coupling of rigid and flexible chain extenders can refine the hard‑phase structure and achieve a gradient distribution of hydrogen bonds. Inspired by the hierarchical structure of articular cartilage, Li et al. [124] proposed a rigid‑flexible coupling supramolecular cross‐linking strategy (Figure 8d,e). They used bio‑based PO3G as a soft segment to construct an amorphous continuous phase, and introduced a rigid furan‑ring chain extender and a flexible amide side‑chain extender to build the hard segments. The rigid furan ring inhibited the excessive entanglement and disordered aggregation of the hard‑segment backbone. At the same time, the flexible amide side chain enhanced cohesive energy within the hard‑segment microdomains through high‑density hydrogen bonding. This bio‑based hybrid hard‑segment design induced the formation of a microphase‑separated structure with uniform size and a continuous spatial distribution, as confirmed by synchrotron radiation small‑angle X‑ray scattering (Figure 8f). The scattering spectra exhibited clear isotropic scattering rings and broad shoulder peaks. Correlation function analysis showed that the hard‑phase size was only 3.59 nm, and the phase spacing was significantly reduced, which was derived from the optimization of the spatial distribution of the hydrogen‑bond network by the bio‑based components. Incremental strain cyclic tensile curves revealed the energy‑dissipation advantage of this structure (Figure 8g): the hysteresis loop area gradually increased with increasing strain, because weak hydrogen bonds were preferentially dissociated at low stress to dissipate the initial energy, while strong hydrogen bonds maintained the overall structural integrity at high stress. Therefore, the synergistic coupling of bio‑based soft and hard segments can significantly enhance mechanical toughness by regulating the phase‑separation morphology.

The cross‐linked network not only provides mechanical support for PU, but also endows the material with reprocessability and long‑term durability [126]. Natural polyphenol compounds are rich in phenolic hydroxyl and carboxyl functional groups, which can simultaneously construct covalent and physical double cross‐linking networks. Li et al. [125] integrated low‑molecular‑weight lignin with carbon dioxide‑based polyol to prepare dynamic cross‐linked WPU adhesives (Figure 8h). They partially replaced petroleum‑based polyol with lignin, thereby introducing rigid aromatic skeletons. The phenolic hydroxyl groups of lignin reacted with isocyanate to form thermally reversible phenolic‑carbamate covalent bonds. In contrast, the carboxyl and polar groups of lignin formed a high‑density hydrogen‑bonded physical cross‐linking network, giving the adhesive repeated bonding ability. Cyclic bonding tests (Figure 8i) provided direct evidence for this: the lap shear strength of the aluminum plate substrate remained above 9.2 MPa after 10 hot‐pressing cycles, and the bonding strength retention rate exceeded 85% of the initial value. This performance was attributed to the reversible bond‑breaking and recombination of dynamic phenolic‑carbamate bonds at high temperature. Stress‑relaxation curves further verified the dynamic exchange characteristics of the cross‐linking network (Figure 8j): the lignin‑containing sample fully relaxed to zero at 150°C, whereas the lignin‑free blank group exhibited significantly accelerated relaxation, and no dynamic exchange plateau was observed. Bio‑based lignin simultaneously enhanced interface robustness and closed‑loop recycling efficiency by forming dynamic cross‐linking centers.

### 3.3 Construction and Collaboration of Dynamic Molecular Networks

#### 3.3.1 Single Dynamic Bond

The dynamic network design of bio‐based PU, featuring covalent and non‑covalent bonds, has made remarkable progress in recent years. Researchers have endowed materials with self‑healing, reprocessing, and shape‑memory functions by embedding a single dynamic bond (e.g., disulfide, imine, acylhydrazone, or oxime‑carbamate) into the cross‐linking network [127, 128, 129]. The reversible breaking and recombination of dynamic bonds usually depend on the electronic structure and steric hindrance of the bonds. The long aliphatic chain, aromatic ring, or multi‑hydroxyl structure of bio‑based raw materials provides a regulatory platform for dynamic networks [72]. Biomass‑derived components can also actively regulate the dynamic bond‑exchange efficiency by affecting chain mobility, hydrogen‑bond density, and network rigidity.

**FIGURE 9:**
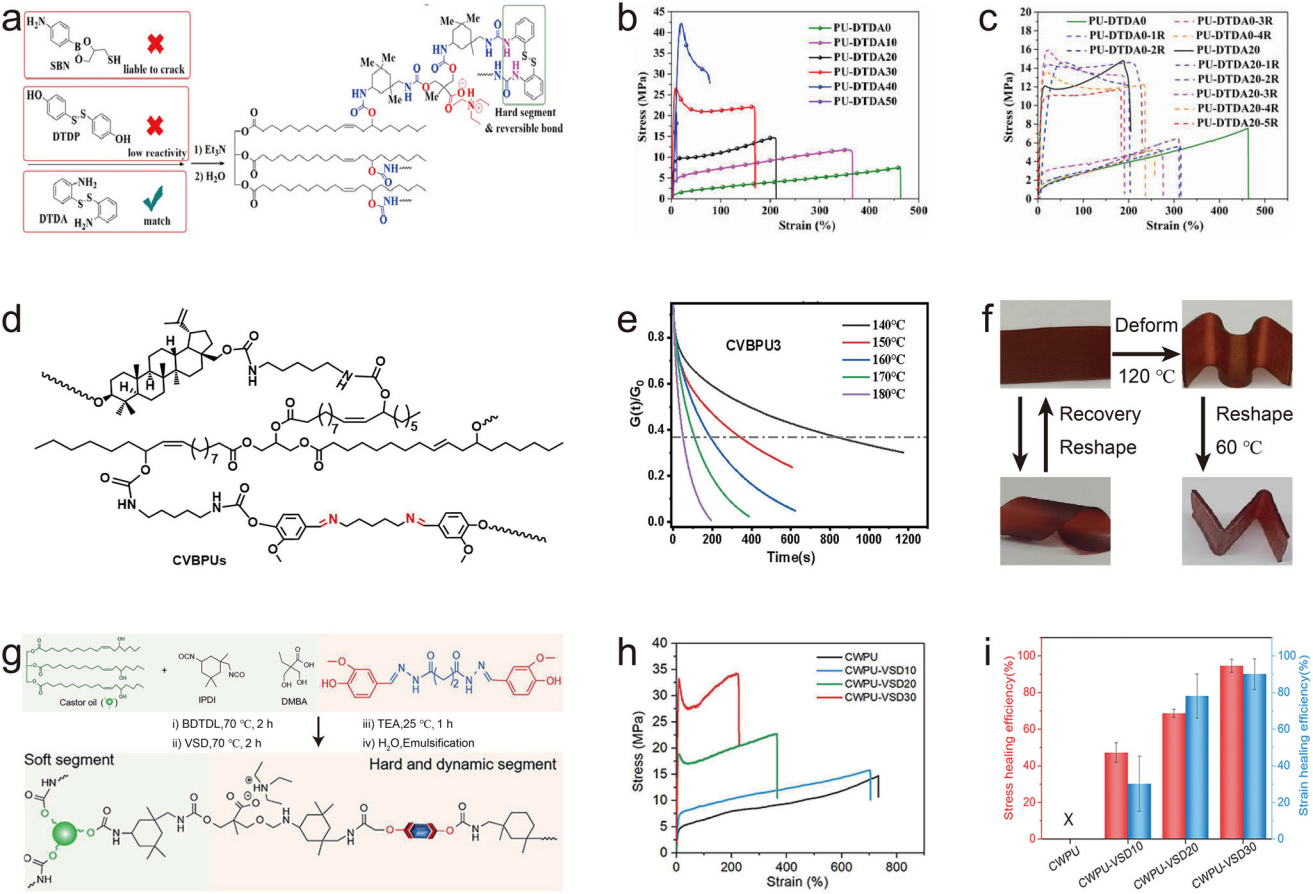
The synthesis route, mechanical regulation effect, and functional response behavior of a single dynamic bond in bio‐based polyurethane. (a) Schematic diagram of the synthesis route for DTDA and castor oil‑based waterborne polyurethane dispersion. (b) Stress–strain curves of polyurethane films with different DTDA content. (c) Stress–strain curve of polyurethane film after multiple reprocessing. (a–c) Reproduced with permission [131]. Copyright 2020, Wiley‐VCH. (d) Molecular skeleton of fully bio‑based polyurethane (CVBPU). (e) Stress relaxation curves of CVBPU3 at different temperatures. (f) Editable shape memory process of CVBPU3. (d–f) Reproduced with permission [132]. Copyright 2024, Elsevier. (g) Synthesis of VSD and castor oil‑based waterborne polyurethane dispersions. (h) Stress–strain curves of polyurethane films with different VSD contents. (i) Self‑healing efficiency of tensile strength and elongation of polyurethane films with different VSD content. (g–i) Reproduced with permission [133]. Copyright 2025, The Royal Society of Chemistry.

A disulfide bond is a classical dynamic covalent bond that responds to heat and light. The exchange mechanism usually follows a free radical or ionic reversible cleavage‑recombination pathway [130]. The disulfide bond readily cooperates with the flexible vegetable oil segment to form an adaptive network in the bio‑based PU system. Aromatic disulfides can also contribute an additional rigid benzene ring to compensate for the strength loss caused by the introduction of dynamic bonds. Zhang et al. [131] introduced aromatic diamines (DTDA) containing disulfide bonds into castor oil‑based WPU networks (Figure 9a). In this work, DTDA partially replaced castor oil as the hard‑segment chain extender. As the DTDA content increased, tensile strength rose from 7.2 to 38.42 MPa (Figure 9b). The dynamic disulfide bond and the carbamate hydrogen bond together give the network self‑healing ability. After four hot‑pressing reprocessing cycles, the tensile‑strength recovery rate remained 100% (Figure 9c). The long, flexible chain of castor oil maintains the mobility of its segments, ensuring dynamic bond recombination. The system successfully reconciles the traditional contradiction between high strength and high repair efficiency.

The imine bond is formed by reversible condensation of an amine and an aldehyde group. It is stable under anhydrous conditions and reversibly dissociates in hot, humid, or acidic environments. The exchange reaction of the imine bond usually does not require an additional catalyst. Bio‑based aromatic aldehydes (such as vanillin) provide a natural platform for the construction of whole‑biomass imine networks. Xu et al. [132] synthesized a vanillin‐based diol chain extender containing an imine bond using vanillin and bio‑based 1,5‐pentanediamine. The CVBPU molecular skeleton was formed by copolymerization of the vanillin‐based diol chain extender containing an imine bond with betulin, castor oil, and pentane diisocyanate (Figure 9d). The large steric hindrance skeleton and the imine bond of betulin synergistically regulate the network's rigidity and dynamics. The stress relaxation time at high temperature decreased from 840 s to 48 s (Figure 9e). Dual‑shape editing behavior driven by glass transition and topological rearrangement was demonstrated (Figure 9f). The imine bond is rapidly hydrolyzed under acidic conditions, completely degrading the material. This work demonstrates the potential for integrating whole biomass feedstocks into a dynamic network.

The acylhydrazone bond is formed by the condensation of hydrazide and aldehyde. Its water stability is better than that of the imine bond and can provide additional hydrogen bond sites [31]. The acylhydrazone bond can simultaneously enhance the mechanical strength and self‑healing efficiency in bio‑based PU. Zhou et al. [133] used lignin‑derived vanillin and succinylhydrazine to synthesize rigid diols (VSDs) containing acylhydrazone bonds. VSD was embedded in a castor oil‑based WPU network (Figure 9g). The rigid benzene ring and multiple hydrogen bonds enhanced the tensile strength from 14.5 to 33.9 MPa. Stress–strain curves revealed precise regulation of mechanical properties by VSD content (Figure 9h). The tensile strength recovery rate after repair was 93.6% (Figure 9i). The coating can be quickly dissociated in an acidic alcohol solution to achieve closed‑loop recovery of the paper substrate. In this work, bio‑based hard segments are actively involved in dynamic bond formation and in regulating network structure.

**FIGURE 10:**
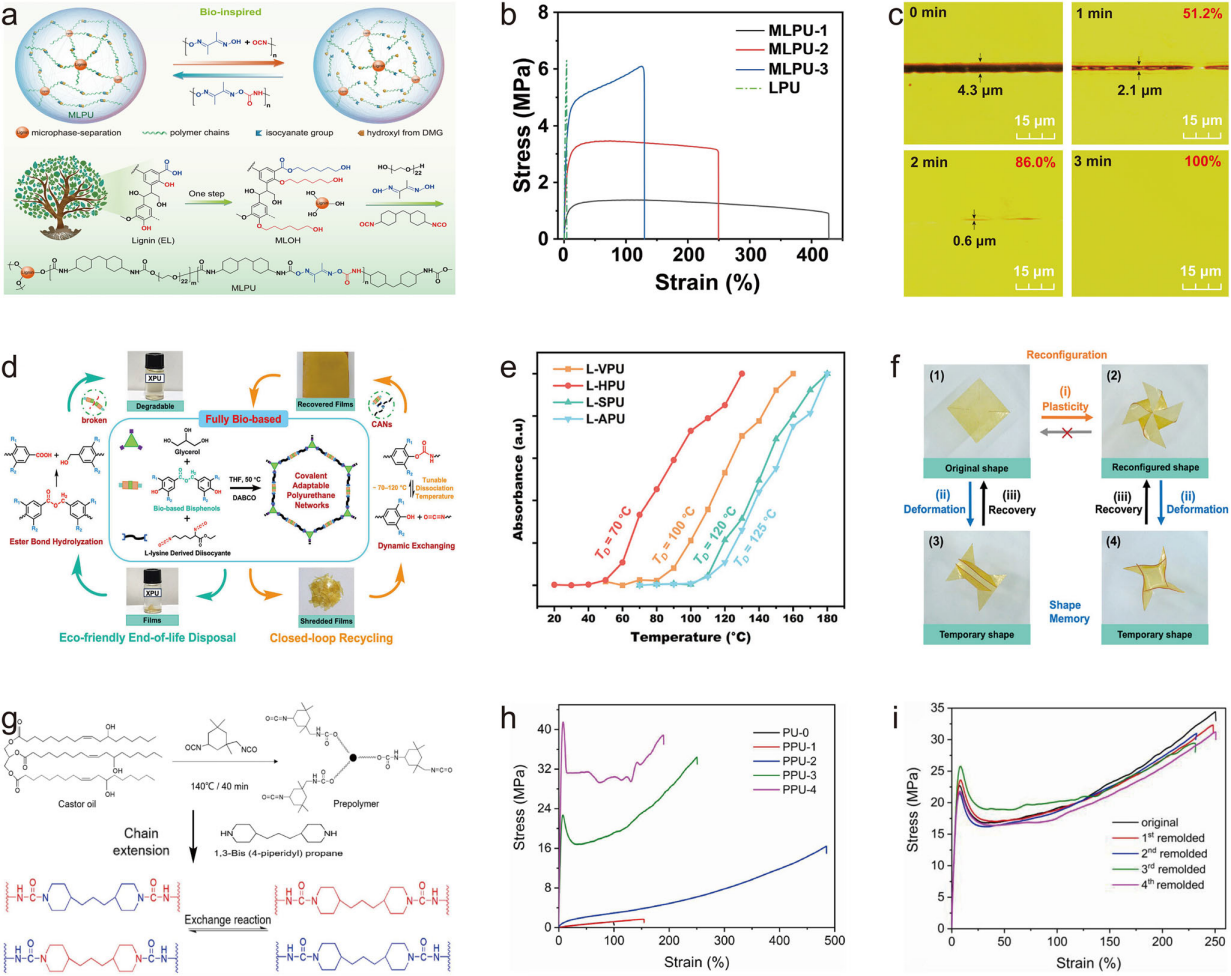
The synthesis route, mechanical regulation effect, and functional response behavior of a single dynamic bond in bio‐based polyurethane. (a) Synthesis of modified lignin polyol and schematic of dynamic oxime‑carbamate mechanism. (b) Stress–strain curves of polyurethane films with different modified lignin polyol contents. (c) Optical microscopy images of MLPU‑2 films after different repair times at 80°C. (a–c) Reproduced with permission [134]. Copyright 2023, Elsevier. (d) Bio‑based bisphenol dynamic polyurethane network and its closed‑loop recovery pathway. (e) Temperature‑dependent free isocyanate peak intensity of different bisphenol linear model compounds. (f) Shape memory and topological reconstruction process of polyurethane film. (d–f) Reproduced with permission [135]. Copyright 2025, Wiley‐VCH. (g) Schematic of the synthesis route of castor oil‑based polyurethane urea (PPU). (h) Stress–strain curves of PPU with different PIP contents. (i) Stress–strain curves of PPU‑3 before and after multiple reprocessing. (g–i) Reproduced with permission [136]. Copyright 2022, Elsevier.

The thermally reversible addition of oxime and isocyanate forms the oxime‐carbamate bond. The dissociation temperature is moderate, and the oxime structure can be readily tuned to adjust the dynamic exchange behavior. This bond can synergize with the microphase‐separated structure to enhance both the mechanical properties and the bond exchange rate of bio‐based PU networks. Zheng et al. [134] designed a lignin‑enhanced dynamic PU network inspired by the two‑phase structure of spider silk (Figure 10a). Enzymatic lignin was converted into a highly reactive aliphatic hydroxyl polyol by alkylation. Increasing modified lignin polyol content increased toughness by 41 times compared with the unmodified system (Figure 10b). Scratches healed completely within 3 min at 80°C without pressure (Figure 10c). The microphase separation structure increased the phase interface area and promotes energy dissipation. The bonding strength of the material as a hot‑melt adhesive to steel reached up to 10.5 MPa. Bio‑based lignin actively defines network viscoelasticity through nano‑scale phase separation.

The phenol–carbamate bond is formed by the reaction of the phenolic hydroxyl group and isocyanate. The dissociation temperature can be adjusted in a wide range by the electronic effect of benzene ring substituents. This bond can simultaneously endow reprocessability and degradability in bio‑based PU. Wang et al. [135] synthesized bio‑based bisphenol monomers containing ester bonds via quaternary coupling. The dynamic network was constructed from bisphenol, lysine diisocyanate, and glycerol, with a closed‑loop recovery pathway (Figure 10d). Lignin‑derived aromatic aldehydes provided aromatic skeletons with adjustable electronic properties. The bisphenol substituent greatly adjusted the bond dissociation temperature from about 70 to 120°C (Figure 10e). The rigid aromatic ring and multiple hydrogen bonds increased the tensile strength to 33 MPa. The network exhibited both temporary shape‑memory and permanent topology‑reconstruction capabilities (Figure 10f). The incorporation of ester bonds enables rapid hydrolysis and degradation of the material under alkaline conditions. The bio‑based aromatic structure actively defines a dynamic response window through electronic and spatial effects.

The piperidine‑urea bond is a new type of dynamic urea bond formed by the addition of cyclic secondary amines to isocyanates. Its resonance energy is between the ordinary urea bond and the sterically hindered urea bond. This bond can cooperate with the rigid ring structure in bio‑based PU to achieve a wide range of adjustment of mechanical properties. Xie et al. [136] constructed a dynamic PU urea network using castor oil, isophorone diisocyanate, and 1,3‑bis(4‑piperidinyl) propane (PIP) as raw materials (Figure 10g). PIP containing a double six‑membered piperidine ring acts as a rigid chain extender and a reversible cross‐linking site. The tensile strength increased from 1.2 to 41.5 MPa, and the Young's modulus synchronously increased from less than 2 MPa to about 1000 MPa (Figure 10h). The mechanical properties of the network were almost fully restored after multiple hot‑pressing cycles (Figure 10i). The system can also be chemically recovered by additional PIP deconstruction and refeeding. The castor oil soft segment and reversible piperidine‑urea cross‐linking synergistically constructed a network for multi‑path recovery.

Collectively, the above examples demonstrate the rich diversity of dynamic covalent bond chemistries available for bio‑based PU design. A systematic comparison of the six representative dynamic bond types discussed in this section reveals distinct performance profiles (Table 4). Disulfide bonds represent the most mature dynamic chemistry, offering healing temperatures of 60 to 100°C and self‑healing efficiencies approaching 100% tensile strength recovery, but typically require elevated temperatures or UV exposure for bond exchange. Imine bonds enable lower‑temperature exchange without catalysts, benefiting from the abundance of aldehyde groups in modified bio‑based feedstocks such as vanillin and dialdehyde cellulose; however, their susceptibility to hydrolysis in humid or acidic environments, while advantageous for triggered degradation, is problematic for applications requiring long‑term water resistance. Acylhydrazone bonds provide superior hydrolytic stability compared to imine bonds and contribute additional hydrogen bond sites for mechanical reinforcement, but their exchange kinetics are generally slower. Oxime‑carbamate bonds offer the widest tunable dissociation temperature range, enabling application‑specific thermal programming, though the limited commercial availability of diverse oxime precursors currently restricts their widespread adoption. Phenol‑carbamate bonds offer high‑temperature exchange with good mechanical strength retention, though the limited number of reprocessing cycles typically reported constrains their applicability for multiple‑life‐cycle materials.

**TABLE 4:** Critical comparison of dynamic covalent bond chemistries in polyurethane.

Dynamic bond	Exchange Ea (kJ/mol)	Activation temp (°C)	Self‐healing efficiency (%)	Environmental sensitivity	Scalability	Key references
Disulfide	51–94	60–100 (thermal) UV (room temp)	Tensile: 80–96%; stress/strain recovery (70 °C, 2 h)	• Moderate: thiol–disulfide exchange; • UV‑active	• Most mature; • High scalability	[137]
Imine	38–102 (cooperation with disulfide can further reduce Ea)	25–80 (room temp to moderate heat)	Tensile: 80–97% Elongation: 85–92%	• High: Rapid hydrolysis under acidic (pH <5); • Moisture‐sensitive	• Well‐established; • Scalability: • Med‐High	[138]
Acylhydrazone	∼48	50–90 ((thermal activation at 125 °C or acetic acid triggering))	Tensile: 75–90% Self‐healing in water possible	• Moderate: Acid‐catalyzed exchange; • Better water stability than imine	• Proof‐of‐concept; • Scalability: Medium	[139]
Oxime‐Carbamate	∼124	30–120 (highly tunable)	Tensile: 97% (synergy with H‑bonds); complete healing	• Low: Excellent hydrolytic stability; • Dissociation	• Emerging chemistry; • Scalability: low‐Med	[140]
Phenol‐Carbamate	∼92.7	50–100	Almost complete healing (80 °C, 2 h)	• Low: Good humid stability; • Slower exchange vs. disulfide/imine	• Growing interest; • Scalability: Medium	[141]
Piperidine‐Urea (cyclic sec. amine + isocyanate)	44–150	80–140 (thermal;catalyst‐free)	Tensile: 85–100% Full property restoration after reprocessing	• Low: Good hydrolytic stability;	• Rapidly growing; • Scalability: Med‐High	[136]

Despite these advances, several critical challenges remain. The fundamental trade‑off between high bond stability required for structural applications and low activation energy needed for facile reprocessing remains unresolved for most single dynamic bond systems. Furthermore, limited understanding of the synergistic or antagonistic interactions between different dynamic bonds within the same network hinders the rational design of multi‐dynamic‐bond systems. Finally, the long‐term stability of dynamic bonds under realistic service conditions, including exposure to moisture, oxygen, and cyclic mechanical loading, requires systematic investigation.

#### 3.3.2 Multiple Dynamic Bonds

The introduction of a single dynamic bond into bio‑based PU materials can confer the ability to rearrange network topology. However, a single dynamic bond system often faces the challenge of balancing mechanical strength and dynamic exchange efficiency. The synergism of multiple dynamic bonds offers a new strategy to address this challenge, where two or more dynamic cross‐linking points of different bonding types coexist within the same network. One dynamic bond can be preferentially broken to dissipate energy, while the other maintains the network's overall integrity and provides reaction sites for structural rearrangement. Moreover, the synergism can also reduce the activation energy of the network's overall relaxation. The multiple dynamic bond network strengthens and toughens through the sacrificial bond mechanism under stress. Bio‑based compounds provide abundant modifiable sites for the construction of multiple dynamic networks [9, 56]. The long aliphatic chain and rigid aromatic ring structure of bio‑based monomers provide flexibility and strength. The integration of multiple dynamic bonds and bio‑based skeletons achieves the unity of high strength and rapid self‑healing [128]. Such multiple dynamic networks enable the elastomer to exhibit self‑healing, shape memory, and reprocessing capabilities simultaneously. Therefore, the multiple dynamic bond design of bio‑based PU expands the application boundary of materials.

**FIGURE 11:**
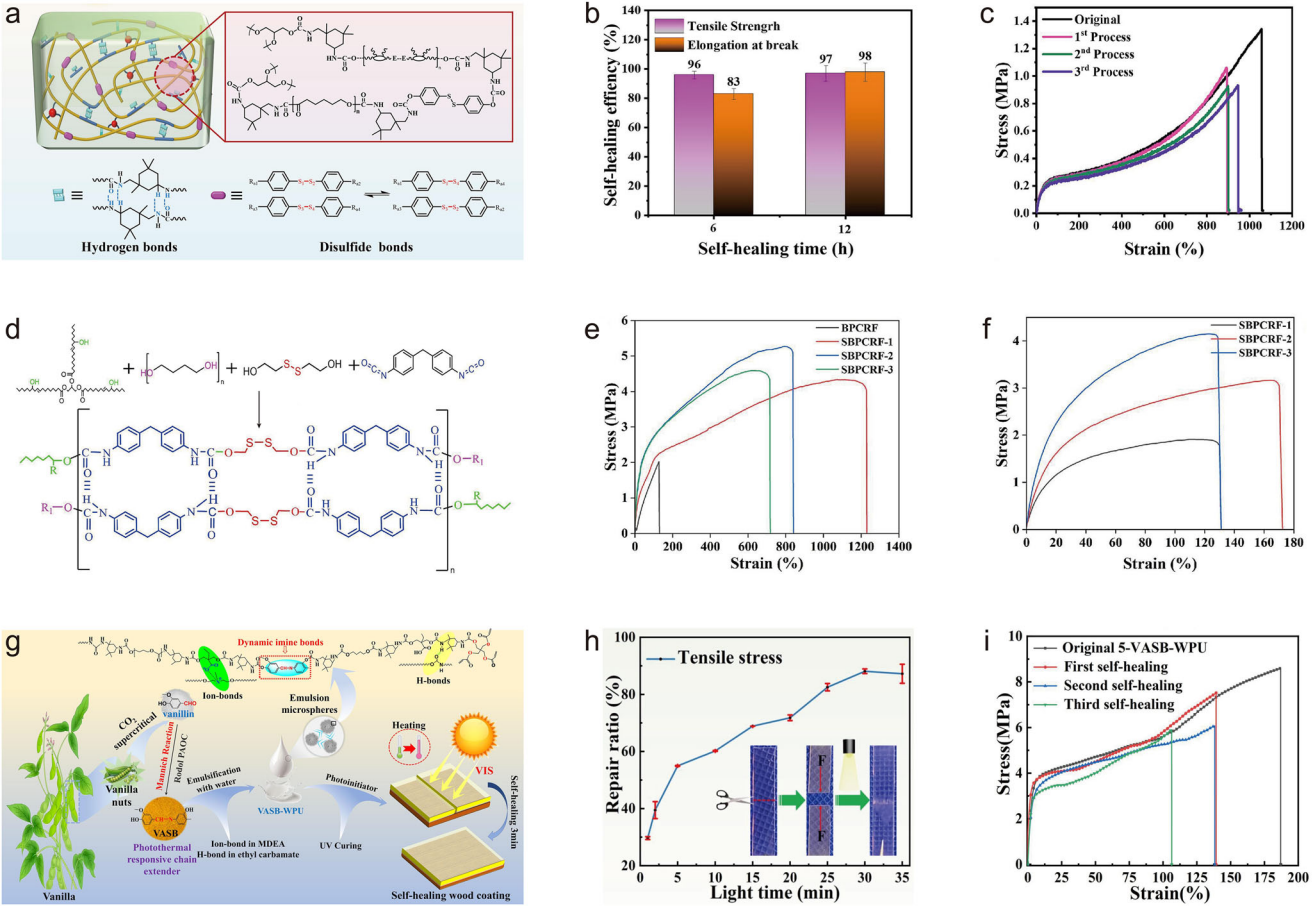
Mechanism and core data of bio‑based multiple dynamic bond synergy from structural design to functional expression. (a) Dynamic network structure of hydrogen and disulfide bonds in bio‑based polyurethane elastomers. (b) Histogram of self‑healing efficiency versus healing time at 60°C. (c) Stress–strain curve comparison of elastomer after three solution‑recovery cycles. (a–c) Reproduced with permission [142]. Copyright 2025, Elsevier. (d) Schematic synthesis of disulfide bond–hydrogen bond synergistic network in self‑healing bio‑based polyurethane coating. (e) Original stress–strain curves of polyurethane elastomers with different formulations. (f) Histogram of self‑healing efficiency at 25°C for elastomers with different DTD content. (d–f) Reproduced with permission [143]. Copyright 2025, Elsevier. (g) Preparation strategy and multiple dynamic bond network of vanillin‑based waterborne polyurethane coating. (h) Self‑healing efficiency as a function of visible‑light irradiation time after fracture. (i) Tensile stress of the elastomer after multiple reprocessing. (g–i) Reproduced with permission [144]. Copyright 2023, Elsevier.

A typical example of double dynamic bond synergism is the coupling of disulfide and hydrogen bonds in bio‑based PU networks. The disulfide bond can be covalently introduced into the molecular main chain by a disulfide‑containing chain extender. Hydrogen bonds can form between carbamates and polar groups, such as ester groups. Reversible covalent cross‐linking via disulfide bonds can rearrange network topology at high temperatures [29]. Hydrogen bonds provide rapidly reconstructed physical cross‐linking sites to maintain the overall structural stability. The synergistic effect of the two endows the material with fatigue resistance and reprocessability. Wang et al. [142] prepared bio‑based PU elastomers using fully bio‑based polyester diol and PCL as soft segments. The dynamic network structure of the elastomer was composed of disulfide and hydrogen bonds (Figure 11a). The ester group on the PCL segment formed a dense hydrogen bond array with the polar group of the hard segments, and the disulfide bond was introduced into the cross‐linking system via the chain extender dihydroxydiphenyl disulfide. The elongation‑at‑break repair efficiency reached 83% after 6 h of healing at 60°C (Figure 11b). After three solution‑recovery cycles, the tensile strength decreased only slightly (Figure 11c). Furthermore, the bio‑derived long‑chain fully bio‑based polyester diol reduced the glass transition temperature to ‐35°C, which enhanced the mobility of the molecular chains at room temperature. The improvement in mechanical properties is due to hydrogen and disulfide bonds dissipating external energy by breaking in sequence under stress, thereby reducing stress concentration. This work shows that bio‑based soft segments can serve to improve the flexibility of PU molecular chains and dynamic bond cross‐linking density, thereby synergistically optimizing mechanical and self‑healing properties.

The synergistic effect of disulfide bonds and hydrogen bonds can further enhance mechanical strength and significantly improve self‑healing efficiency. Wang et al. [143] prepared self‑healing bio‑based PU with castor oil and polytetrahydrofuran as mixed soft segments. The dynamic covalent network was formed by disulfide and hydrogen bonds (Figure 11d). The introduction of bis(2‑hydroxyethyl) disulfide (DTD) reduced the glass transition temperature of the elastomer to −40.88°C, which significantly improved the mobility of the molecular chain segments. The self‑healing PU exhibited a tensile strength of 5.271 MPa and an elongation at break of 1227.87% (Figure 11e). The self‑healing efficiency at 25°C varied with DTD content, reaching a maximum of 92.2% (Figure 11f). The disulfide bond undergoes spontaneous homolysis and recombination reaction after damage. Density functional theory calculations confirm that the Gibbs free energy of the recombination process is negative. The hydrogen bond restores interfacial bonding force by migrating and reassociating at the fracture surface. The castor oil matrix achieves a balance between high strength and rapid self‑healing by adjusting cross‐link density and chain flexibility.

The dual dynamic bond synergism can be further extended to the multiple coupling of dynamic covalent bonds and supramolecular ionic bonds. Vanillin‑derived aromatic Schiff bases (VASB) can simultaneously provide dynamic imine bonds and photothermal conversion capabilities. Chen et al. [144] introduced VASB as a chain extender into the main chain of waterborne PU to prepare a photothermal self‑healing coating. The preparation strategy and multiple dynamic bond network of the VASB‑WPU coating are presented in Figure 11g. The conjugated aromatic ring of vanillin give the material a strong visible light absorption capacity, and the photothermal conversion raised the film's surface temperature to 121.9°C within 220 s. Under visible‑light irradiation, the self‑healing efficiency reached 92.43% within 30 min (Figure 11h).

After three reprocessing cycles, the fracture strain remained 70% higher than that of the original film (Figure 11i). The imine bond dissociated and reorganized under photothermal driving, enabling the reconstruction of network topology. Hydrogen bonds and ionic bonds reassociated at the fracture surface, restoring the interfacial bonding force. The coating exhibited a tensile strength of 10.59 MPa and a toughness of 16.56 MJ m^−^^3^, while the shape memory fixation rate remained above 93% and the shape self‑healing rate after three cycles exceeded 96%. Bio‑based vanillin enhances the mechanical strength and light‑response properties of the material through the synergism of the rigid aromatic ring and the dynamic imine bond.

**FIGURE 12:**
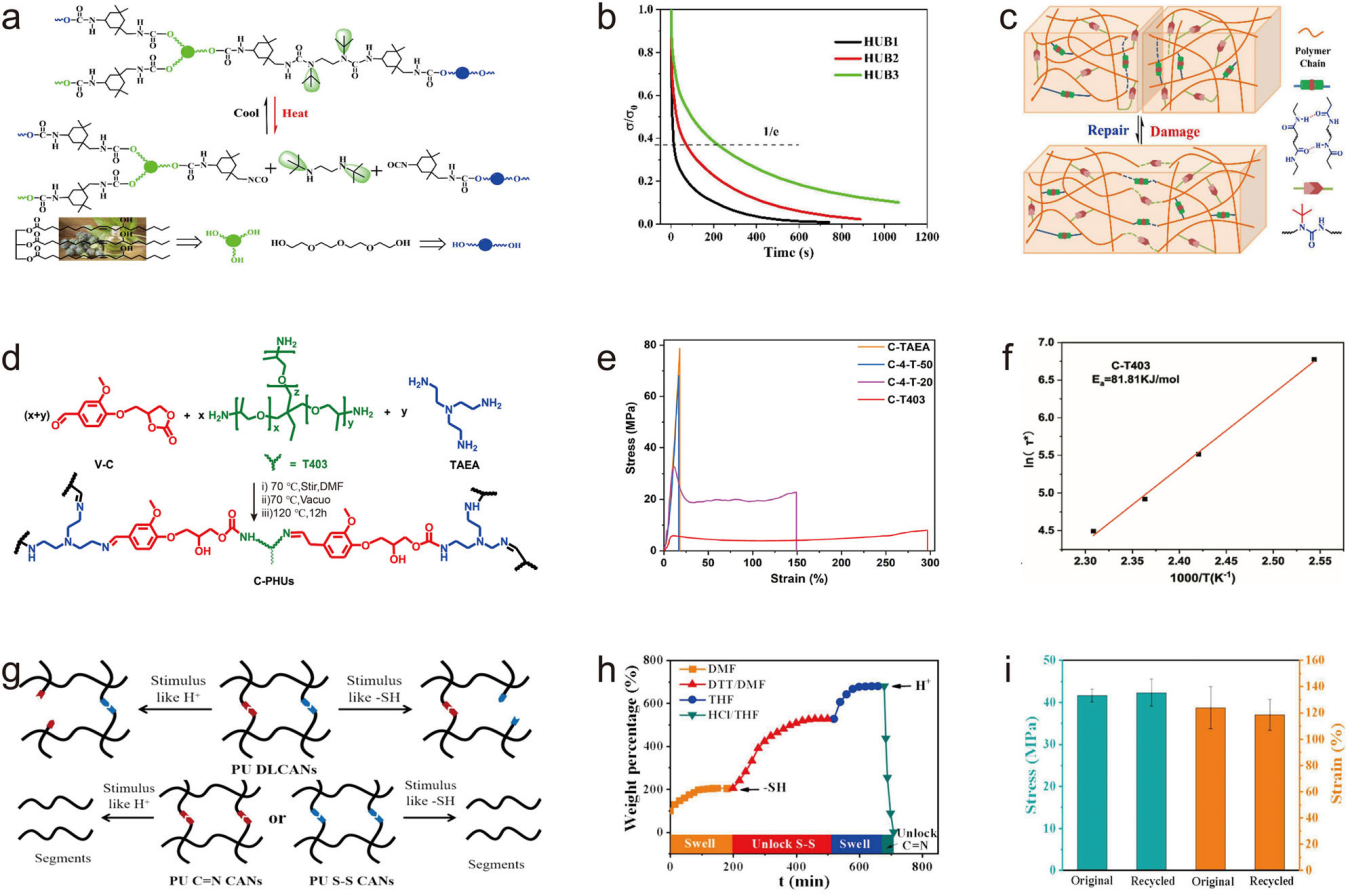
Mechanism and core data of bio‑based multiple dynamic bond synergy from structural design to functional expression. (a) Schematic of microscopic dynamic exchange between hindered urea bonds and hydrogen bonds. (b) Stress relaxation curves of elastomers with different formulations at 100°C. (c) Mechanism of synergistic repair via hydrogen‑bond recombination and hindered urea‑bond exchange. (a–c) Reproduced with permission [145]. Copyright 2022, Elsevier. (d) Synthesis of vanillin‑based cyclic carbonate and preparation of double dynamic network via ammonolysis polymerization with triamine. (e) Stress–strain curves of NIPU elastomers with different triamine ratios. (f) Arrhenius plot of stress relaxation time as a function of temperature. (d–f) Reproduced with permission [146]. Copyright 2025, Elsevier. (g) Parallel cross‐linking structure of disulfide and imine bonds in double‑lock network and partial fracture mechanism under single stimulus. (h) Programmed degradation kinetics of double‑lock network after sequential treatment with dithiothreitol and hydrochloric acid. (i) Stress–strain curves of double‑lock network elastomers before and after closed‑loop recovery. (g–i) Reproduced with permission [147]. Copyright 2024, Elsevier.

Double dynamic bond coordination can also be constructed based on the dynamic exchange of hindered urea bonds and hydrogen bonds. The reaction of tert‑butyl‑substituted secondary amines with isocyanates forms the hindered urea bond. Its reversible dissociation and recombination provide the driving force for the rearrangement of network topology. Zhang et al. [145] synthesized PU elastomers (HUB) with hindered urea bonds using castor oil as a bio‑based polyol. The microscopic process of the dynamic exchange of the hindered urea bond and the hydrogen bond is shown in Figure 12a. The long, fatty chain of castor oil made it easy to diffuse and move when heated, and its multi‑hydroxyl structure provided the necessary structural sites for the cross‐linking network. The stress relaxation time of HUB3 at 100°C was only 221 s, whereas that of HUB1 with lower cross‐linking density was shortened to 12.3 s (Figure 12b).

The synergistic repair principle of hydrogen bond recombination and hindered urea bond exchange is illustrated in Figure 12c: hydrogen bonds formed rapidly during heating, restoring the initial network structure, while the dissociation and reconstruction of the hindered urea bond at high temperatures completed the topological rearrangement. The tensile strength of the elastomer reached 20.7 MPa, and the elongation at break was 248.7%. The sample achieved nearly 100% scratch repair efficiency after 10 min at 100°C. The shape‑memory fixation rate exceeded 88.4%, and the recovery rate exceeded 81.3%. The mechanical properties of the elastomer did not decrease significantly after four cycles of hot‐pressing recovery. Bio‑based castor oil integrates mechanical toughening and multiple functions by regulating network flexibility and dynamic bond density.

The multiple dynamic bond synergism strategy is also effective in NIPU systems. The dynamic bond of HU is formed by the ring‑opening reaction of cyclic carbonate with the amino group. The imine bond is formed in situ by the condensation of an aldehyde and an amino group. As a bio‑based aromatic monomer, vanillin provides a modifiable skeleton for constructing two dynamic bonds. Fan et al. [146] prepared HU‑imine double dynamic network elastomers using vanillin‑derived cyclic carbonate as a precursor. The preparation of vanillin‐derived cyclic carbonate and its ammonolysis polymerization with a triamine are illustrated in Figure 12d. The rigid benzene ring of vanillin endows the network with an extremely high cross‐linking density and structural strength. The hydroxyl carbamate unit provides numerous pendant hydroxyl groups to form reinforcing hydrogen bonds. Under different triamine ratios, the tensile strength of C‑TAEA reached up to 79 MPa, and the maximum elongation at break of C‑T403 was 296% (Figure 12e). From the Arrhenius relationship between stress relaxation time and temperature of C‑T403, the dynamic bond‑exchange activation energy was fitted to be as low as 81.81 kJ mol^−1^ (Figure 12f). The imine bond is rapidly hydrolyzed under acidic conditions at room temperature to achieve complete degradation. The HU bond and the imine bond undergo topological rearrangement synergistically at 140°C. The recovery rate of the mechanical properties of the elastomer after hot‑press recovery was 91.65%. Bio‑based vanillin introduces two dynamic covalent bonds simultaneously through bifunctional design. The combination of a rigid aromatic ring and a double dynamic network achieves a balance between high strength and rapid stress relaxation.

The parallel connection mode of double dynamic bonds can endow the network with ultra‑stable characteristics and programmed degradation ability. The disulfide bond is sensitive to reducing thiols, and the imine bond undergoes rapid hydrolysis in acidic conditions, such that the two dynamic bonds share the network cross‐linking function in parallel. Zhou et al. [147] constructed a double‑lock covalent adaptive network by reacting castor oil‑derived isocyanate prepolymer with two dynamic amine curing agents. The structural mechanism of the double dynamic cross‐linking points formed by the disulfide bond and the imine bond in parallel is explained in Figure 12g. The aromatic disulfide bond was introduced by aminophenyl disulfide, and the aromatic imine bond was introduced via aminophenylimine. The long, fatty chain of castor oil endows the network with high flexibility and hydrophobicity. Programmed degradation kinetics after treatment with dithiothreitol followed by hydrochloric acid solution were recorded (Figure 12h). Under single stimulation, only some of the network's cross‐linking points broke, leaving it swollen but insoluble.

After the double stimulation was applied in sequence, the elastomer was completely degraded into soluble segments. The tensile strength of the recovered sample reached 42 ± 3 MPa, and the elongation at break was restored to 119 ± 12%, as shown by the stress‑strain curves of the original and closed‑loop recycled PU (Figure 12i). The imine bond absorbs ultraviolet light and can provide the coating with ultraviolet shielding. The disulfide bond and the imine bond can be exchanged synergistically and reversibly at 170°C to achieve self‑healing. Castor oil improves network toughness by regulating soft segment polarity and cross‐linking density. The parallel design of the double dynamic bonds enables the material to maintain an ultra‑stable cross‐linked structure in a conventional environment. The self‑healing function, programmed degradation, and closed‑loop recovery ability all depend on the bio‑based skeleton supporting the double‑lock network.

The above examples collectively illustrate that the synergistic combination of multiple dynamic bonds within a single bio‑based PU network offers distinct advantages over single dynamic bond systems, but each dual or multiple bond combination presents unique trade‑offs (Table 5). Disulfide‑hydrogen bond dual networks achieve self‑healing at sub‑zero temperatures, up to 92% efficiency at −10°C, with moderate tensile strength, making them particularly suitable for low‑modulus applications such as coatings and flexible adhesives where ambient‑temperature healing is critical. Imine‑hydrogen bond systems leverage the photothermal conversion capability of aromatic imine precursors, enabling light‑triggered, spatially controlled healing with 92.43% efficiency in 30 min under visible light. Hindered urea‑hydrogen bond networks offer rapid stress relaxation, with a characteristic time of 12.3 s at 100°C, and high reprocessing fidelity exceeding 95% property retention after three cycles. The disulfide‑imine dual‑lock design provides unique sequential programmed degradation, where orthogonal chemical stimuli enable controlled, stepwise network disassembly. However, a common challenge across all multiple dynamic bond systems is the difficulty of independently tuning the exchange kinetics of each bond, as modifications targeting one dynamic chemistry invariably affect the behavior of the other through changes in network topology and segmental mobility.

**TABLE 5:** Comparison of multiple dynamic bond systems in bio‐based polyurethane networks.

Dynamic bond combination	Self‐healing efficiency (%)	Tensile strength (MPa)	Key advantages & challenges	Key references
Disulfide + H‐Bond	∼90%	16.1 MPa Elongation: 1055.8%	• Most mature system; • Excellent elastic recovery • Multiple recyclability	[148]
Imine + H‐Bond	98% stress recovery (36°C, 20 min)	Tunable (hard segment ratio dependent)	• Body‐temperature self‑healing • Excellent mechanical properties • Closed‑loop recyclability	[149]
Hindered Urea + H‐Bond	88.9–100% (100°C, 10 min)	4–20 MPa	• Ultra‐fast stress relaxation; • Excellent reprocessability • Shape memory (fixity >88.4%, recovery >81.3%)	[145]
Hydroxycarbamate + Imine [NIPU; vanillin‐based]	Good reprocessability & self‑healing Acid‑triggered degradation	Up to 79 MPa Elongation: 296%	• Isocyanate‐free NIPU route Excellent mechanical properties • Room‑temperature acid degradation	[146]
Disulfide + Imine	Self‐healing properties reported	CO‐based PU thermosets	• Programmed degradation • Closed‑loop recycling • Ultra‑stable against adverse stimuli • UV shielding + long‑term stability	[147]

Despite these advances, several critical challenges remain for the practical deployment of multiple dynamic bond systems. The long‑term stability of multiple dynamic networks under realistic service conditions, including cyclic mechanical loading, temperature and humidity cycling, and chemical exposure, requires systematic evaluation. Furthermore, methods for independently characterizing the contribution of each dynamic bond type to macroscopic properties, for instance through selective bond labeling or kinetic isotope effects, need to be developed. Finally, the multi‑step, stoichiometrically precise synthesis required for multicomponent dynamic networks presents a significant barrier to industrial‑scale production, as the complexity of incorporating two or more dynamic chemistries into a single network increases production costs and quality control demands.

### 3.4 Construction of Nanocomposites

Nanocomposite engineering provides an effective route to enhance the overall performance of bio‑based PU. The introduction of nanofillers enhances the mechanical strength and imparts additional functionalities to bio‑based PU. The size and surface effects of nanofillers can enhance interfacial interactions, and this interface strengthening mechanism promotes the transfer of stress from the matrix to the filler [33]. Furthermore, nanofillers can endow PU with self‑healing, thermal conductivity, flame retardancy, or electrical conductivity. The selection of functional nanofillers directly determines the target application of composite materials [150, 151]. For example, zero‑dimensional nanomaterials (nanoparticles, carbon dots, quantum dots, etc.) can form a conductive network or increase cross‐linking density; one‑dimensional nanomaterials (nanofibers, nanowires, carbon nanotubes (CNTs), etc.) can form percolation structures and limit the movement of molecular chains; two‑dimensional nanomaterials (BNNSs, graphene, MXene, etc.) can serve to construct thermal conduction pathways and block gas penetration [152, 153, 154]; three‑dimensional nanomaterials (metal‑organic frameworks (MOFs), covalent organic frameworks (COFs), zeolites, etc.) can serve to adsorb gases and catalyze char‑forming reactions [155]. Therefore, by regulating the intrinsic properties and dispersion state of nanofillers, researchers can accurately design the mechanical response and functional expression of composites.

Nanocellulose is an important nanofiller to improve the mechanical properties of bio‑based PU. CNCs have high crystallinity, high modulus, and biodegradability [156]. Tunicate CNCs (TCNCs) are particularly prominent due to their high aspect ratio of 94 and modulus of about 12.0 GPa. Deng et al. [157] introduced TCNCs into castor oil‑based WPU matrix by solution blending. The hydroxyl groups on the surface of TCNCs formed a strong hydrogen bond interface with WPU urethane bonds, and the interfacial interaction enhanced the cross‐linking density and *Tg* of the composites. TCNCs constructed a rigid percolation network in WPU to effectively transfer stress. The tensile strength increased from 7.36 MPa for pure WPU film to 18.29 MPa after adding 10 wt.% TCNCs, while the Young's modulus increased significantly from 25.13 to 312.17 MPa. The elongation at break remained above 110%. The storage modulus rose from 62.06 to 903.32 MPa at 25°C. The composite film's toughness reached a maximum of 28 MJ m^−^^3^ at a TCNC content of 5 wt.%. Dynamic mechanical analysis and DSC confirmed that *Tg* increased with increasing TCNCs. Thermogravimetric analysis showed that the initial decomposition temperature increased from 252.85°C to 265.49°C. The above enhancement mechanism originates from the high‑modulus intrinsic characteristics of TCNCs and efficient interfacial stress conduction.

**FIGURE 13:**
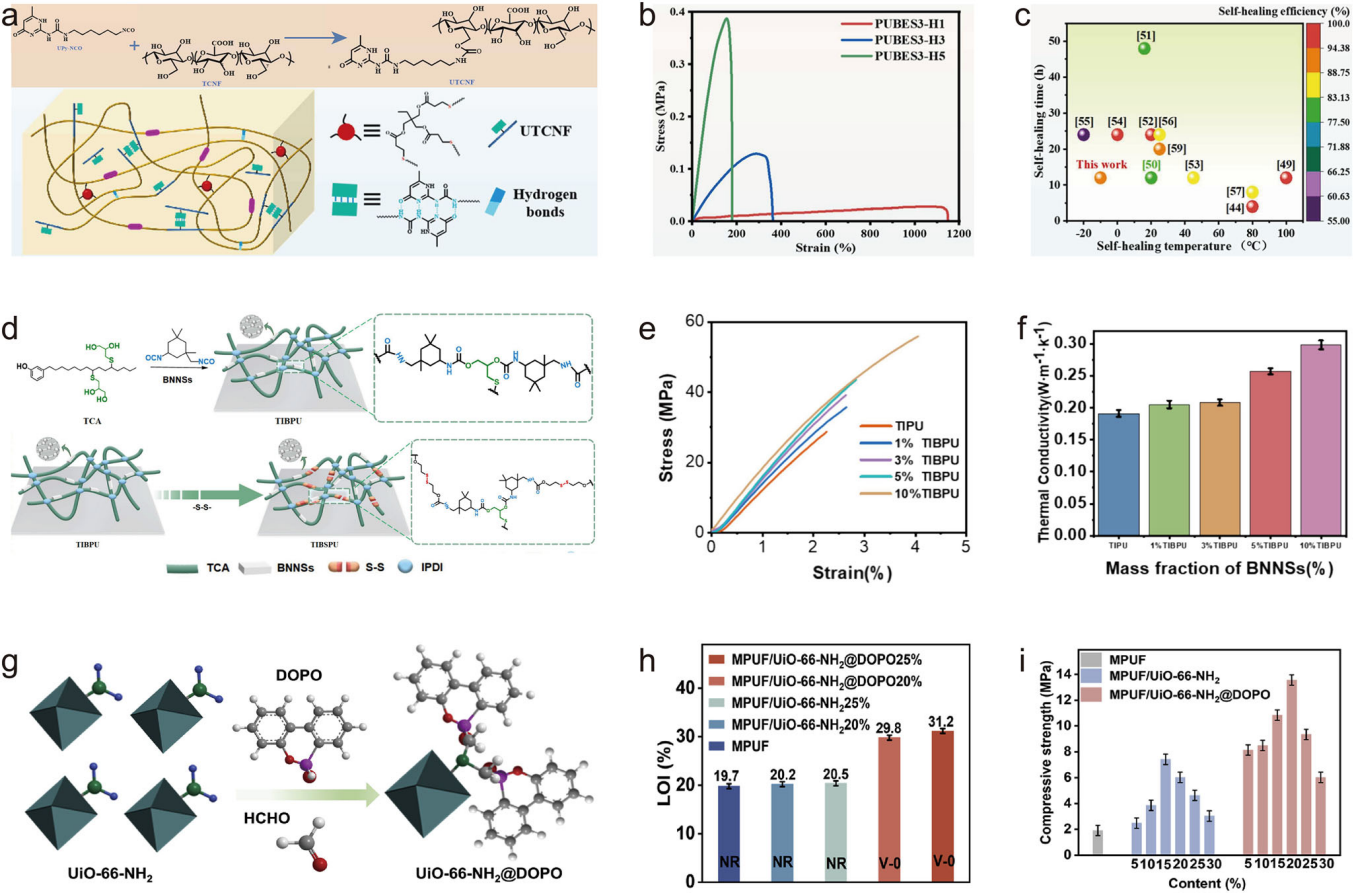
Nanocomposite strategy for improving the performance of bio‑based polyurethane materials. (a) Schematic diagram of the double‑dynamic supramolecular network structure composed of UTCNF and disulfide bonds. (b) Stress–strain curves of PUBES‑H composites with different UTCNF contents. (c) Comparison of self‑healing time, temperature, and efficiency between PUBES3‑H1 and other reported self‑healing elastomers. (a–c) Reproduced with permission [158]. Copyright 2025, Elsevier. (d) Synthesis roadmap of cardanol‑based polyurethane and BNNSs composites. (e) Tensile stress–strain curves of polyurethane films with different BNNSs contents. (f) Thermal conductivity of polyurethane film composites with different BNNSs contents. (d–f) Reproduced with permission [106]. Copyright 2024, Elsevier. (g) Preparation of UiO‑66‑NH_2_@DOPO nano flame retardant via reaction of UiO‑66‑NH_2_ and DOPO. (h) Limiting oxygen index comparison of pure MPUF and composites with different flame retardant contents. (i) Compressive strength comparison of pure MPUF and its composites at 90% strain. (g–i) Reproduced with permission [159]. Copyright 2025, Elsevier.

The introduction of nanofillers not only improves mechanical properties but also endows bio‑based PU with dynamic self‑healing function. The realization of self‑healing ability depends on the reversible cleavage and recombination of dynamic covalent bonds and supramolecular interactions. Cellulose nanofibers (CNFs) can provide multiple hydrogen bond cross‑linking sites after functional modification. Wang et al. [158] constructed a dual dynamic network by introducing ureidopyrimidinone‑modified CNFs (UTCNF) into a bio‑based PU matrix. The UPy group on the surface of UTCNF formed quadruple hydrogen bond cross‑linking points with the PU hard segments, and the disulfide bonds and hydrogen bonds in the main chain constituted a dynamic supramolecular network (Figure 13a). After adding 5 wt.% UTCNF, the tensile strength of PUBES3‑H5 increased from 0.06 to 0.32 MPa, more than five times that of the unmodified sample (Figure 13b). At the same time, the elongation at break of the composite elastomers exceeded 700%. The UTCNF induced orientation of the PU main chain during stretching, and its strain‑induced crystallization effect further promoted hydrogen bond recombination and network strengthening. The self‑healing efficiency reached 93% after healing for 12 h at −10°C (Figure 13c). The dynamic cross‑linking points formed by UTCNF do not hinder the low‑temperature mobility of the chain segments. The *Tg* of the material is about −13°C, which ensures the molecular chain's low‑temperature activity. The broken disulfide bonds and hydrogen bonds recombine rapidly at the low‑temperature interface. The physical entanglement of nanofibers and interfacial hydrogen bonds jointly promote crack healing.

Thermal management is a key requirement for polymer materials in electronic device packaging and heat dissipation. The polymer's low intrinsic thermal conductivity limits its application in high‑power applications. BNNSs have both high thermal conductivity and electrical insulation. Sha et al. [106] introduced BNNSs into a cardanol‑based PU matrix to prepare thermally conductive composites (TIPU). As presented in Figure 13d, BNNSs were uniformly dispersed in PU prepolymer by solution blending, and the hydroxyl groups on the surface of BNNSs formed a strong interfacial interaction with the PU hard segments. The tensile strength of pure PU TIPU was 28.86 MPa, and it increased to 54 MPa after adding 10 wt.% BNNSs, while the elongation at break rose from 2.5% to 4.27% (Figure 13e). The mechanical properties and high specific surface area of BNNSs are the key to enhancement. Uniformly dispersed BNNSs constructed a continuous thermal conduction pathway in the matrix. The thermal conductivity of pure TIPU was only 0.191 W m^−^^1^ K^−^^1^, and that of the composite containing 10 wt.% BNNSs was 0.298 W m^−^^1^ K^−^^1^, which was 56% higher than that of pure PU (Figure 13f). The BNNS thermal‑conductive network can effectively promote heat transfer. The higher the BNNS content, the higher the thermal conductivity. The dynamic disulfide bond simultaneously endows the composite material with self‑healing and reprocessing capabilities.

Flame retardancy and smoke suppression are prerequisites for the application of bio‑based PU in construction and transportation. Traditional flame retardants require a large loading and are prone to deteriorating mechanical properties. MOFs have emerged as a promising nano‑flame retardant due to their high specific surface area and designability. Zhang et al. [159] introduced functionalized UiO‑66‑NH_2_@DOPO nanofillers into a bio‑based modified porous PU foam (MPUF) matrix. UiO‑66‑NH_2_ is an amino‑functionalized zirconium‑based MOF. The composite route for UiO‑66‑NH_2_ and DOPO is shown in Figure 13g. The nanofiller was uniformly dispersed in MPUF and formed strong interfacial hydrogen bonds. The limiting oxygen index of pure MPUF was only 19.7%, and it increased to 31.2% after adding 25 wt.% UiO‑66‑NH_2_@DOPO, thus passing the UL94 V‑0 rating (Figure 13h).

The total smoke release decreased by 80.64%. The porous structure of UiO‑66‑NH_2_ adsorbed combustible gases and inhibited smoke emission. The presence of zirconium ions promoted the formation of a carbon layer in the matrix, thereby further increasing the residual carbon content. The decomposition of DOPO released phosphorus‑containing radicals to quench the free radicals in the combustion process and terminate the chain reaction. At 90% strain, the composite's compressive strength was 13.56 MPa, which is 7–8 times that of pure MPUF (Figure 13i). The hydrogen bonds between the nanofiller surface and the PU hard segments enhanced the energy dissipation. The composite material exhibited a maximum loss factor of 1.32 at 1.0 Hz. The interface interaction prolonged the stress propagation path and improved the vibration reduction performance.

The conductive function makes bio‑based PU applicable to flexible sensors and wearable electronic devices. Conductive nanofillers mainly include carbon black (CB), CNTs, graphene, and metal nanowires. CB is cheap and easy to disperse in a polymer matrix. Li et al. [160] introduced CB into a soybean oil‑based NIPU matrix. CB nanoparticles constructed a three‑dimensional conductive network in polysiloxane‑modified NIPU. The physical entanglement between CB and polymer chains enhanced the mechanical properties of the composites. The tensile strength increased from 0.095 MPa for pure NIPU to 1.0 MPa after adding 14 wt.% CB, nearly ten times higher. The uniform dispersion of CB effectively limited the slip of the PU molecular chain. The conductive network increased the composite material's conductivity to 27.8 mS cm^−^^1^. The dynamic urethane bond endowed the material with self‑healing ability at 120°C for 2 h. The conductivity retention rate after self‑healing was 93.2%. The polysiloxane segment lowered the material's Tg to −112°C. The composites still maintained 430% elongation at break at −40°C. The sensor output a stable resistance signal over the range of ‐40°C to 60°C.

The examples discussed above illustrate that the reinforcement efficiency of different nanofiller types varies considerably with dimensionality and must be evaluated alongside their trade‑offs with other critical properties, particularly recyclability (Table 6). Zero‑dimensional nanofillers, such as silica nanoparticles, carbon quantum dots, and metal oxide nanoparticles, provide moderate reinforcement at low loadings of 1 to 5 weight percent with minimal impact on optical transparency and melt processability, making them suitable for optically clear coatings; however, their reinforcement efficiency per unit loading is the lowest among all dimensionalities. One‑dimensional nanofillers, including CNCs, chitin nanowhiskers, and CNTs, offer high reinforcement efficiency through percolation network formation, leveraging the excellent intrinsic mechanical properties of individual nanofillers, often necessitating surface functionalization strategies. Two‑dimensional nanofillers, such as BNNSs, graphene, and MXene, provide the highest reinforcement per unit loading owing to their ultrahigh aspect ratios and specific surface areas, while simultaneously enabling multifunctional properties; however, their anisotropic properties demand orientation control for optimal performance, and high loadings above 5 weight percent can severely hinder dynamic bond exchange, reducing self‑healing efficiency by 40 to 60 percent. The trade‑off between mechanical reinforcement and reprocessability therefore represents a critical design consideration for nanocomposite bio‑based PUs.

**TABLE 6:** Comparative performance of nanofillers in bio‐based polyurethane nanocomposites.

Nanofiller (dimensionality)	Examples	Strength improvement	Additional functionality	Sufficient for real applications	Key references
0D — NPs	SiO_2_, TiO_2_, ZnO NPs	1.2–2.0× TiO_2_ (4 wt.%): tensile modulus ↑55% (14.85→23 GPa)	• UV‐shielding (TiO_2_) • Antibacterial (ZnO)	Coatings: ✓ Biomedical: ✓ Structural: ✗	[161]
1D — Nanofibers / Nanotubes	CNCs, TCNCs, CNFs; CNTs,	CNC: tensile strain 1740.42%, toughness 90.01 MJ/m^3^	• High specific strength • Conductivity (CNTs) • Biodegradability (CNCs)	Coatings: ✓✓ Structural: ✓ Sensors: ✓	[162]
2D — Nanosheets	BNNS, graphene oxide (GO), rGO, MXene (Ti_3_C_2_T_x_)	MXene (0.077 wt.%): tensile strength ↑39.9%, elongation ↑38.5%	• Thermal conductivity • EMI shielding (MXene) • Gas barrier (GO)	Coatings: ✓✓ Fire retardant: ✓✓ Energy: ✓✓	[163]
3D — Networks / Frameworks	MOFs (UiO‐66, ZIF‐8); Aerogels; COFs	UiO‐66 (30 wt.%): best mechanical properties	• Gas adsorption • Ultra‐low density • Tunable porosity	Specialty only: Gas separation: ✓ Catalysis: ✓	[164]

## 4 Functional Coupling and Advanced Application of Bio‐Based Polyurethane

### 4.1 Coatings and Protection

Coating technology provides a long‑term protective barrier for the substrate surface. Traditional petroleum‑based coatings rely on organic solvents and are difficult to degrade. Bio‑based PU coatings replace fossil raw materials with renewable polyols to achieve sustainability. Thanks to their strong designability, their molecular structures are conducive to synergistic improvements in mechanics and functionality [20, 165]. The regulation of hard‑segment content and cross‐linking density in bio‑based PU can optimize the coating's barrier properties and adhesion, and the introduction of hydrogen bonds and dynamic covalent bonds endows the coating with self‑healing ability. The bio‑based platform can also integrate functions such as ultraviolet absorption, low surface energy, and corrosion sensing.

Anticorrosion is the most basic and critical requirement in coating performance. The coating needs to establish a dense barrier between the environment and the substrate to prevent the penetration of water and oxygen, which can cause chemical corrosion [53]. The introduction of bio‑based rigid monomers can simultaneously increase cross‐linking density and hydrogen‑bond network density, thereby enhancing anticorrosion performance. Using castor oil as a flexible backbone, Shen et al. [166] copolymerized isosorbide and L‑tyrosine‑derived cyclic dipeptide (L‑CD) as rigid diols into the WPU backbone. The isosorbide (IS) double‑ring structure increased the hard‑segment content to 62.5% and the tensile strength from 15.97 to 29.56 MPa. The piperazinedione skeleton of L‑CD contributed multiple hydrogen bond sites that maintained the toughness at 20.12 MJ m^−^^3^ and retained 84.59% of the elongation at break.

Hydrogen bond enhancement was confirmed by shifts in the infrared peaks of carbamate and amide carbonyl groups to lower wavenumbers, and the stress–strain curves showed that the film transitioned from flexible to rigid as the IS ratio increased. Compared with the reported castor oil‑based WPU, the material balanced both mechanical properties and toughness. The water contact angle of the coating increased from 84.3° to 93.7°, and no significant whitening was observed after 72 h of immersion. Electrochemical tests showed that the corrosion inhibition efficiency of the L‑CD modified coating on 45# steel was 96.61%. The rigid bio‑based ring compresses the cross‐link spacing and strengthens intermolecular physical cross‐linking, limiting the path for water molecule penetration. This strategy improves the protection level of vegetable oil‑based WPU to a level comparable to that of a petrochemical‑based system.

**FIGURE 14:**
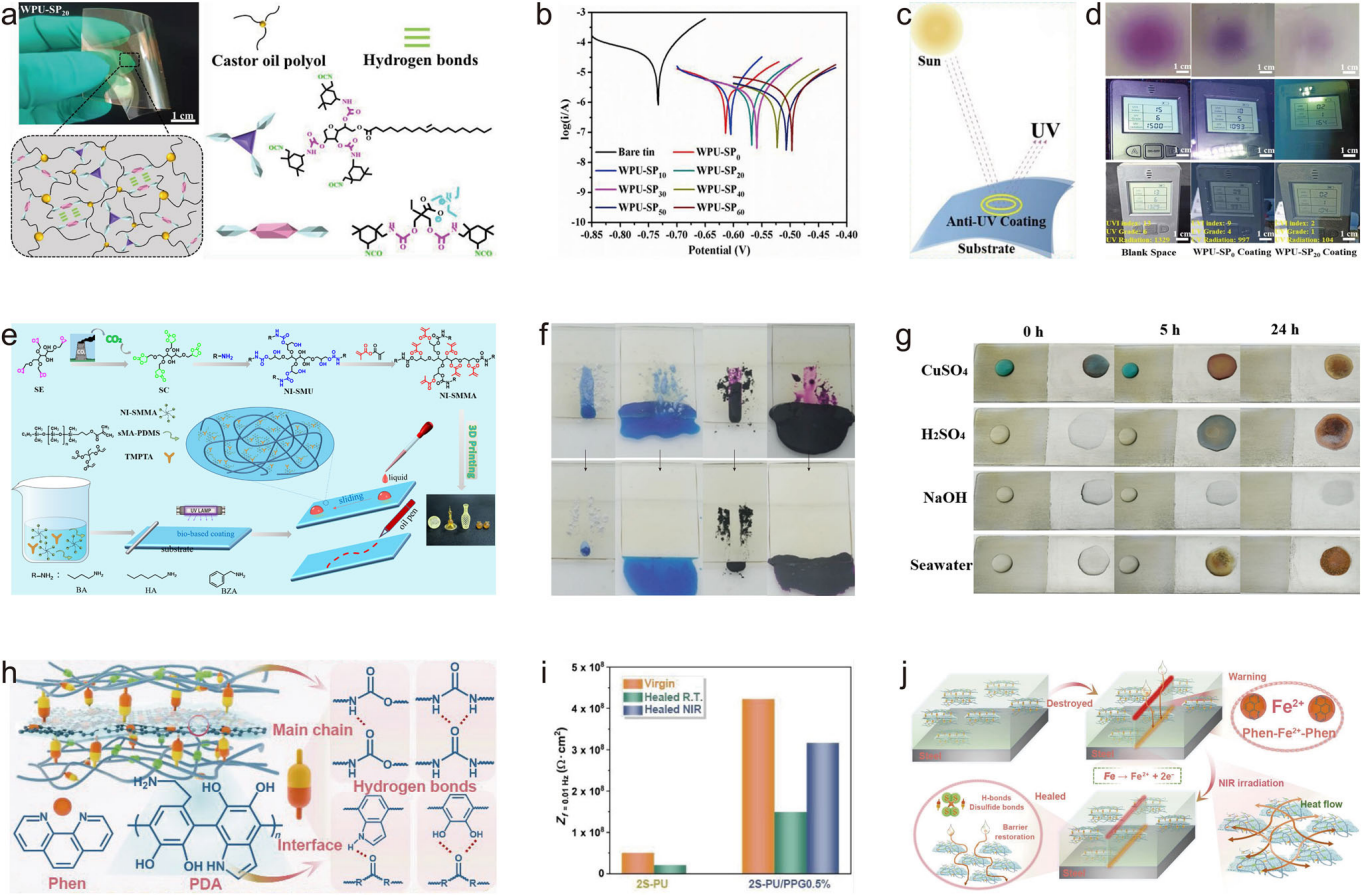
Development of bio‑based polyurethane coatings from structural design and multi‑functional integration to response. (a) Physical photos and network structure of WPU‑SP_20_ film. (b) Tafel polarization curves of WPU coatings with different SP contents. (c) Schematic diagram of the anti‑ultraviolet mechanism of the coating. (d) UV test card and radiometer quantitative results demonstrating the actual UV shielding effect of SP coating. (a–d) Reproduced with permission [167]. Copyright 2022, Elsevier. (e) Synthesis route of NI‑SMUV coating and schematic diagram of the 3D printing process. (f) Comparison of sliding behavior of water, milk, corn oil, and pump oil on unmodified (left) and 1 wt.% monomethacryloxypropyl polydimethylsiloxane modified (right) coating surfaces. (g) Corrosion test photos of NI‑SBZ‑1 coating and bare tin plate exposed to CuSO_4_, H_2_SO_4_, NaOH, and seawater for 0, 5, and 24 h. (e‐g) Reproduced with permission [168]. Copyright 2025, Elsevier. (h) Schematic diagram of the internal dynamic bond network of the bionic composite material. (i) Low‑frequency impedance modulus contrast histogram of the coating in 3.5 wt.% NaCl solution after initial damage and repair. (j) Schematic diagram of the protective mechanism of the bionic coating, illustrating the barrier effect, self‑healing, and Fe^2^^+^ chelating color warning process. (h–j) Reproduced with permission [169]. Copyright 2023, Tsinghua University Press.

In addition to general corrosion damage, coating protection must also resist aging degradation caused by ultraviolet irradiation when used outdoors. Ultraviolet light can induce free radical oxidation, leading to polymer chain breakage and accelerating coating failure. Endowing the coating with intrinsic UV absorption capacity can prolong service life and reduce reliance on external light stabilizers. The bio‑based furan ring structure has natural ultraviolet absorption characteristics and can be used as a rigid block to enhance network density. Deng et al. [167] prepared a series of WPU by copolymerizing castor oil with sorbitan monooleate (SP), a bio‑based polyol. The SP molecule contained a furan ring, an ortho‑trihydroxyl group, and a dangling oleic acid chain. The furan ring enhanced the rigidity of the hard segments and increased the hydrogen‑bonded carbonyl ratio to 70.4%. The cross‐linking density increased from 325.99 mol m^−^^3^ to 365.61 mol m^−^^3^. The tensile strength of WPU‑SP50 reached 26.32 MPa, and the modulus increased to 554.85 MPa (Figure 14a). The elongation at break was retained at 56.36% due to the internal plasticization of the dangling fatty acid chain.

Electrochemical polarization curves showed that the WPU‑SP50 coating increased the corrosion potential to −0.507 V and decreased the corrosion current to 2.92 × 10^−^^6^ A, yielding a corrosion inhibition efficiency of 95.39% (Figure 14b). The coating did not rust on the metal substrate after 6 months of outdoor exposure. UV transmittance spectra showed that all films containing SP exhibited 0% transmittance in the UVC region, while WPU‑SP50 completely blocked 200–380 nm radiation in the UVA region (Figure 14c,d). In the outdoor UV irradiation comparative test, the test paper coated with WPU‑SP20 did not change color, whereas the bare test paper was significantly yellowed. Bio‑based SP simultaneously provides UV absorption and a dual shielding mechanism based on a dense cross‐linked network.

Self‑cleaning and anti‑graffiti functions require extremely low surface energy on the coating surface. The liquid needs to have a high contact angle on the coating surface and be easy to slide to resist the adhesion of pollutants. Traditional fluorocarbon modification is effective, but its environmental persistence is uncertain. The bio‑based skeleton, combined with fluorine‑free hydrophobic segments, can yield an environmentally friendly antifouling surface. Liu et al. [168] synthesized sorbitol cyclic carbonate (SC) by an insertion reaction of a sorbitol‑based epoxy precursor and CO_2_, and then obtained non‑isocyanate sorbitol‑based multi‑urethanes by ring opening with monoamine, followed by blending with monomethacryloxypropyl polydimethylsiloxane and UV curing after methacrylic anhydride esterification (Figure 14e).

The bio‑based sorbitol skeleton provides a polyhydroxyl platform, eliminating the need for isocyanates. Water droplets, milk, corn oil, and pump oil on the surface of the coating containing 1 wt.% monomethacryloxypropyl polydimethylsiloxane all shrank into hemispheres and slipped without residue (Figure 14f). The water contact angle increased from 52.1° to 109.3°, and the hexadecane contact angle increased from 10.9° to 45.2°. The surface energy decreased from 50.4 to 20.3 mJ m^−^^2^, and the polar component decreased from 27.1 to 20.1 mJ m^−^^2^. No corrosion was observed on the surface of the coating after immersion in 0.5 mol L^−^^1^ CuSO_4_ solution, H_2_SO_4_ (pH 0), NaOH (pH 14), and seawater for 24 h (Figure 14g). This phenomenon can be attributed to the enrichment of silicon‑containing segments at the interface, forming a dense, extremely low surface energy barrier. The bio‑based non‑isocyanate system combines a renewable carbon skeleton, rapid photocuring, and long‑term anti‑graffiti performance on a single coating platform.

High‑end coatings need both self‑healing against damage and early warning capabilities. Once the traditional coating is scratched, it loses its protective function, and the damaged location is difficult to find. Dynamic bond recombination film formation and corrosion response discoloration are two core paths to achieve active protection. Wu et al. [169] coated graphene oxide with polydopamine and loaded 1,10‑phenanthroline to form polydopamine‑coated graphene oxide nanosheets, which were then assembled with the PU matrix through interfacial hydrogen bonds to form a pearl‑like ordered structure (Figure 14h; dynamic bond network diagram). The tensile strength of the bio‑based elastomer was 6 MPa, and the elongation at break was maintained at 2320%, with a toughness of 116.1 MJ m^−^^3^. The low‑frequency impedance modulus of the damaged coating increased to 2.07 × 10^7^ Ω·cm^2^ after repair at room temperature for 3 h. Near‑infrared light irradiation for 1 h restored the impedance modulus to 3.2 × 10^6^ Ω·cm^2^, which was close to the initial value (Figure 14i). The Fe^2^^+^ ions released from the Phen‑chelated corrosion site after the coating was scratched produced a striking red warning signal (Figure 14j). The bio‑based soft segment endows the network with ultra‑long stretching ability and achieves efficient self‑healing by promoting dynamic bond recombination. The bionic layered structure can prolong the diffusion path of corrosive medium and enhance the passive protection ability. The strategy integrates the renewable carbon skeleton of cashew nut shell oil and its active damage‑warning function into a single protective coating platform.

### 4.2 Fire Protection

PU foams and elastomers are widely used in building energy conservation and electronic devices due to their good thermal insulation and machinability. However, traditional PU is highly dependent on petroleum‑based resources and is inherently flammable, releasing large amounts of heat and toxic gases during combustion [170]. The conventional strategy to improve the fire safety of PU is to add halogen or phosphorus‑containing flame retardants. However, these additives pose hidden risks of migration and precipitation, reducing the mechanical properties of the matrix and raising concerns about their environmental persistence [171]. Therefore, the development of intrinsically flame‑retardant, renewable, bio‑based PU is urgently needed. Biomass materials such as lignin, phytic acid, and vanillin are naturally rich in aromatic rings and oxygen‑containing functional groups, which can serve as ideal anchoring platforms for phosphorus‑containing flame‑retardant structures [172, 173]. These bio‑based components are covalently incorporated into the PU main chain, and the flame‑retardant elements are stably locked within the network, avoiding the migration problems associated with additive flame retardants [174]. During combustion, the aromatic skeleton is converted into a dense carbon layer. The phosphorus element promotes cross‑linking in the condensed phase to form carbon while releasing free radicals in the gas phase to quench the combustion chain reaction. The above synergistic mechanism enables bio‑based PU to achieve high‑efficiency flame retardancy and heat insulation while maintaining, or even improving, mechanical properties.

**FIGURE 15:**
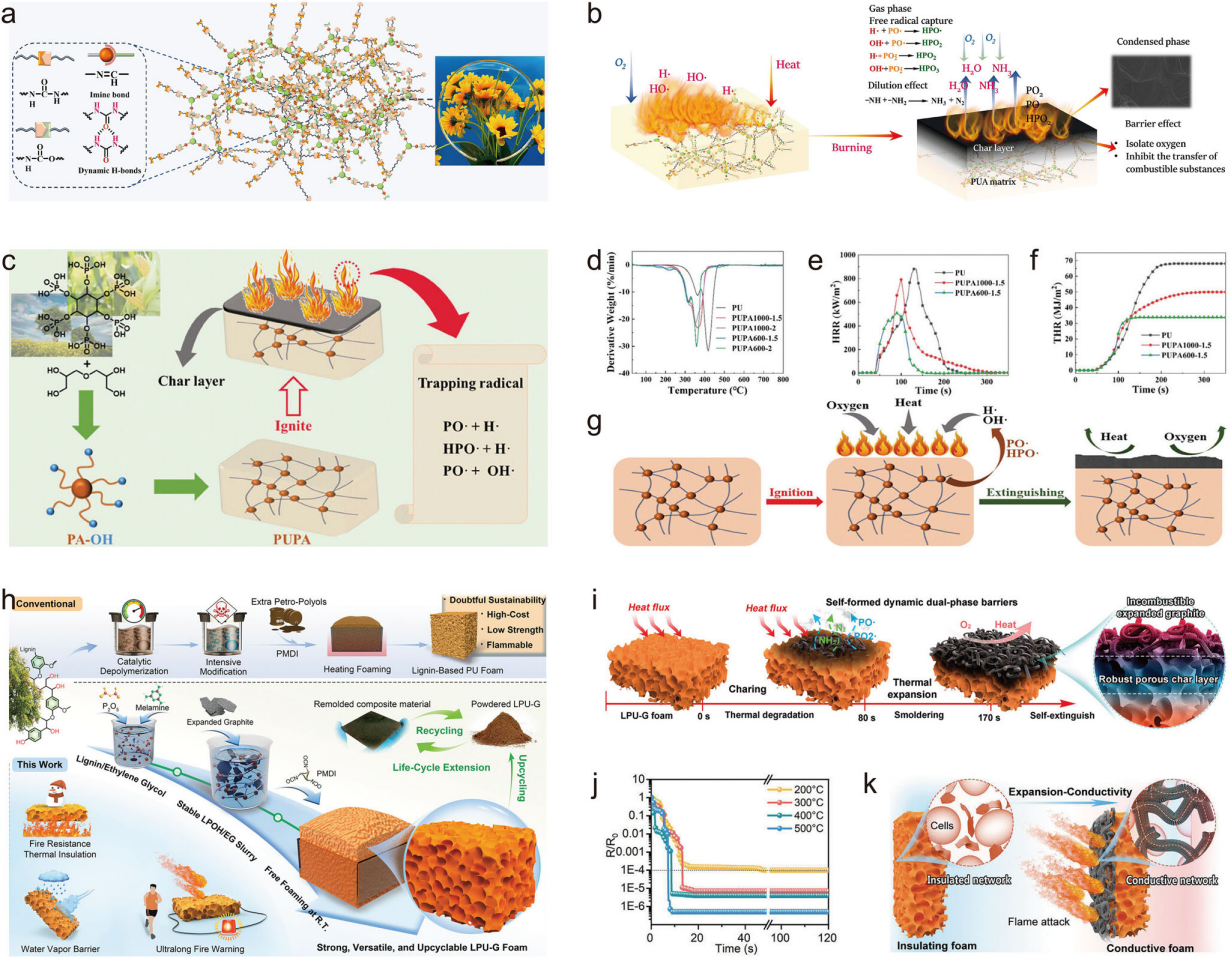
Multi‑level design strategy and internal principles for achieving flame‑retardant, thermal‑insulating, and early‑warning functions in bio‑based polyurethane. (a) Molecular structure of phosphorus‑containing vanillin derivatives forming polyurethane urea elastomers. (b) Schematic of the two‑phase flame‑retardant mechanism of vanillin‑based polyurethane urea elastomer during combustion. (a,b) Reproduced with permission [175]. Copyright 2025, Elsevier. (c) Synthesis route of PUPA. (d) Differential thermogravimetric curve in nitrogen atmosphere. (e) Heat release rate curve in cone calorimeter test. (f) Total heat release versus time in cone calorimeter test. (g) Schematic diagram of the two‑phase flame retardant mechanism. (c–g) Reproduced with permission [178]. Copyright 2025, Wiley‐VCH. (h) Synthesis roadmap of expandable graphite polyurethane foam. (i) Schematic of the dynamic two‑phase flame retardant mechanism involving synergistic formation of an expanded carbon layer and release of phosphorus‑containing free radicals during combustion. (j) Decrease in resistance with time under flame attack at different temperatures. (k) Schematic of the expansion‑conduction fire early‑warning mechanism based on the continuous conductive network formed by thermal expansion of expandable graphite. (h–k) Reproduced with permission [179]. Copyright 2024, Wiley‐VCH.

The microphase separation structure of PU elastomer endows it with high strength and toughness. However, the inherent flammability of PU materials severely limits their use in electronic packaging and protective equipment. By synergistically introducing a dynamic covalent bond system and a phosphorus‑containing biomass monomer, flame retardancy and recyclability can be simultaneously achieved while maintaining the elastomer's high resilience. Ding et al. [175] synthesized two phosphorus‑containing oxime polyols (TMPOH and HVPOH) with vanillin and incorporated them into the main chain of PPU to construct a dynamic oxime‑PU bond and a hydrogen‑bond synergistic network (Figure 15a). This strategy couples the aromatic skeleton of biomass with dynamic covalent bonds, providing a new paradigm for elastomer fire protection and closed‑loop recovery. The tensile strength of the elastomer PUA‑TMPOH reached 50.9 MPa, and the limiting oxygen index increased with increasing phosphorus content. In the cone calorimeter test, the peak heat release rate and total heat release of PUA‑HVPOH were 23.6% and 30.5% lower than those of pure samples, respectively. The flame‑retardant mechanism is presented in Figure 15b: phosphorus‑containing vanillin derivatives formed a dense, phosphorus‑rich carbon layer in the condensed phase, inhibiting heat transfer, while phosphorus‑containing free radicals were released in the gas phase to capture active hydrogen and hydroxyl radicals, thereby terminating the combustion chain reaction. The aromatic structure of bio‑based vanillin enhances the stability of the carbon layer, and the dynamic oxime‑PU bond confers renewability on the material, with mechanical property retention exceeding 95% after multiple dissolution and recovery cycles.

When collecting mechanical energy, triboelectric nanogenerators usually require an elastic dielectric material as a contact layer [176, 177]. Conventional polymers are easy to burn, and fire spread is uncontrollable, posing a safety hazard when such equipment is used. The application of an intrinsic flame‑retardant, bio‑based PU to the triboelectric layer enables integration of power‐generation and fire‐prevention functions without adding flame retardants. Zhang et al. [178] synthesized a phytic acid‑based polyol (PA‑OH) from phytic acid and diglyceride as bio‑based raw materials, and then prepared an intrinsic flame‑retardant PU elastomer (PUPA) (Figure 15c).

The phytic acid molecule contains six phosphate groups and can be directly incorporated into PU copolymerization as a high‑density phosphorus source. Thermogravimetric analysis showed that the degradation rate of PUPA decreased in the high‑temperature region, and the carbon residue increased significantly (Figure 15d). The cone calorimeter test confirmed that the peak heat release rate and total heat release of PUPA600‑1.5 were significantly reduced (Figure 15e,f). The flame‑retardant mechanism (Figure 15g) involves the pyrolysis of the phytic acid structure in the condensed phase to produce phosphoric acid and polyphosphoric acid, which catalyze the dehydration of the matrix into carbon. In contrast, the gas phase releases phosphorus‑containing free radicals to quench the active radicals. The elastomer reached UL94 V‑0 grade and exhibited a limiting oxygen index of 28.8%, and a fracture toughness of 34.71 MJ m^−^^3^. Bio‑based phytic acid is covalently incorporated into the network to prevent the migration of flame retardants, providing reliable, elastic dielectric materials for self‑powered sensing devices in high‑risk fire scenarios.

Rigid PU foam is the core material for building exterior walls and pipeline insulation, but its porous structure makes it highly flammable, and flames spread rapidly [170]. Using biomass‐liquefied polyol instead of petrochemical polyol can reduce carbon emissions, but it is necessary to incorporate flame retardants to meet the commercial fire rating. Nguyen‐Ha et al. [180] used liquefied bamboo powder as a bio‑based polyol to prepare flame‑retardant rigid foams by compounding nano‑silica derived from rice husk with halogen‑free flame retardants (aluminum diethylphosphinate (OP), diammonium hydrogen phosphate (DAP), aluminum hydroxide (ATH)). The residual lignin components in the bamboo‑derived polyol imparted the foam with an initial tendency toward carbonization and thermal stability, while nano‑silica optimized the cell structure and reduced the combustion rate. With the addition of the flame retardants, the limiting oxygen index of the foam increased to 23–26% and passed the UL94 V‑0 level. The cone calorimeter test showed that the peak heat release rate was 32.37–41.68% lower than that of pure bio‑based foam. DAP and OP released phosphorus‑containing free radicals in the gas phase, while ATH absorbed heat and dehydrated in the condensed phase, forming a protective alumina layer. The thermal conductivity of the foam was maintained at 0.038–0.046 W m^−^^1^ K^−^^1^, which is lower than that of most biomass insulation boards. The synergism of bio‑based polyol and halogen‑free flame retardants enables efficient integration of flame‑retardant and thermal‑insulation properties in foams, providing a solution for building fire protection and insulation.

The active fire warning function can trigger an alarm at the start of a fire, buying time for evacuation [181, 182]. Based on the principle that the volume of expandable graphite changes during heating, forming a conductive path, it is incorporated into lignin‑based PU foam to enable fire sensing via resistance transition. Sun et al. [179] proposed a one‑pot method to prepare lignin phosphate‑based PU/expandable graphite composite foam (Figure 15h). The high solubility of lignin in ethylene glycol makes it unnecessary to depolymerize under high pressure. The obtained lignin phosphate polyol exhibited both flame‑retardant and dynamic ester‑exchange functions. The foam remained non‑combustible at 1200°C, and the back temperature was only 31°C. The flame‑retardant mechanism (Figure 15i) involves lignin phosphate being pyrolyzed into carbon and cooperating with expandable graphite to form a worm‑like expanded carbon layer that isolates heat and oxygen, while gas‑phase phosphorus‑containing free radicals inhibit the combustion chain reaction. Based on the expansion‑conduction mechanism (Figure 15j), the dispersed graphite sheets formed a continuous conductive network after thermal expansion, so that the foam triggered an alarm within 7 s and continued to alarm for more than 1800 s (Figure 15k). The aromatic skeleton and high carbon content of lignin enhance the charring ability and hydrophobicity. The foam thermal conductivity is about 50 mW m^−^^1^ K^−^^1^, and its change is less than 5% at 95% relative humidity. This work realized, for the first time, the synergistic coupling of active early warning and passive flame‑retardant in bio‑based foam, and provided a reliable paradigm for a new generation of fire‑insulating materials.

### 4.3 Biomedical Engineering Application

Bio‑based PU has demonstrated remarkable structural designability and functional programmability in biomedical engineering. The microphase‑separated structure of soft and hard segments can simulate the mechanical behavior of natural tissues [90]. The long aliphatic chain of bio‑based polyol endows the material with flexibility and hydrolysis stability. Multifunctional bio‑based chain extenders can form dense hydrogen‑bond networks, thereby enhancing mechanical strength. The metabolic compatibility of bio‑based components reduces the risk of chronic inflammation induced by implants [183, 184, 185]. The above characteristics make bio‑based PU an ideal matrix for artificial ligaments, cartilage repair scaffolds, bone repair piezoelectric materials, antibacterial coatings, and wound dressings. Researchers have covalently anchored bioactive molecules to the PU backbone via molecular engineering. The introduction of dynamic non‑covalent bonds endows the material with shape memory and self‑healing properties. The crystallization behavior of bio‑based soft segment can be programmed to serve as a body‑temperature‑triggered source of deformation. Based on the above common rules, bio‑based PU is evolving from inert filler materials into platforms with reliable performance and responsive diagnostic and therapeutic capabilities.

**FIGURE 16:**
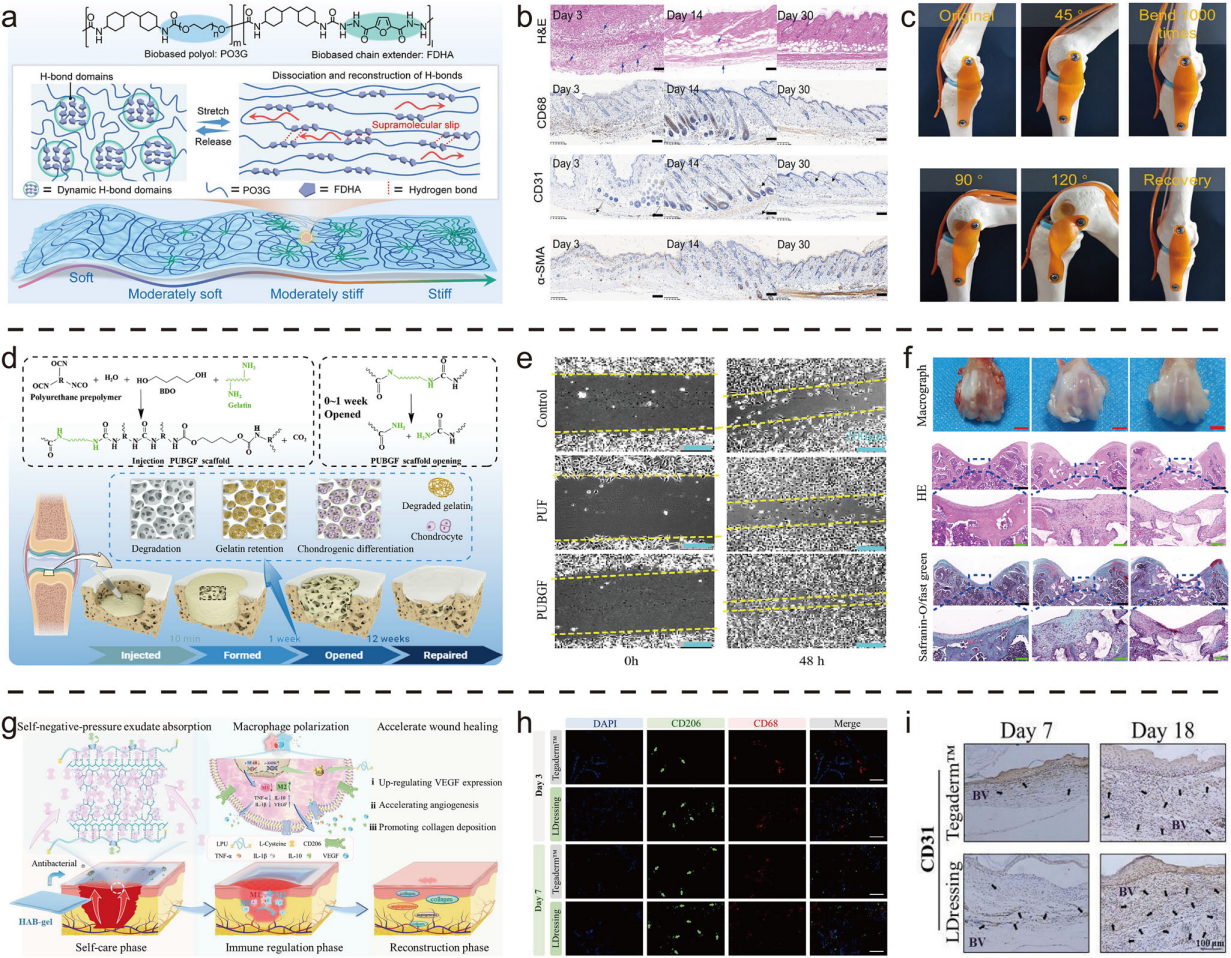
Multifunctional application examples of bio‑based polyurethane in biomedical engineering. (a) Schematic diagram of PFPU elastomer transitioning from an isolated network to a soft–hard layered bicontinuous microphase separation structure. (b) H&E staining and immunohistochemical staining of CD68, CD31, and α‑SMA in surrounding tissues after P_1_F_1_PU elastomer implantation. (c) Photographs of P_1_F_1_PU artificial ligaments after 1000 flexion cycles at different angles. (a–c) Reproduced with permission [188]. Copyright 2025, Wiley‐VCH. (d) Schematic diagram of PUBGF scaffold filling cartilage defects via in situ foaming and the dynamic microstructure evolution mechanism. (e) Scratch test results for the effect of PUBGF extract on bone marrow mesenchymal stem cell migration. (f) H&E, safranin‑O/fast green, and type II collagen staining results of cartilage defect repair at 8 and 12 weeks after PUBGF implantation. (d–f) Reproduced with permission [191]. Copyright 2024, KeAi Communications Co. (g) Mechanism of HAB‑gel dressing in chronic wound treatment through self‑pressure absorption, antibacterial activity, and immune regulation. (h) Immunofluorescence staining of CD68 and CD206 showing LPU dressing promoted M2 macrophage polarization. (i) CD31 immunohistochemical staining showing superior neovascularization density and diameter in the LPU dressing group compared to the control group. (g–i) Reproduced with permission [198]. Copyright 2023, Elsevier.

Load‑bearing soft‑tissue repair materials must have ultra‑high strength, a super‑high tensile rate, and anti‑fatigue properties. The artificial ligament is subjected to complex tension‑shear loads in the knee flexion cycle [186, 187]. Traditional PU elastomers face the contradiction between strength and toughness. The topological structure and distribution of hydrogen‑bond arrays in bio‑based soft segment are key to solving this problem. Collagen fibers and proteoglycans in natural ligaments form a bicontinuous gradient structure. Rigid collagen fiber bundles provide tensile strength through a high‑density network of hydrogen bonds. Viscoelastic proteoglycan matrix dissipates impact energy through molecular chain slip. Bio‑based PU can simulate this fiber‑matrix synergistic mechanism at the molecular scale. Chu et al. [188] used bio‑based polytrimethylene ether glycol (PO3G) as the soft segment and 2,5‑furan diformylhydrazide (FDHA) as a bio‑based chain extender to introduce hard segments. The team proposed a dynamic hydrogen‑bonding‑induced confinement effect (DHBCE) molecular engineering strategy. The transformation of the microstructure from an isolated hydrogen bond network to a soft‑hard layered bicontinuous microphase separation structure is illustrated in Figure 16a. The furan ring in FDHA and the hydrazide unit formed a multi‑level hydrogen‑bond donor‑acceptor array. The weak hydrogen bonds reversibly broke and reformed under external force, dissipating energy.

Strong hydrogen bonds acted as physical cross‐linking points to maintain network integrity. The PO3G soft segment provided the required segmental mobility for ultra‑long‑range stretching. The highest breaking strength of high‑performance bio‑based PU (PFPU) was 76.54 MPa, with a toughness of 589.75 MJ m^−^^3^ and an elongation at break of 1797.2%. A 0.1 g sample could lift a 10 kg weight. In vitro and in vivo evaluation confirmed the low immunogenicity advantage of the bio‑based components (Figure 16b). Hematoxylin‑eosin staining showed that inflammatory cell levels returned to normal 30 days after implantation. CD68‑positive macrophages returned to baseline levels in the later stages of healing. There was no significant difference in the expression of CD31 and α‑smooth muscle actin between the control group and the experimental group. The physical cross‐linking of the PO3G hydrophobic backbone and FDHA synergistically inhibited enzymatic swelling. No crack appeared in the 3D printed artificial ligament after 1000 flexion cycles from 45° to 120° (Figure 16c). The renewability of bio‑based raw materials and the reversible reconstruction of dynamic hydrogen bonds jointly ensure the long‑term stability of the implants.

The minimally invasive treatment of irregular articular cartilage defects requires the material to have both shape adaptability and a pro‑regeneration microenvironment [189, 190]. Cartilage tissue has no vascular distribution and low cell density. The implanted scaffold should guide the migration and differentiation of endogenous stem cells while filling the defect. Although injectable hydrogels can conform to the defect morphology, they often fail mechanically due to swelling. Preformed porous scaffolds are difficult to match the irregular wound boundary perfectly. The in‑situ liquid‑forming characteristics of foamed PU provide a solution to this contradiction. However, the closed‑cell structure on the surface of ordinary PU foam hinders cell growth. The introduction of bio‑based gelatin can endow the scaffold with dynamic opening properties and bioactive sites. Bahatibieke et al. [191] used polyethylene glycol and a bio‑based castor oil polyoxyethylene ether as soft segments, blending gelatin with 1,4‑butanediol as a composite foaming agent. The constructed injectable PU foam scaffold was named PU‑BDO‑Gelatin‑Foam (PUBGF). The process of in‑situ foaming of PUBGF after injection to fill the defect and establish a dynamic microstructure, where the support surface was sealed at the initial stage of forming to prevent leakage of the contents, is depicted in Figure 16d.

The gelatin chains were preferentially degraded within 2 weeks, leading to pore penetration. This confinement effect allowed gelatin degradation products to be enriched inside the scaffold, with a peak concentration exceeding that of the external environment by a factor of 3. The cell scratch test confirmed that the PUBGF extract significantly promoted the migration of bone marrow mesenchymal stem cells, and the migration rate exceeded that of the pure PU foaming group and the blank control group within 48 h (Figure 16e). In vivo repair results further verified the effectiveness of the strategy (Figure 16f). Injection of PUBGF achieved uniform filling of the new tissue in the defect area within 8 weeks. Safranin‑O/fast green staining showed that the density and distribution of the new cartilage matrix were better than those of the preformed scaffold implantation group. Type II collagen immunohistochemical staining showed that cartilage‑specific matrix deposition was more significant in the injection group. Bio‑based gelatin actively regulates the timing of permeability in the scaffold microenvironment through controllable degradation. This process can enhance the homing and differentiation of endogenous stem cells without exogenous growth factors. The hydrolytic responsiveness and spatial confinement of bio‑based components synergistically endow the scaffold with dual functions: shape adaptation and regeneration promotion.

The repair of load‑bearing bone defects involves the timing of early immune microenvironment regulation and late osteogenic differentiation. Electrical stimulation has been shown to effectively promote macrophage M2 polarization and osteogenic differentiation of bone marrow mesenchymal stem cells [192, 193]. However, in the early postoperative period, it is difficult for patients to provide electromechanical coupling stimulation through rehabilitation exercise. Although implantable electrodes can deliver in situ electrical stimulation, they must rely on an external power supply. Shape memory polymers can recover permanent morphology and release mechanical energy at physiological temperatures. The crystallization‑melting transition of bio‑based PCLs is the energy source driving deformation. By combining it with piezoelectric materials, the mechanical deformation energy can be converted into bioelectrical signals. Li et al. [194] used PCL and castor oil as mixed soft segments. Shape memory PU (SMPU) was synthesized using hexamethylene diisocyanate as the hard segment.

The multifunctionality of castor oil improves molecular chain entanglement density and energy output. The melting transition temperature of SMPU was 33°C, lower than body temperature and thus able to trigger shape recovery. The content of β phase in polyvinylidene fluoride (PVDF) nanofibers increased significantly after annealing. The piezoelectric coefficient d was 7.9 ± 0.1 pC N^−^^1^. The SMPU matrix was combined with PVDF piezoelectric fiber to achieve functional coupling between the shape‑memory effect and the piezoelectric effect. The mechanical energy released by the scaffold during shape recovery drove PVDF deformation to generate electricity, and the resulting surface electrostatic potential could actively regulate macrophage M2 polarization after surgery. When the patient recovers after exercise, the cyclic load generated by the rehabilitation training can make the stent continuously output voltage. PVDF nanofibers could deliver 2.9 V under 2% compressive strain. The metabolic compatibility of bio‑based PCL and castor oil ensures the scaffold's long‑term safety during implantation. The repair effect of the femoral defect in the AF‑SP group was significantly better than that in the non‑polarized control group. This work demonstrates that the crystallization behavior of bio‑based soft segments can convert mechanical deformation into bioelectrical stimulation. At the same time, the synergistic effect of programmable degradation and electromechanical conversion functions enabled regulation of the immune‑osteogenic cascade.

Bacterial colonization of medical catheter and wound dressing surfaces is the main cause of nosocomial infections [195, 196]. Antibiotic coatings have the dual risks of drug resistance induction and burst release toxicity. The cationic antibacterial agent polyhexamethylene guanidine (PHMG) has broad‑spectrum, high‑efficiency bactericidal activity. However, simple blending will lead to rapid leaching of PHMG in an aqueous environment. Covalent grafting of the antibacterial agent onto the polymer's main chain can inhibit leaching, but it significantly reduces mechanical properties. The rich carboxylate and hydrogen bond sites of bio‑based PU provide anchor points for electrostatic locking. Zheng et al. [197] synthesized anionic WPU with castor oil as the soft segment.

The long aliphatic chain and multi‑hydroxyl structure of castor oil endow the polymer with high cross‐linking density and hydrophobic shielding effect. Cationic PHMG was anchored in the WPU matrix by an electrostatic self‑assembly strategy. The guanidine group of PHMG formed a strong electrostatic interaction with the carboxylate group of WPU, and the amine group of PHMG also formed a dense hydrogen‑bond network with the carbonyl group of WPU. This synergistic effect enabled PHMG to maintain non‑leaching characteristics after 42 days of water immersion and 6 cycles of antibacterial testing. Quantitative analysis by UV‑visible spectroscopy showed that the leaching rate of PHMG was only 0.07 wt.%. The antibacterial activity of the composite membrane against Staphylococcus aureus and Escherichia coli exceeded 99.9%. The hydrophobic main chain derived from castor oil compressed the water absorption of the composite film from 56.4% to 6.6%. Hydrogen bonding and electrostatic cross‐linking increased the tensile strength to 30 MPa, twice that of the original film. Its toughness reached 70 MJ m^−^^3^. The 0.27 g composite membrane could withstand a load of 19,000 times its own weight. The multifunctionality of bio‑based castor oil provides the physical cross‐linking point required for mechanical strengthening, and its inherent hydrolysis stability can block the migration channel of antibacterial agents caused by water molecule penetration. The electrostatic self‑assembly strategy provides a new manufacturing paradigm for the development of non‑leaching durable antibacterial medical coatings.

Wound healing is limited by three pathological factors: exudate accumulation, bacterial biofilm formation, and stagnation of macrophage phenotype transformation [199]. Matrix metalloproteinases in the wound exudate continuously degrade the newly formed extracellular matrix. M1 pro‑inflammatory macrophages persist in the wound for a long time and cannot be transformed into M2 repair macrophages. The ideal wound dressing should have the functions of active drainage of exudate, continuous antibacterial, and immune reprogramming [200, 201]. The thiol group of bio‑based small molecule L‑cysteine (L‑Cys) can catalyze the formation of hydrogen sulfide (H_2_S) in vivo. H_2_S is an endogenous gas signaling molecule that can effectively drive macrophages to polarize from the M1 phenotype to the M2 phenotype. Zou et al. [198] synthesized bio‑based PU (LPU) with L‑Cys end‑capping. β‑Cyclodextrin was covalently incorporated into the LPU backbone as a host inclusion site for polyhexamethylene biguanide. The polyacrylamide‑grafted polyvinyl alcohol phase enabled the dressing to absorb seepage through self‑negative pressure. The hydrogel‑aerogel biphase gel (HAB‑gel) enabled the functional integration of physical adsorption and biochemical regulation.

The multiple synergistic therapeutic mechanisms of HAB‑gel are demonstrated in Figure 16g. The aerogel phase generated self‑negative pressure during the liquid absorption expansion process, with a maximum value of ‐7.25 mmHg. This negative pressure could continuously drain exudate from deep within the wound. The β‑cyclodextrin cavity‑loaded polyhexamethylene biguanide exhibited continuous release for 72 h via host‑guest interactions. Immunofluorescence staining confirmed that LPU‑based dressings significantly up‑regulated the proportion of CD206‑positive M2 macrophages, and the CD206/CD68 ratio was significantly higher than that in the commercial dressing control group on the 7th postoperative day (Figure 16h). H_2_S induced by the L‑Cys sulfhydryl group is a key signaling molecule that drives macrophage phenotype transformation. CD31 immunohistochemistry showed that the density and diameter of neovascularization in the LPU group were better than those in the control group (Figure 16i). Masson staining further showed that the collagen deposition was denser and the fibers were arranged in an orderly manner. The collagen index of the LPU group was 0.305 at 18 days, close to the normal skin level. The active thiol site of bio‐L‑Cys converts PU from an inert dressing into an active immune regulatory platform. The design of HAB‑gel provides a bio‑based integrated paradigm for sequential intervention of chronic refractory wounds.

### 4.4 Flexible Sensing and Electronic Devices

Flexible sensors and stretchable electronic devices offer broad application prospects in health monitoring and human‑computer interaction [202, 203, 204]. Such devices need to maintain a stable electrical signal output under repeated deformation. Traditional PU elastomer substrates are mostly derived from petrochemical resources, so the environmental pollution and sustainability issues associated with waste are becoming increasingly prominent. Bio‑based PU uses renewable resources as raw materials. The rich ester bonds, carbamate bonds in the molecular chain, and the designable ratio of soft and hard segments give the material adjustable mechanical properties [205]. By modifying the side chain or introducing dynamic covalent bonds, self‑healing, recyclable, and controllable degradation functions can be achieved. The low toxicity and biocompatibility of bio‑based monomers make the material suitable for long‑term skin application [102, 206, 207]. The current research is devoted to the construction of composite bio‑based PU materials that enable functional materials to exhibit high stretchability, high sensitivity, and environmental friendliness.

**FIGURE 17:**
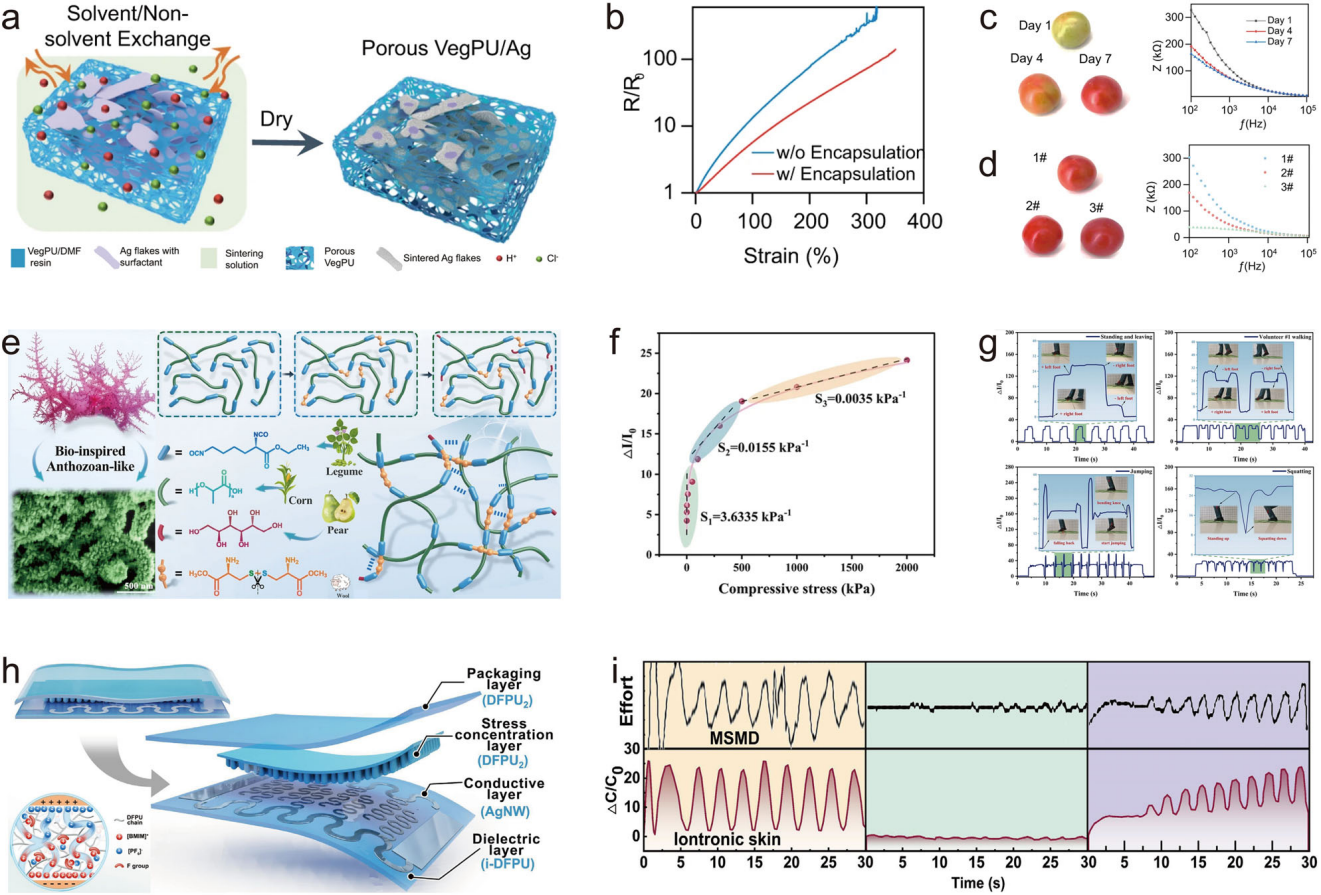
Application of bio‑based polyurethane in a flexible sensing platform. (a) Schematic diagram and microstructure of the porous VegPU matrix and silver sheet sintering. (b) Significantly reduced resistance fluctuation of the electrode during cyclic stretching by the VegPU encapsulation layer. (c) Color changes and corresponding impedance spectra of tomatoes during 7 days of storage. (d) Impedance spectra of three tomatoes with similar color but different maturity. (a–d) Reproduced with permission [210]. Copyright 2023, Springer Nature. (e) Design of a degradable omnidirectional sensing carpet based on CNTs@PANI/BDPU composites. (f) Sensitivity curve of the CNTs@PANI/BDPU pressure sensor. (g) Real‑time electrical signal response of the carpet to standing, walking, running, jumping, and squatting. (e–g) Reproduced with permission [214]. Copyright 2025, Springer Nature. (h) Integration of a dome stress concentration layer, a serpentine electrode, and an ionic decalactone‑based fluorinated polyurethane (i‑DFPU) dielectric layer in the multilayer ion‑electronic skin to amplify the capacitive response. (i) Comparison of normal respiration, apnea, and respiratory recovery waveforms recorded synchronously by the ion‑electronic skin and a medical sleep monitor. (h,i) Reproduced with permission [215]. Copyright 2024, Elsevier.

The stretchable conductor requires the elastomer to withstand large deformation while maintaining the integrity of the conductive network [208, 209]. The soft segments of bio‑based PU are mostly derived from long, fatty‑chain polyols derived from vegetable oil. The reversible dynamic bonds introduced into the hard segments can dissipate strain energy and inhibit crack propagation. This design can endow the conductor with high conductivity, high stretch, and low electrical hysteresis. Lv et al. [210] reported a printed elastic conductor based on vegetable oil‑based PU (VegPU). In this work, castor oil‑based bifunctional ricinoleic acid and epoxidized soybean oil were used to synthesize polyol. Soybean oil‑based polyol reacted with isophorone diisocyanate and dimethylglyoxime chain extender to form a dynamic cross‐linking network. The printed VegPU/silver electrode was treated with a lactic acid and chloride ion solution at room temperature. The sintering process was synchronized to achieve nonsolvent‑induced phase separation of the cured and partially removed silver surfactant (Figure 17a). Nonsolvent‑induced phase separation formed a porous network in the binder to passivate the crack tip. The tunnel barrier between silver foils was reduced due to the removal of the insulating ligand. The porous structure and sintering effect worked together to maintain electrode conductivity at 350% stretching. The VegPU encapsulation layer constrained slip in the conductive network and further improved cycle stability (Figure 17b). The conductivity of the conductor was 12833 S cm^−^^1^, and the electrical hysteresis was only 0.333 after 100% strain cycling. The VegPU‑based impedance sensor can conform to the surface of the soft gripper and follow its deformation. As the tomato skin color changed from green to red over one week, the 100 Hz impedance decreased from 327 to 164 kΩ (Figure 17c). The degradation of cell membrane capacitance led to an increase in intracellular fluid resistance and a decrease in extracellular fluid resistance. The impedance spectra of three tomatoes with similar colors but different physiological states are presented in Figure 17d, which allows a rough assessment of maturity. The bio‑based dynamic network maintains the electrode's conductive path stability under repeated deformation. This strategy provides a feasible path for integrating elastic conductors into non‑destructive monitoring for smart agriculture.

Multi‑dimensional pressure sensing requires the device to respond sensitively to external forces from different directions [211, 212, 213]. Large‑area monitoring scenarios, such as smart carpets, also require sensing coatings that are both flexible and well‑adhered to the substrate. Bio‑based PPU can achieve self‑healing and degradability through soft‑segment crystallization inhibition and dynamic disulfide bond exchange. The omnidirectional pressure‑sensing platform can be constructed by combining such a matrix with a bionic conductive microstructure. Inspired by the multi‑contact structure of coral worms, Zhang et al. [214] designed the CNTs@polyaniline (CNTs@PANI) active layer. The sensor used bio‑based degradable PU urea (BDPU) as the flexible matrix (Figure 17e). The BDPU soft segments were polylactic acid, and the hard segments contained L‑lysine diisocyanate and L‑cystine dimethyl ester as a chain extender. The CNTs@PANI pseudo‑three‑dimensional spinous structure increased the contact area by convex interlocking under micro‑stress. The sensor's sensitivity was 3.634 kPa^−^^1^ in the range of 0.05–100 kPa (Figure 17f). The glass transition temperature of BDPU was about −50°C. The activity of the soft‑segment chains at room temperature made the matrix reversibly compress the conductive network under external force. The disulfide bond in the hard segments was dynamically reorganized under thermal assistance at 35–90°C to repair the crack. The current waveforms of the carpet array for standing, walking, running, and jumping were recorded, each corresponding to a signal frequency and peak‑valley characteristics (Figure 17g). The BDPU coating maintained hydrogen‑bond anchorage with the fabric substrate after 300 bending cycles. CNTs@PANI can be recovered by degrading the matrix under alkaline conditions. The recovered material retains its sensing sensitivity after being re‑doped with hydrochloric acid. The design simultaneously solves the problems of electronic waste and the recycling of functional fillers.

Wearable respiratory monitoring devices need to be highly sensitive to capture subtle thoracic deformation. Patients with central sleep apnea often experience night sweats. Therefore, the matrix material must be sweat‑resistant and biocompatible [216]. The alkyl side chains of bio‑based polyester diols can reduce the packing density of molecular chains. Their combination with fluorine‑containing hard segments can simultaneously achieve sweat resistance and ion‑trap effects. Li et al. [215] used polyester diol obtained by ring‑opening polymerization of δ‑decalactone as the soft segment. The hard segments were introduced with 2,2,3,3,4,5,5‑octafluoro‑1,6‑hexanediol and diphenylmethane diisocyanate. The multi‑layer architecture of the ion electronic skin, which included a decalactone‑based fluorinated PU (DFPU) package layer, a dome stress concentration layer, a serpentine silver nanowire electrode, and an i‑DFPU dielectric layer, is shown in Figure 17h. The height and bottom diameter of the dome array were about 100 µm.

The sensor could concentrate thoracic expansion forces onto the sensing area, thereby amplifying capacitance changes. The alkyl side chains of the soft segment promote loose molecular packing. The cation in the [BMIM]^+^[PF]^−^ ionic liquid forms reversible ion‑dipole interactions with the fluorine groups of the hard segments. The fluorine group captured the cation when standing. When pressed, the trap was destroyed, and the ions were released to migrate to the electrode. The contact angle of DFPU to artificial sweat was 114.5°. The mass remained almost constant after 4 days of soaking. The proliferation trend of L‑929 cells in i‑DFPU medium was consistent with that of the medical polyethylene control group. The synchronous recordings of respiration from a suspected patient by the ion electronic skin and a medical sleep monitor were compared (Figure 17i). The capacitance signal fluctuated regularly during normal breathing. When apnea occurred, the thoracic signal disappeared, and there was no curve peak. The signal intensity of spontaneous breathing recovery gradually increased. At the same time, the device still maintained signal stability in the sweat state. The bio‑based soft segment and the fluorine‑based trap effect increased the sensitivity to 132.2 kPa^−^^1^. This strategy provides a highly reliable wearable solution for clinical apnea warning.

### 4.5 Energy Storage and Conversion Devices

Energy storage and conversion devices are rapidly advancing toward greater flexibility, integration, and sustainability. Wearable electronics, unmanned monitoring terminals, and distributed sensor networks impose stringent requirements on lightweight, long‑life energy‐supply units [217, 218, 219]. Traditional energy devices rely on petroleum‑based dielectrics or metal current collectors to build rigid architectures. Such systems are difficult to withstand repeated deformation and to repair. Bio‑based PU is an ideal alternative matrix due to its highly tunable molecular structure and the ease with which dynamic bonds can be introduced [220, 221]. The glass transition temperature and dielectric relaxation behavior of the material can be adjusted by changing the composition and proportion of the soft segment. The hard‑segment microphase endows the material with mechanical robustness and self‑healing potential through hydrogen bonds or dynamic covalent bonds. The multi‑functionality and aromatic conjugated structure of biomass raw materials further expand the design space of photothermal conversion and electrical response. These intrinsic advantages make bio‑based PU valuable for supercapacitors, dielectric energy storage capacitors, triboelectric nanogenerators (TENG), and photothermoelectric conversion systems.

**FIGURE 18:**
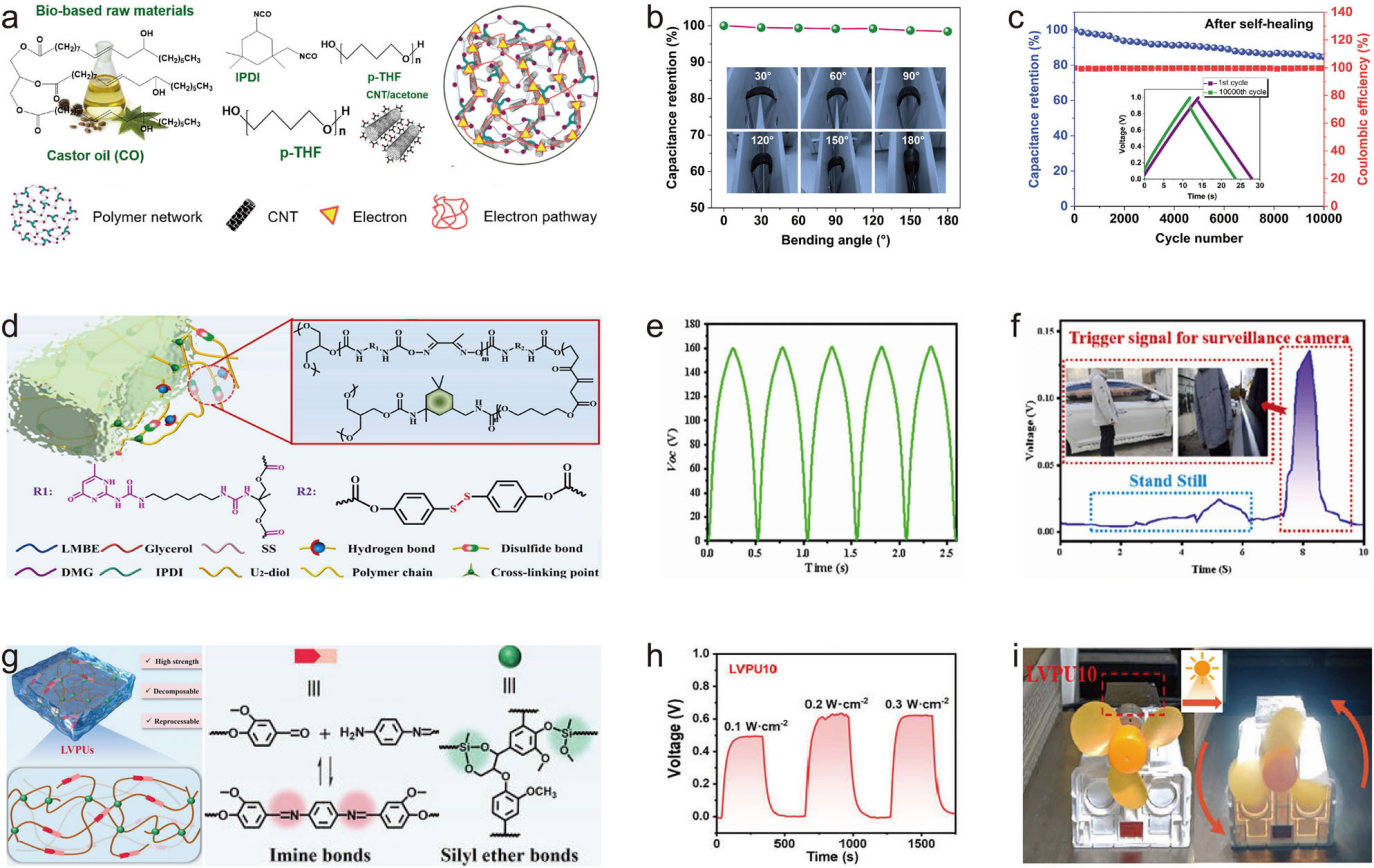
Key structural design, performance, and practical application demonstration of bio‑based polyurethane materials in energy storage and conversion devices. (a) Synthesis of castor oil‑based polyurethane/carbon nanotube composites and display of the self‑healing conductive network structure. (b) Capacitance retention of flexible supercapacitors at various bending angles. (c) Capacitance retention and Coulombic efficiency curves of the device after 10 000 charge–discharge cycles. (a–c) Reproduced with permission [225]. Copyright 2024, Elsevier. (d) Synthesis of a low‑temperature self‑healing polyurethane elastomer from itaconic acid and sebacic acid. (e) Open‑circuit voltage of TENG at room temperature. (f) Demonstration of TENG‑triggered camera monitoring in vehicle sentinel mode. (d–f) Reproduced with permission [227]. Copyright 2024, Elsevier. (g) Dynamic network structure diagram of LVPUs. (h) Output voltage of the photo‑thermal‑electric conversion system based on LVPUs under different light intensities. (i) Photographs of the LVPUs light‑heat‑electricity energy conversion system driving a micro‑fan in continuous operation. (g–i) Reproduced with permission [231]. Copyright 2025, Wiley‐VCH.

Supercapacitors require current collectors with high conductivity, flexibility, and long‑term reliability. The flexible device must maintain the functioning of its conductive path after repeated bending or accidental damage [222, 223, 224]. Bio‑based PU can rely on the reconstruction of a dynamic reversible network to achieve self‑healing of the damaged interface. Vu et al. [225] constructed a disulfide‑containing self‑healing PU matrix from castor oil and poly(tetrahydrofuran) mixed polyols. The design logic involved the bio‑based long, fatty‑chain polyol copolymerizing with poly(tetrahydrofuran) to regulate the cross‐linking density and provide the chain segments with sufficient mobility (Figure 18a). CNTs were uniformly dispersed in the matrix to form a percolation conductive network. Dynamic disulfide bonds and dense hydrogen bonds synergistically drove the crack interface to rebond under thermal excitation. The conductivity of the bio‑based composite material reached 1.10 S m^−^^1^, and the electrical self‑healing efficiency was about 98% within 70 s at 100°C. When used as a flexible solid‑state supercapacitor current collector, the device's area‑specific capacitance was 44.7 mF cm^−^^2^. The Ragone curve showed that the device exhibited an area energy density of 0.22 µWh cm^−^^2^ and a power density of 0.09 mW cm^−^^2^ (Figure 18b). The specific capacitance recovery rate reached 92.4% after seven cutting/healing cycles (Figure 18c). The viscoelasticity of the bio‑based substrate promotes the close adhesion of the electrode and the electrolyte under dynamic deformation. The castor oil‑derived PU network can buffer the volume change of the electrolyte during charging and discharging. This work demonstrates the feasibility of a bio‑based self‑healing current collector for long‑life, flexible energy storage devices.

Dielectric energy storage capacitors require both a high dielectric constant and a high breakdown strength of dielectric materials. Although the traditional biaxially oriented polypropylene has a high breakdown field strength, its dielectric constant is only about 2.2. The dielectric constant of fluorine‑containing ferroelectric polymers is considerable but accompanied by high polarization hysteresis loss. Bio‑based poly(hydroxyurethane) (PHU) can simultaneously achieve high energy storage density and high discharge efficiency by a dense arrangement of polar groups and precise regulation of the glass transition temperature. Le Goupil et al. [226] synthesized a fully bio‑based PHU dielectric using erythritol dicarbonate and a bio‑based diamine. Erythritol dicarbonate was derived from natural sugar alcohols and carried two hydroxyl groups and two carbamate polar groups per repeating unit. The dielectric constant of the molecular structure was as high as 8.4 at 1 kHz. The intrinsic breakdown strength of PHU was greater than 400 MV m^−^^1^. The linear dielectric behavior, in combination with the high breakdown field, enabled the discharge to reach an energy density exceeding 6 J cm^−^^3^. The discharge efficiency of the bio‑based PHU reached 85% at the breakdown field strength and increased to 91% at 0.5 times the breakdown field strength. This performance is comparable to that of commercial biaxially oriented polypropylene and significantly exceeds the upper efficiency limit of fluorinated ferroelectric polymers. The rigid skeleton of the erythritol alicyclic group and the carbamate hydrogen bond network jointly suppressed the high‑field conductive loss. The fully bio‑based synthetic route simultaneously avoids the toxicity of isocyanates and reduces the life‐cycle carbon emissions of capacitors.

A TENG converts environmental mechanical energy into electrical energy by contact electrification and electrostatic induction. Outdoor application scenarios require the friction layer material to maintain flexibility and damage self‑healing ability at low temperatures. Bio‑based PU can reduce the glass transition temperature to a level well below the operating temperature by copolymerizing a long‑chain dibasic acid with a polyol. Wang et al. [227] synthesized hydroxyl‑terminated prepolymer by polycondensation of bio‑based monomers such as itaconic acid and sebacic acid. The whole process involved introducing isophorone diisocyanate and an aromatic disulfide system into the prepolymer as soft segments (Figure 18d). The bio‑based, long aliphatic chain lowered the glass transition temperature to −30°C. The polymer network synchronously integrated the quadruple hydrogen bonds and disulfide bonds of ureidopyrimidinone. The multiple dynamic bonds endowed the elastomer with 1100% elongation at break and 84% tensile‑strength self‑healing efficiency at −10°C. The single‑electrode TENG based on the elastomer had an open‑circuit voltage of 160 V at a 2 Hz operating frequency (Figure 18e). The abundant hydroxyl and carbamate groups on the surface of bio‑based PU enhance its electron‑donating ability with polytetrafluoroethylene. In vehicle sentinel mode, the device generated a 0.12 V trigger signal and activated the monitoring camera when a volunteer approached (Figure 18f). The elastomer's intrinsic low‑temperature flexibility ensures that the device stably drives the LED array at −40°C. The recovery rate of the output voltage was 98% after repair at −10°C for 12 h. The bio‑based dynamic network synchronously restores effective contact between the friction and electrode layers by reconstructing the interface.

An ideal self‑powered photothermal conversion device should have strong photothermal and thermoelectric conversion capabilities, stable mechanical properties, long‑term operational stability, and good environmental tolerance [228, 229]. Lignin is a natural polyphenol polymer with abundant reserves. Its aromatic conjugated structure endows the material with broadband spectral absorption and photothermal conversion capabilities [230]. Huang et al. [231] prepared multifunctional elastomers lignin‐vanillin‐based PU elastomer (LVPUs) by introducing lignin and double dynamic covalent bonds into PU networks via a dynamic locking strategy. The network structure design involved a silicon ether bond locking the imine bond activity (Figure 18g). The bio‑based vanillin‑derived diimine chain extender and the lignin rigid skeleton jointly formed dense hydrogen‑bond networks and covalent cross‐linking sites. The intrinsic *π*‐‑*π* conjugation of lignin made the surface temperature of LVPUs reach 77°C under the irradiation of 0.1 W cm^−^^2^. The output voltage of the photothermoelectric conversion system based on LVPU10 increased from 0.5 to 0.6 V with increasing optical power density (Figure 18h). The practical load capacity was verified by driving a small fan, which started rotating within 30 s under 1 sun irradiation (Figure 18i). The lignin endowed the elastomer with UV shielding efficiency exceeding 99% and a tensile strength of 26.2 MPa. Bio‑based components integrate mechanical protection, UV shielding, and energy supply into a single material platform. This design provides a lightweight solution for energy self‑supply and self‑protection for unmanned systems in field environments.

## 5 Conclusion and Outlook

The development of bio‑based PU has progressed beyond the simple replacement of petroleum‑based feedstocks toward a stage of systematic structural engineering and functional integration. However, it is essential to recognize that a bio‑based origin alone does not guarantee sustainability; comprehensive LCA, energy‑efficient processing, and effective end‑of‑life management are equally critical [232]. This review has systematically summarized the chemical basis, structural engineering design principles, and frontier applications of bio‐based PU. The extensive utilization of renewable raw materials has established a biomass‐based supply system for polyols, internal emulsifiers, and isocyanates, while the non‐isocyanate route further circumvents the use of toxic precursors. Segment engineering enables precise control over the degree of microphase separation by tuning the chemical composition and compatibility of the soft and hard segments. Studies of aggregation structures have revealed the quantitative influence of hydrogen‐bond arrays and dynamic bond distribution on mechanical response and energy dissipation. The introduction of dynamic covalent bonds overcomes the intrinsic irreversibility of traditional thermosetting networks. While a single dynamic bond system endows the material with self‐healing and reprocessing capabilities, a multi‐dynamic‐bond coordination strategy simultaneously enhances mechanical performance and the rate of network topology rearrangement [233]. Nanomaterials strengthen filler‐matrix interfacial coupling through the abundant binding sites provided by the bio‐based matrix and impart diverse functionalities. These advances in structural engineering have enabled bio‐based PU to demonstrate compelling advantages in smart coatings, fire protection, biomedical engineering, flexible sensing, and energy devices.

### 5.1 Critical Assessment of Current Challenges

#### 5.1.1 Sustainability Assessment Beyond Feedstock Origin

The frequent characterization of bio‑based PUs as sustainable in the literature warrants critical examination. While renewable feedstock utilization represents a meaningful step toward reducing fossil resource dependence, a bio‑based origin alone is insufficient to guarantee environmental sustainability. A comprehensive life‑cycle assessment must account for multiple factors beyond feedstock renewability [234]. First, agricultural impacts include land‐use change, water consumption, fertilizer and pesticide application, and potential competition with food production when using edible oil feedstocks such as soybean, palm, and rapeseed oils are employed. Microalgal oils and non‑edible oil crops, including jatropha and castor, partially mitigate food competition but introduce their own cultivation challenges. Second, the processing energy intensity of chemical modification must be considered: processes such as epoxidation, hydroformylation, and ozonolysis often require substantial energy inputs and may involve hazardous organic solvents, contributing significantly to the overall carbon footprint [235]. Third, use‐phase durability should also be evaluated: materials with insufficient service life may necessitate frequent replacement, potentially negating any cradle‐to‐gate environmental benefits. Furthermore, the limited number of published techno‐economic and life‐cycle assessment studies on bio‐based PUs highlights a critical gap in the current literature. We strongly recommend that future research publications include, at minimum, a qualitative discussion of life‐cycle considerations and, ideally, quantitative data to substantiate sustainability claims. Practical strategies for improving sustainability include adopting green chemistry principles for chemical modification steps, utilizing non‐edible or waste‐derived feedstocks, and implementing process intensification to reduce energy demand.

#### 5.1.2 End‑of‑Life Management: Biodegradability versus Chemical Recyclability

It is essential to distinguish between biodegradability and chemical recyclability, as these represent fundamentally different end‑of‑life pathways with distinct requirements and implications. Biodegradability refers to the breakdown of a material by microorganisms under specific environmental conditions into CO_2_, water, and biomass. Typical environments include soil burial, marine conditions, and industrial composting, with evaluation conducted according to standardized protocols such as ISO 14855 for aerobic composting, ASTM D6400 for compostable plastics, and EN 13432 for packaging waste [236, 237]. Chemical recyclability refers to the depolymerization of the polymer network back to monomers or well‑defined oligomers through chemical reactions, including hydrolysis, alcoholysis, aminolysis, and acidolysis, for subsequent re‑polymerization into virgin‑quality materials. Biodegradability is advantageous for single‑use applications where post‑use collection is impractical, such as agricultural mulch films and disposable packaging, although biodegradation rates for cross‐linked PU networks are typically slow under ambient conditions. Chemical recyclability can preserve material value by recovering reusable monomers or oligomers, but requires dedicated collection infrastructure, energy input for depolymerization, and separation and purification of recovered monomers [238]. The choice between these end‑of‑life pathways, or their intelligent combination, should be guided by the intended application, expected service lifetime, and available waste management infrastructure. Furthermore, the long‑term stability of dynamic networks under realistic service conditions, including performance degradation after repeated repair cycles and the reversibility of bonding in complex chemical environments, must be systematically evaluated to ensure that recyclability claims of recyclability are validated under practical conditions [239].

#### 5.1.3 From Laboratory to Industry: Processing and Scale‑Up Considerations

The translation of academic bio‐based PU research to industrial production faces several critical challenges that are often underappreciated in the literature: (1) Kinetics and viscosity engineering: Academic syntheses typically proceed in low‐volume glass reactors (100–500 mL) with extended reaction times (4–24 h) and manual reagent addition, whereas industrial continuous processing (tubular reactors, CSTR cascades) demands rapid, controllable kinetics with residence times of 5–30 min [240]. The lower reactivity of secondary hydroxyl groups in many bio‐based polyols compared to petroleum‐based polyether polyols necessitates optimized catalyst systems and potentially elevated temperatures. Reactive diluent strategies, temperature‐controlled viscosity management, and process‐compatible formulations represent key engineering approaches for bridging this gap. (2) Purification bottlenecks: While laboratory‐scale purification can employ labor‐intensive techniques such as precipitation, dialysis, or column chromatography, industrial‐scale production requires rapid, high‐throughput purification methods [241]. I Inadequate removal of residual organometallic catalysts (organotin, Bi, Zn compounds at ppm levels), malodorous fatty acid derivatives, and unreacted monomers leads to VOC entrapment and compromised long‐term durability, rendering products unsuitable for sensitive applications (automotive interiors, medical devices, food contact). Thin‐film evaporation, wiped‐film molecular distillation, steam stripping, and solid‐phase adsorption represent scalable purification alternatives. (3) Feedstock standardization: As discussed in Section 2.1, the inherent variability of natural feedstocks necessitates robust quality control protocols, potential blending strategies, and real‐time process analytics to ensure reproducible material properties at production scale.

#### 5.1.4 Technology Readiness Assessment

To provide a realistic perspective on the current state of bio‐based PU technology, we categorize the major application domains according to their technological maturity: (1) Near‐term realistic applications include protective coatings and flame‐retardant foams, which benefit from established industrial PU manufacturing infrastructure, demonstrated commercial products (castor oil‐based coatings from BASF, soybean oil‐based flexible foams from Ford and John Deere, lignin‐based rigid foams for building insulation), and relatively straightforward performance requirements [111, 112]. (2) Medium‐term promising applications include biomedical scaffolds for tissue engineering and flexible strain/pressure sensors, which have demonstrated compelling laboratory‐scale results and, in some cases, preclinical validation (in vitro cytotoxicity, in vivo biocompatibility studies), but require further regulatory approval (ISO 10993 biocompatibility testing, FDA/CE marking), long‐term degradation data under physiological conditions, and extensive field testing before widespread clinical or consumer adoption [242]. (3) Long‐term aspirational applications include fully autonomous self‐healing systems capable of operating without external stimuli (heat, light, or chemical triggers), bio‐integrated energy devices that couple energy harvesting and storage with biological or wearable systems, and truly closed‐loop material systems in which products are designed from the outset for complete chemical recovery and infinite re‐manufacturing cycles [243]. These application concepts represent exciting research frontiers but currently face significant gaps in fundamental understanding and formidable engineering challenges. In addition, the quantitative structure‐activity relationships between kinetic behavior and macroscopic properties remain insufficiently understood, particularly in complex response mechanisms involving multi‐physics coupling, where more accurate in situ characterization techniques and multi‐scale simulation methods are needed for in‐depth analysis [244, 245]. Furthermore, current demonstrations of controlled degradation or closed‐loop recovery have mostly been conducted in idealized chemical environments, and their relevance to real biodegradable environments or industrial recovery processes remains to be verified [246, 247].

### 5.2 Future Research Directions

**FIGURE 19:**
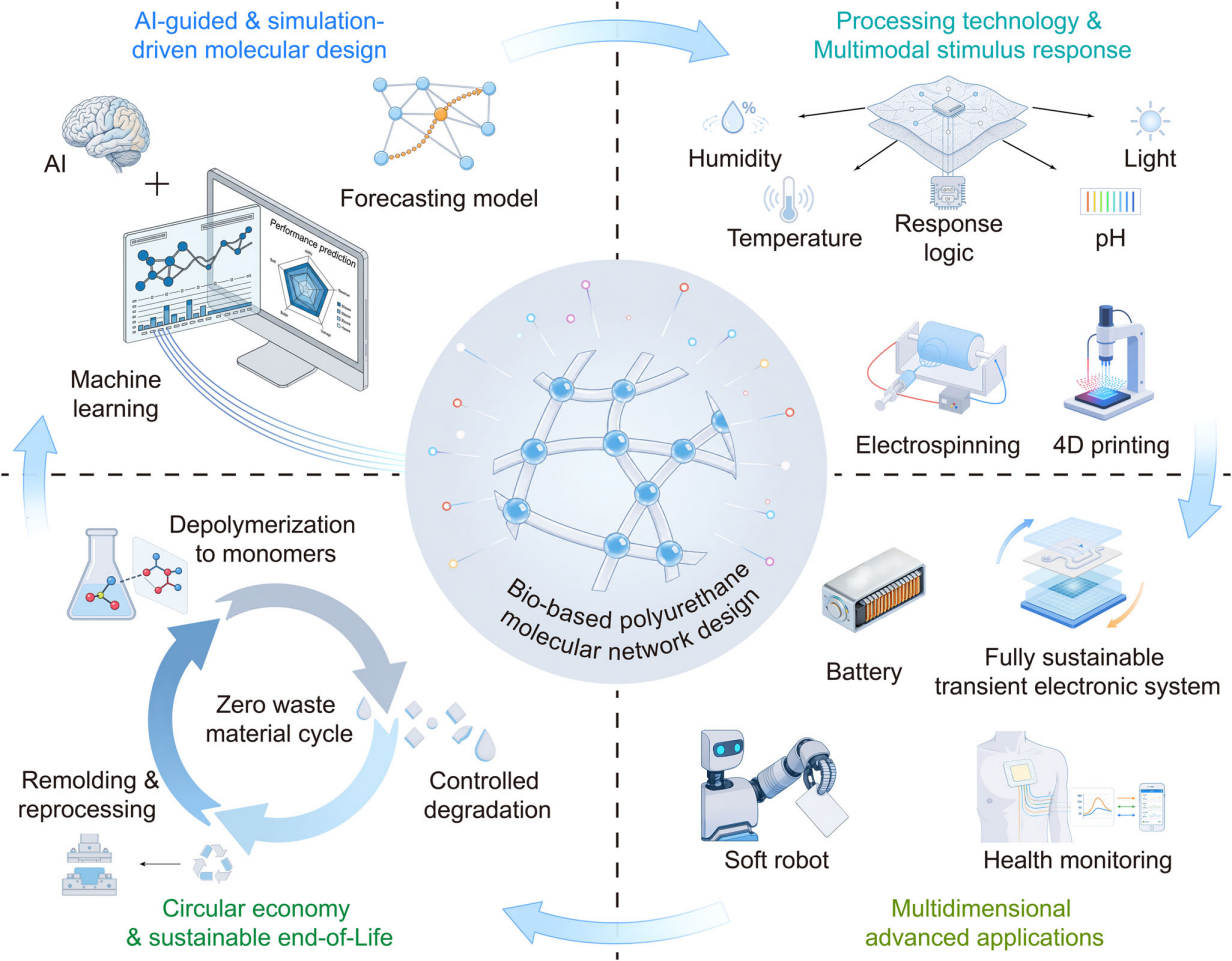
Prediction of the future development direction of bio‐based polyurethane.

Future research on bio‐based PU is expected to place even greater emphasis on deep mechanistic understanding of structure‐property relationships, precise functional design, and the construction of closed‐loop systems for full life‐cycle sustainability. The following research directions merit particular attention (Figure 19).

1. Data‑driven material design and precision manufacturing: Machine learning and artificial intelligence can integrate databases encompassing bio‐based raw material structures, dynamic bonding reaction kinetics, and final material performance to establish predictive models spanning from molecular structure to macroscopic properties. Based on such models, researchers can inversely design optimal material formulations that meet specific application requirements [248]. Advanced manufacturing technologies such as 4D printing and electrospinning have already enabled the controllable fabrication of materials with spatially heterogeneous compositions and structural gradients, which can address the needs of personalized biomedical implants and are equally suited for manufacturing flexible electronics with complex geometries.
2. Advances in environmental adaptability and intelligent responsiveness: Next‐generation materials must possess multi‐modal perception and autonomous adaptability [249]. Dynamic networks should be capable of responding to multiple stimuli simultaneously, including temperature, humidity, light, and specific biomolecules, with response logic resembling that of Boolean logic gates. Smart dressings could autonomously adjust the release rate of antibacterial agents in response to changes in pH and enzyme concentration within the wound microenvironment, while also regulating the timing of growth factor delivery. The outer skin of a soft robot could adjust stiffness and damping in real time in response to external mechanical loads and electrical feedback.
3. Extreme environmental tolerance and functional reliability: The application of materials in harsh environments, such as cryogenic, deep‐sea, high‐radiation, and high‐load‐cycle conditions, needs to be expanded. This requires fundamental research on the evolution of dynamic networks under extreme conditions [250, 251]. Researchers can strengthen interfacial interactions through molecular design, introduce energy‐dissipation mechanisms based on sacrificial bond topology design, and incorporate specialized functional nanomaterials to collectively enhance the mechanical integrity, functional retention rate, and self‐healing capability of materials in extreme environments.
4. Cross‑scale collaboration and system integration: Future breakthroughs will increasingly depend on the collaborative design of materials, devices, and systems, requiring optimization not only of the materials themselves but also of their integration with functional units such as energy‐harvesting and storage modules, signal‐processing circuits, and wireless transmission components. For example, researchers could develop fully sustainable, degradable transient electronic systems that integrate a bio‐based PU dielectric layer, a stretchable conductor, and a self‐healing encapsulation layer, which degrade harmlessly after completing health‐monitoring or environmental‐sensing tasks.
5. Advancing closed‐loop design for the circular economy: Future research needs to develop hierarchical response and recovery systems aligned with the product life cycle. This goal extends beyond the introduction of a single dynamic bond; it requires maintaining a high degree of stability during service while enabling, under specific trigger conditions such as particular enzymes, pH levels, or sequential chemical stimuli, the complete depolymerization of the network into original monomers or high‐value oligomers. Concurrently, pathways for co‐processing bio‐based PU waste with other biomass or polymer waste streams should be explored to upcycle these wastes into new materials, thereby achieving a truly zero‐waste material cycle.

## Author Contributions

D.Z. and C.Z. supervised the project. X.L. and H.D. co‐wrote the text. X.L. and H.D. drew the schematic diagrams. S.Y., J.H. and Z.C. were involved in the collection of topic literature and the organization of table data. D.Z., Z.C., X.L., and C.Z. made the guidance amendments to the pictures and text and revised the manuscript. All authors read and approved the final submitted manuscript.

## Conflicts of Interest

The authors declare no conflicts of interests.

## Data Availability

The data that support the findings of this study are available on request from the corresponding author. The data are not publicly available due to privacy or ethical restrictions.
